# Supplementary material for: Visible-Light-Promoted Tandem Skeletal Rearrangement/Dearomatization of Heteroaryl Enallenes
Source: Molecules. 2024 Jan 25;29(3):595. doi: 10.3390/molecules29030595 (PMC10856172; doi:10.3390/molecules29030595)
Supplement: Supplementary file 1 [file molecules-29-00595-s001.zip › Supplementary.pdf]

Supporting Information for

# Visible-light promoted tandem skeletal rearrangement/ dearomatization of heteroaryl- enallenes

Maurizio Chiminelli, Gabriele Scarica, Andrea Serafino, Luciano Marchiò, Rosanna Viscardi and Giovanni Maestri

# Table of contents


| | |
|---|---|
| **Details on the experimental setup** | **3** |
| **Optimization experiments** | **6** |
| **Synthesis of substrates** | **7** |
| **Copies of NMR spectra** | **13** |
| **XYZ coordinates** | **46** |
| **Comprehensive Table in AU** | **55** |

# Details of the experimental setup

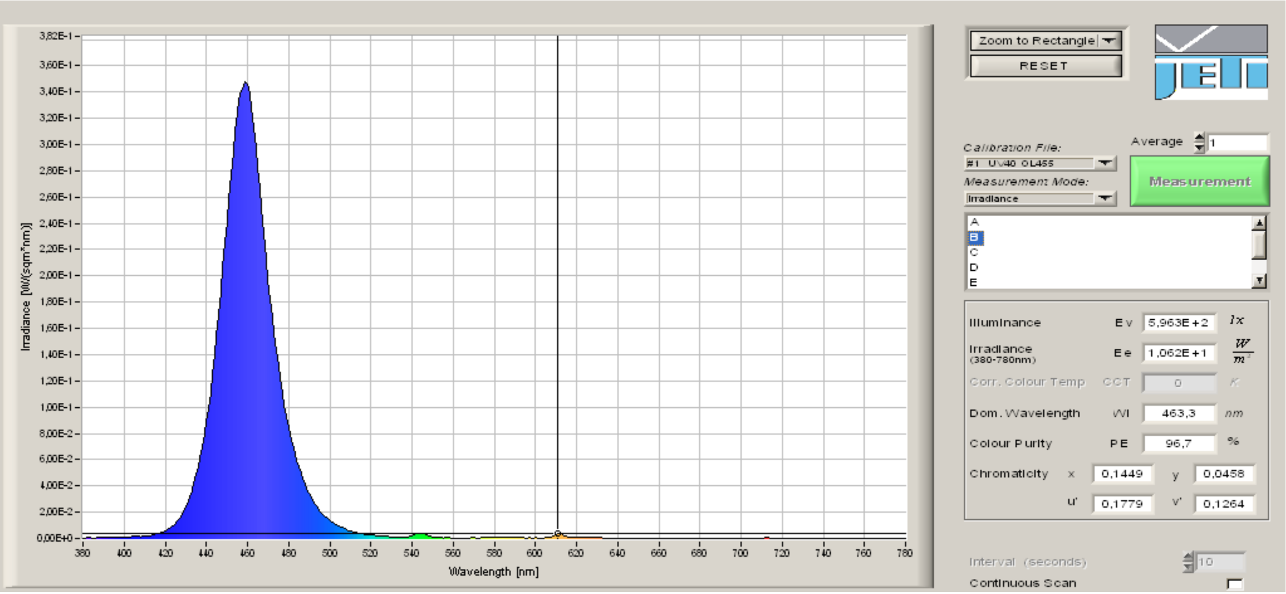


Measured emission of the adopted LED strip (above) and photo of the photoreactor (below).

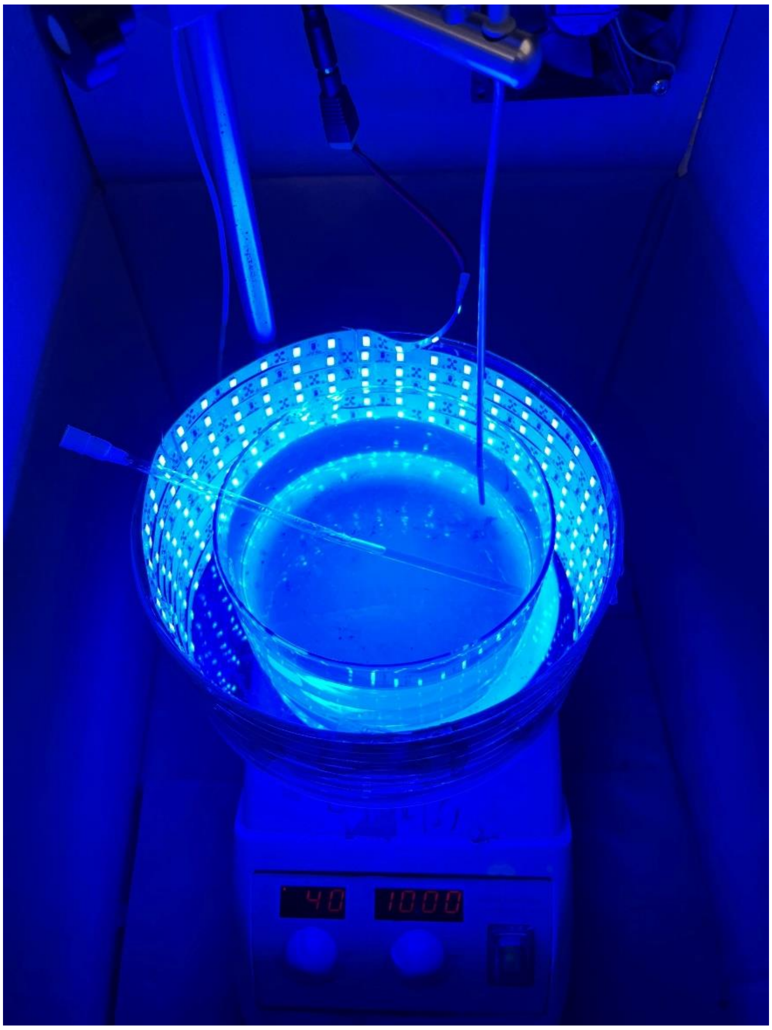

## UV-VIS ABSORPTION OF 1a AND SILICON OIL

Substrate **1a** and silicon oil were dissolved in MeCN to a dilution of $10^{-3}$ M. Then, both solutions were diluted to $10^{-5}$ M, and UV-vis absorption spectra were recorded using a Lambda 750 spectrophotometer (Perkin Elmer).
Substrate **1a** starts absorbing below 370 nm, far from the emission of the LED strip. This is consistent with the absence of detection of the desired product performing the reaction without the photocatalyst. On the other hand, silicon oil has no significant absorption below 300 nm, so it does not interfere with the emission of the blue LEDs.

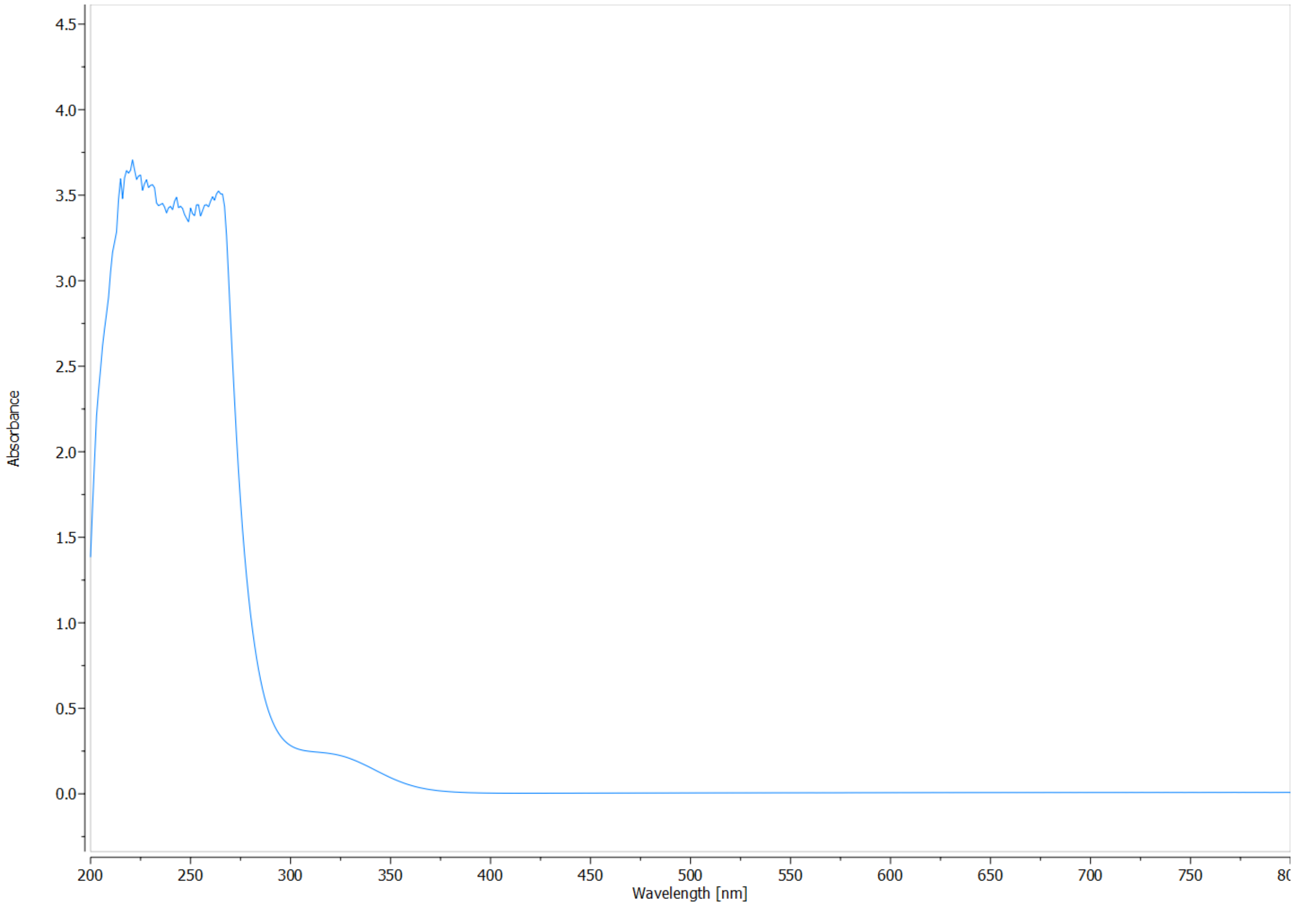


*Figure 1: UV spectra of **1a***

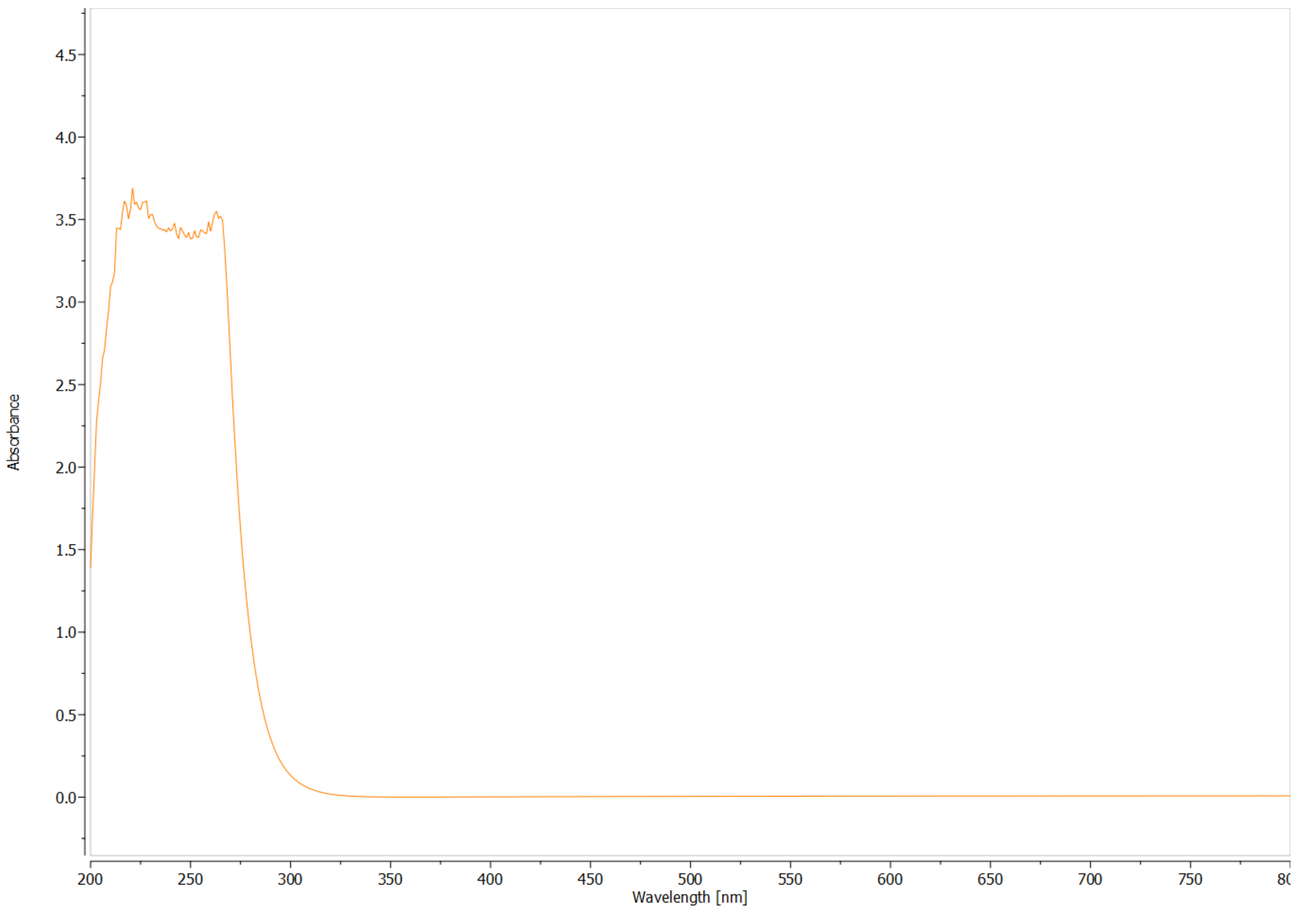


*Figure 2: UV spectra of silicon oil*

# Optimization experiments

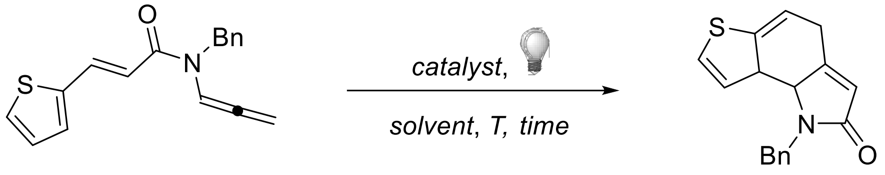


**1a** **2a**

| Entry | Catalyst (1 mol %) | Solvent (0.1 M) | Temperature (° C) | Time (h) | Yield of **2a**[a] [%] |
|---|---|---|---|---|---|
| 1[b] | $Ir(ppy)_3$ | DMF | 25 | 3 | 31 |
| 2 | $Ir(ppy)_3$ | DMF | 25 | 3 | 32 |
| 3 | $Ir(ppy)_3$ | DMF | 25 | 18 | 32 |
| 4 | $Ru(bpy)_3Cl_2$ | DMF | 25 | 18 | 24 |
| 5 | $Ir(p\text{-}F\text{-}ppy)_3$ | DMF | 25 | 18 | 37 |
| 6 | $(Ir[dF(CF3)ppy]_2(dtbbpy))PF_6$ | DMF | 25 | 18 | 19 |
| 7 | Thioxanthen-9-one[c,e] | DMF | 25 | 18 | 14 |
| 8 | Benzophenone[c,e] | DMF | 25 | 18 | 11 |
| 9 | $Ir(p\text{-}F\text{-}ppy)_3$ | Toluene | 25 | 18 | 26 |
| 10 | $Ir(p\text{-}F\text{-}ppy)_3$ | Toluene/DCM 1:1 | 25 | 18 | 50 |
| 11 | $Ir(p\text{-}F\text{-}ppy)_3$ | DCM | 25 | 18 | 60 |
| 12 | $Ir(p\text{-}F\text{-}ppy)_3$ | MeCN | 25 | 18 | 0[f] |
| 13 | $Ir(p\text{-}F\text{-}ppy)_3$ | $MeCN/CHCl_3$ 7:3 | 25 | 18 | 24 |
| 14 | $Ir(p\text{-}F\text{-}ppy)_3$ | Chlorobenzene | 25 | 18 | 40 |
| 15 | $Ir(p\text{-}F\text{-}ppy)_3$ | THF | 25 | 18 | 24 |
| 16 | $Ir(p\text{-}F\text{-}ppy)_3$ | $CHCl_3$ | 25 | 18 | 33 |
| 17 | $Ir(p\text{-}F\text{-}ppy)_3$ | DCE | 25 | 18 | 35 |
| 18 | $Ir(p\text{-}F\text{-}ppy)_3$ | DCM [0.05 M] | 25 | 18 | 53 |
| 19 | $Ir(p\text{-}F\text{-}ppy)_3$ | DCM [0.01 M] | 25 | 18 | 5 |
| 20 | $Ir(p\text{-}F\text{-}ppy)_3$[e] | DCM | 25 | 18 | 58 |
| 21 | $(Ir[dF(CF3)ppy]_2(bpy))PF_6$[e] | DCM | 25 | 18 | 40 |
| 22 | $(Ir[dF(CF3)ppy]_2(dtbbpy))PF_6$[e] | DCM | 25 | 18 | 37 |
| 23 | $Ir(p\text{-}F\text{-}ppy)_3$ | DCM | 10 | 9 | 0[f] |
| 24 | $Ir(p\text{-}F\text{-}ppy)_3$ | DCM | 40 | 3 | 66 |
| 25 | $Ir(p\text{-}F\text{-}ppy)_3$ | DCM [0.2 M] | 40 | 3 | 39 |
| 26 | - | DCM | 25 | 120 | 0[f] |
| 27[d] | $Ir(p\text{-}F\text{-}ppy)_3$ | DCM | 25 | 120 | 0 |

*[a] $^1$H NMR yield using 2,2′-Bipyridyl as internal standard, [b] with freeze-pump-thaw for removing $O_2$, [c] 10 mol %, [d] without light, [e] purple light, [f] degradation of SM.*

*Best conditions*

# Synthesis of substrates

Synthesis of esters *via* Heck coupling

| Precursor of | Yield |
|---|---|
| **1a-d** | 92% |
| **1m** | 89% |
| **1n** | 40% |

Synthesis of esters *via* Wittig olefination

| Precursor of | Yield |
|---|---|
| **1e, 1g, 1h, 1i** | 90% |
| **1j, 1k** | 96% |
| **1l** | 70% |

Hydrolysis of esters

| Precursor of | Yield |
|---|---|
| **1a-d** | 90% |
| **1e, 1g, 1h, 1i** | 70% |
| **1j, 1k** | 91% |
| **1l** | 87% |
| **1m** | 86% |
| **1n** | 88% |

Synthesis of enynes

| Precursor of | Yield |
|---|---|
| **1a** | 85% |
| **1b** | 83% |
| **1c** | 58% |
| **1d** | 53% |
| **1e** | 80% |
| **1f** | 61% |
| **1g** | 74% |
| **1h** | 89% |
| **1i** | 41% |
| **1j** | 98% |
| **1k** | 63% |
| **1l** | 66% |
| **1m** | 80% |
| **1n** | 89% |

## A) Synthesis of secondary amines

Synthesis of secondary amines via nucleophilic substitution (precursor of **1a, 1e, 1f, 1i, 1j, 1l, 1m, 1n, 1o, 1p**)

In a round-bottom flask equipped with a magnetic stirring bar, propargyl bromide (1 equiv.) was added at 0 °C to primary amine (6 equiv.), and the resulting solution was stirred for 18 h at room temperature. After complete conversion as monitored by TLC, the mixture was quenched with a saturated $NaHCO_3$ solution and extracted with $Et_2O$ (3 times). The combined organic phase was washed with brine, dried over $Na_2SO_4$, and concentrated under reduced pressure. The crude was finally purified by chromatography on silica gel (*n*-hexane/EtOAc gradient) to afford the desired product (Yield = 95%) or used without purifications for the synthesis of **1i**.

Synthesis of secondary amines via reductive amination (precursor of **1b, 1c, 1g, 1h, 1k**)

One drop of AcOH was added to a solution of propargyl amine (1 equiv.) and aryl aldehyde (1.08 equiv.) in MeOH (0.6 M). The resulting mixture was then stirred for 18 h at room temperature. $NaBH_4$ (1.5 equiv.) was added at 0 °C, and the solution was then stirred for 1 h prior to the evaporation of the solvent. The mixture was diluted with water, extracted with DCM (2 times), and the combined organic layers were then washed with a 1 M HCl solution. Aqueous layers were neutralized, extracted with DCM (2 times), and the resulting organic phase was finally washed with brine, dried over $Na_2SO_4$, concentrated under reduced pressure, and purified by chromatography on silica gel (DCM/EtOAc gradient).

## B) Synthesis of Esters

Synthesis of esters via Heck coupling (precursor of **1a-d**, **1m**, **1n**)

In a Schlenk tube equipped with a magnetic stirring bar under nitrogen atmosphere, $Pd(OAc)_2$ (0.02 equiv.), P(*o*-tol)$_3$ (0.04 equiv.), TEA (1.5 equiv.), acrylate (1.3 equiv.), and the aryl halide (1 equiv.) were added in DMF (1 M). The resulting mixture was stirred at 120 °C for 18 h. After complete conversion as monitored by TLC, the mixture was diluted with EtOAc, washed twice with water and a saturated LiCl solution, dried with $Na_2SO_4$, and concentrated under reduced pressure. The crude was purified by chromatography on silica gel (*n*-hexane/EtOAc gradient).

Synthesis of esters via Wittig olefination (precursor of **1e**, **1g-1l**)

To a solution of phosphonium ylide (1.08 equiv.) in MeCN (0.12 M), the desired aldehyde (1 equiv.) was added. The resulting mixture was refluxed 45 min under stirring. After complete conversion of reagents, the mixture was concentrated under reduced pressure and purified by chromatography on silica gel (*n*-hexane/EtOAc gradient).

## C) Synthesis of carboxylic acids

Hydrolysis of esters (precursor of **1a-e**, **1g-n**)

In a round-bottom flask equipped with a stir bar the desired ester (1 equiv.), EtOH (0.15 M) and a 1 M solution of KOH (2.5 equiv.) were added. The resulting mixture was stirred 18 h at 45 °C. After complete conversion as monitored by TLC, the mixture was concentrated under reduced pressure and acidified to pH = 1 with a 1 M HCl solution. During the process, the formation of a precipitate was observed. The solid was filtered, washed with water, and dried under high vacuum to afford the corresponding carboxylic acid.

Synthesis of (E)-3-(furan-2-yl)acrylic-2-d acid (precursor of **1f**)

In a round-bottom flask equipped with a magnetic stirring bar and a condenser a solution of malonic acid (1 equiv.), piperidine (0.35 equiv.) and $D_2O$ (14 equiv.) in pyridine (1.5 M) were refluxed for 2 hours; furfural (1 equiv.) was then added, and the mixture was stirred for additional 3 hours. The resulting mixture was poured in a HCl solution (10% m/V), the precipitate was filtered, washed with water, and dried under vacuo affording the corresponding carboxylic acid (Yield = 74%; deuterium incorporation: 80%).

## D) Synthesis of amides

Synthesis of amides via coupling with acyl chlorides (precursor of **1a-c**, **1e-n**)

In a 25 mL round-bottom flask equipped with a magnetic stirring bar, the desired acid was dissolved in DCM (0.6 M), and a catalytic amount of DMF (3 drops) was added. The solution was then cooled to 0 °C, and oxalyl chloride (1.5 equiv) was added. The solution was stirred for 1 h at room temperature. Then, the mixture was concentrated under reduced pressure to afford the acyl chloride, which was added to a solution of DMAP (0.02 equiv.), TEA (1 equiv.), and the desired amine (1 equiv.) in DCM (0.25 M) at 0 °C. The mixture was stirred for 18 h at room temperature. After complete conversion as monitored by TLC, the solution was diluted with DCM and washed with saturated $NH_4Cl$ solution, followed by a saturated $NaHCO_3$ one. The aqueous layers were extracted with DCM (3 times), and the combined organic phase was finally washed with brine, dried over $Na_2SO_4$, and concentrated under reduced pressure. The crude was finally purified by chromatography on silica gel (*n*-hexane/EtOAc gradient) to afford the desired products.

Synthesis of amides via nucleophilic substitution (precursor of **1d**)

To a solution of primary amide (1 equiv.) in DMF (0.6 M) at 0° C, NaH (60% in paraffine oil, 1.3 equiv.) was slowly added, and the mixture was stirred for 1 h. The desired alkyl halide (1.5 equiv.) was then slowly added, and the reaction was stirred at room temperature for 18 h. After complete conversion as monitored by TLC, the mixture was quenched with a saturated $NH_4Cl$ solution and extracted with EtOAc (3 times). The combined organic layers were washed with brine (3 times), dried over $Na_2SO_4$, and concentrated under reduced pressure. The crude was purified by chromatography on silica gel (*n*-hexane/EtOAc gradient) to afford the desired product (Yield = 53%).

## E) Synthesis of precursors of 1o and 1p

Synthesis of **1-benzyl-1H-pyrrole-2-carbaldehyde**

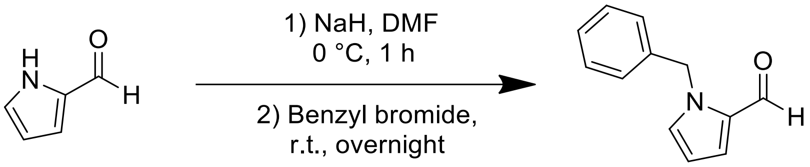


To a solution of pyrrole-2-carboxaldehyde (1 equiv.) in DMF (0.5 M) at 0° C, NaH (60% in paraffine oil, 1.5 equiv.) was slowly added and the mixture was stirred for 1 h. Benzyl bromide (1.2 equiv.) was then slowly added, and the reaction was stirred at room temperature overnight. After complete conversion as monitored by TLC, the mixture was quenched with a saturated $NH_4Cl$ solution and extracted with EtOAc (3 times). The combined organic layers were washed with brine (3 times), dried over $Na_2SO_4$, and concentrated under reduced pressure. The crude was purified by chromatography on silica gel (*n*-hexane/EtOAc 85:15) to afford 1-benzyl-1H-pyrrole-2-carbaldehyde. (Yield 73%)

Synthesis of **(E)-ethyl 3-(1-benzyl-1H-pyrrol-2-yl)acrylate**

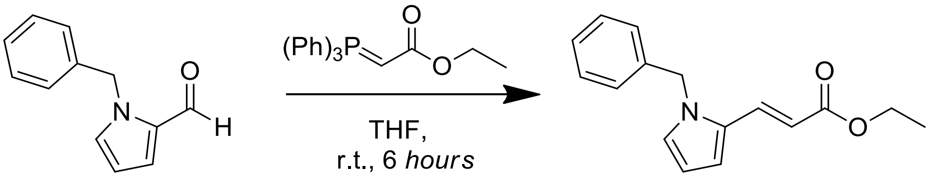


To a solution of phosphonium ylide (1.5 equiv.) in THF (0.5 M), the 1-benzyl-1H-pyrrole-2-carbaldehyde (1 equiv.) was added. The resulting mixture was refluxed 6 hours under stirring. After that, the mixture was quenched with a saturated $NH_4Cl$ solution and extracted with EtOAc (3 times). The combined organic layers were washed with brine (3 times), dried over $Na_2SO_4$, and concentrated under reduced pressure. The crude was purified by chromatography on silica gel (*n*-hexane/MTBE 88:12) to afford (E)-ethyl 3-(1-benzyl-1H-pyrrol-2-yl)acrylate. (Yield 59%)

Synthesis of **(E)-3-(1-benzyl-1H-pyrrol-2-yl)acrylic acid**

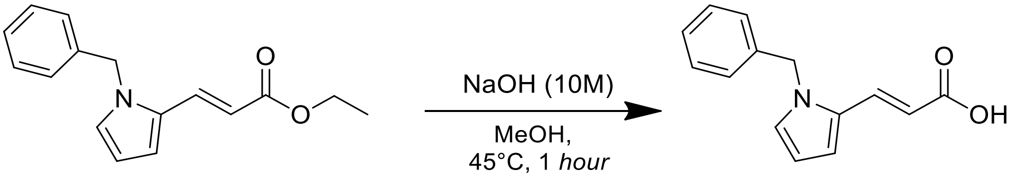


In a round-bottom flask equipped with a stir bar, (E)-ethyl 3-(1-benzyl-1H-pyrrol-2-yl)acrylate, MeOH (0.6M) and a 10M solution of NaOH (2 equiv.) were added. The resulting mixture was stirred for 1 hour at 45°C. After complete conversion as monitored by TLC, the mixture was concentrated under reduced pressure and acidified to pH=1 with a 1M HCl solution. During the process, the formation of a precipitate was observed. The following solid was filtered, washed with water, and finally dried under high vacuum to afford (E)-3-(1-benzyl-1H-pyrrol-2-yl)acrylic acid. (Yield 56%)

Synthesis of **(E)-N-benzyl-3-(1-benzyl-1H-pyrrol-2-yl)-N-(prop-2-yn-1-yl)acrylamide** (precursor of **1o**)

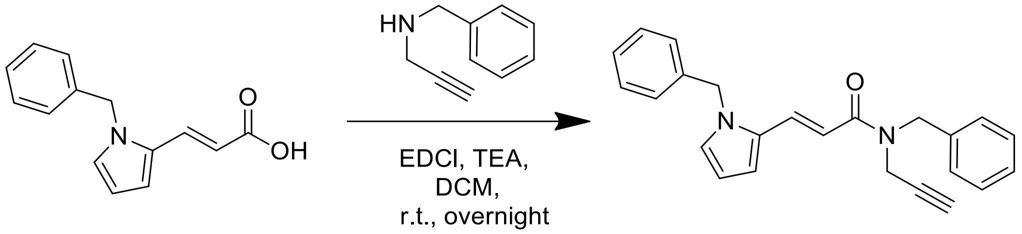


In a round-bottom flask, N-benzylprop-2-yn-1-amine (1 equiv.), 1-Ethyl-3-(3-dimethylaminopropyl)carbodiimide (EDCl) (1.3 equiv.) and $Et_3N$ (1.3 equiv.) were added in DCM (0.1 M). The mixture was stirred at room temperature for 15 minutes; then, a solution of (E)-3-(1-benzyl-1H-pyrrol-2-yl)acrylic acid (1 equiv.) and DCM was slowly added at 0°C, and the mixture was reacted overnight. After complete conversion as monitored by TLC, the solution was quenched with a saturated $NH_4Cl$ solution and extracted with EtOAc (3 times). The combined organic layers were washed with brine (3 times), dried over $Na_2SO_4$, and concentrated under reduced pressure. The crude was purified by chromatography on silica gel (*n*-hexane/EtOAc 7:3) to afford (E)-N-benzyl-3-(1-benzyl-1H-pyrrol-2-yl)-N-(prop-2-yn-1-yl)acrylamide. (Yield 47%)

Synthesis of **1-methyl-1H-indole-2-carbaldehyde**

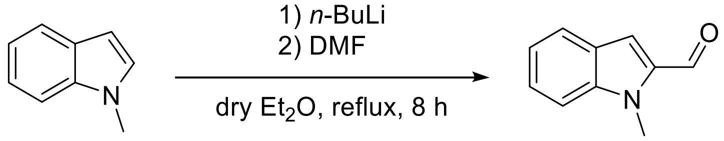


To a solution of 1-methyl-1H-indole (1 equiv.) in $Et_2O$ (0.7 M) at 0° C, *n*-Buli (2.5 M in hexane, 1.1 equiv.) was slowly added, and the mixture was refluxed for 3 hours. The reaction was cooled down to room temperature, then DMF (1.5 equiv.) was added, and the solution was refluxed for 5 h. After complete conversion as monitored by TLC, the mixture was quenched with a saturated $NH_4Cl$ solution and extracted with EtOAc (3 times). The combined organic layers were dried over $Na_2SO_4$ and concentrated under reduced pressure. The crude was purified by chromatography on silica gel (*n*-hexane/EtOAc gradient) to afford the desired product (Yield = 63%).

Synthesis of **(E)-ethyl 3-(1-methyl-1H-indol-2-yl)acrylate**

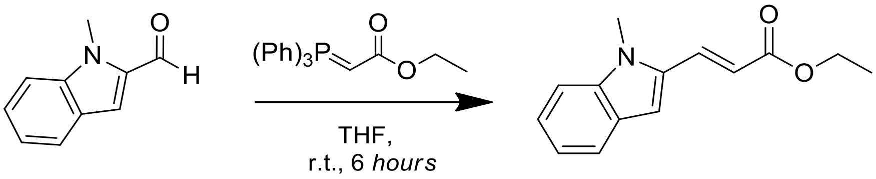


To a solution of phosphonium ylide (1.5 equiv.) in THF (0.5 M), the 1-benzyl-1H-pyrrole-2-carbaldehyde (1 equiv.) was added. The resulting mixture was refluxed 6 hours under stirring. After that, the mixture was quenched with a saturated $NH_4Cl$ solution and extracted with EtOAc (3 times). The combined organic layers were washed with brine (3 times), dried over $Na_2SO_4$, and concentrated under reduced pressure. The crude was purified by chromatography on silica gel (*n*-hexane/EtOAc 85:15) to afford (E)-ethyl 3-(1-methyl-1H-indol-2-yl)acrylate. (Yield 81%)

Synthesis of **(E)-3-(1-methyl-1H-indol-2-yl)acrylic acid**

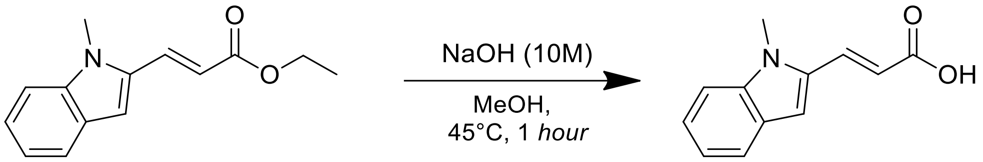


In a round-bottom flask equipped with a stir bar, €-ethyl 3-(1-methyl-1H-indol-2-yl)acrylate, MeOH (0.6M) and a 10M solution of NaOH (2 equiv.) were added. The resulting mixture was stirred for 1 hour at 45°C. After complete conversion as monitored by TLC, the mixture was concentrated under reduced pressure and acidified to pH=1 with a 1M HCl solution. During the process, the formation of a precipitate was observed. The following solid was filtered, washed with water, and finally dried under high vacuum to afford (E)-3-(1-methyl-1H-indol-2-yl)acrylic acid. (Yield 89%)

Synthesis of **(E)-N-benzyl-3-(1-methyl-1H-indol-2-yl)-N-(prop-2-yn-1-yl)acrylamide** (precursor of **1p**)

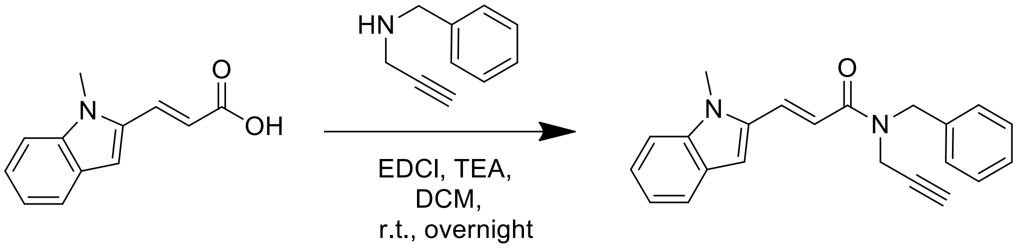


In a round-bottom flask, N-benzylprop-2-yn-1-amine (1 equiv.), 1-Ethyl-3-(3-dimethylaminopropyl)carbodiimide (EDCl) (1.3 equiv.) and $Et_3N$ (1.3 equiv.) in DCM (0.1 M) were added. The mixture was stirred at room temperature for 15 minutes, then a solution of (E)-3-(1-benzyl-1H-pyrrol-2-yl)acrylic acid (1 equiv.) and DCM was slowly added at 0°C, and the mixture was reacted overnight. After complete conversion as monitored by TLC, the solution was quenched with a saturated $NH_4Cl$ solution and extracted with EtOAc (3 times). The combined organic layers were washed with brine (3 times), dried over $Na_2SO_4$, and concentrated under reduced pressure. The crude was purified by chromatography on silica gel (*n*-hexane/EtOAc 72:28) to afford (E)-N-benzyl-3-(1-methyl-1H-indol-2-yl)-N-(prop-2-yn-1-yl)acrylamide. (Yield = 58%)

# Copies of NMR spectra

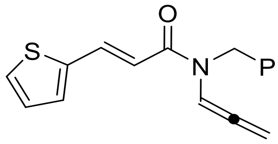

**1a**

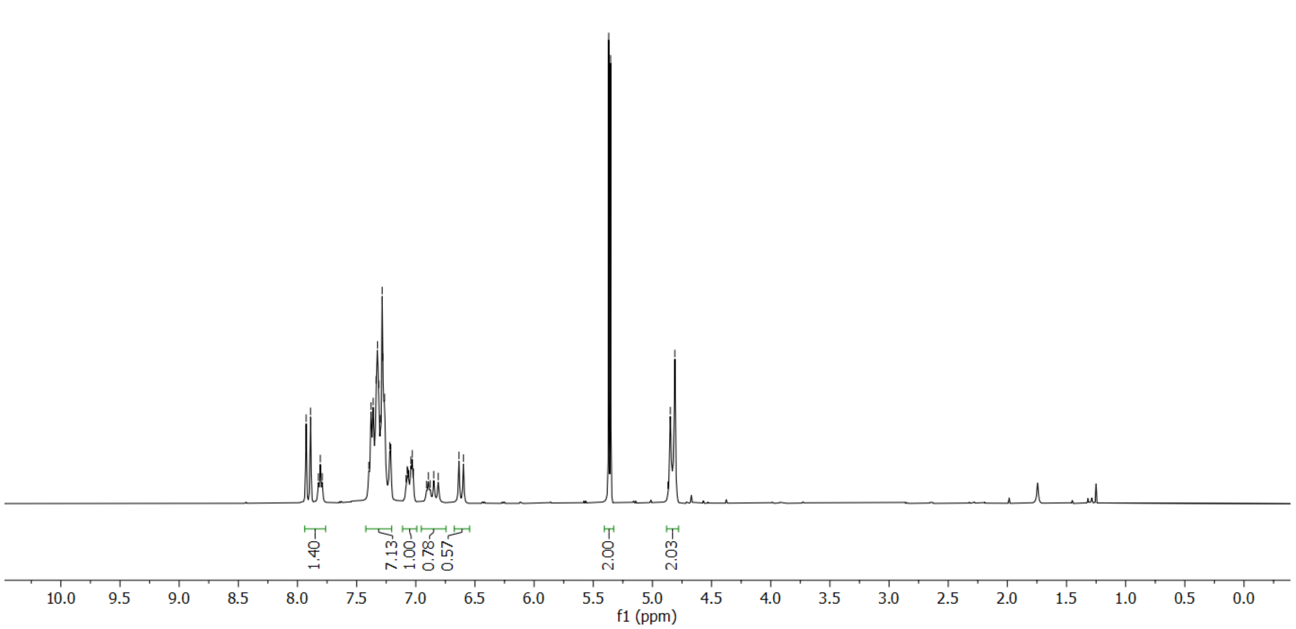


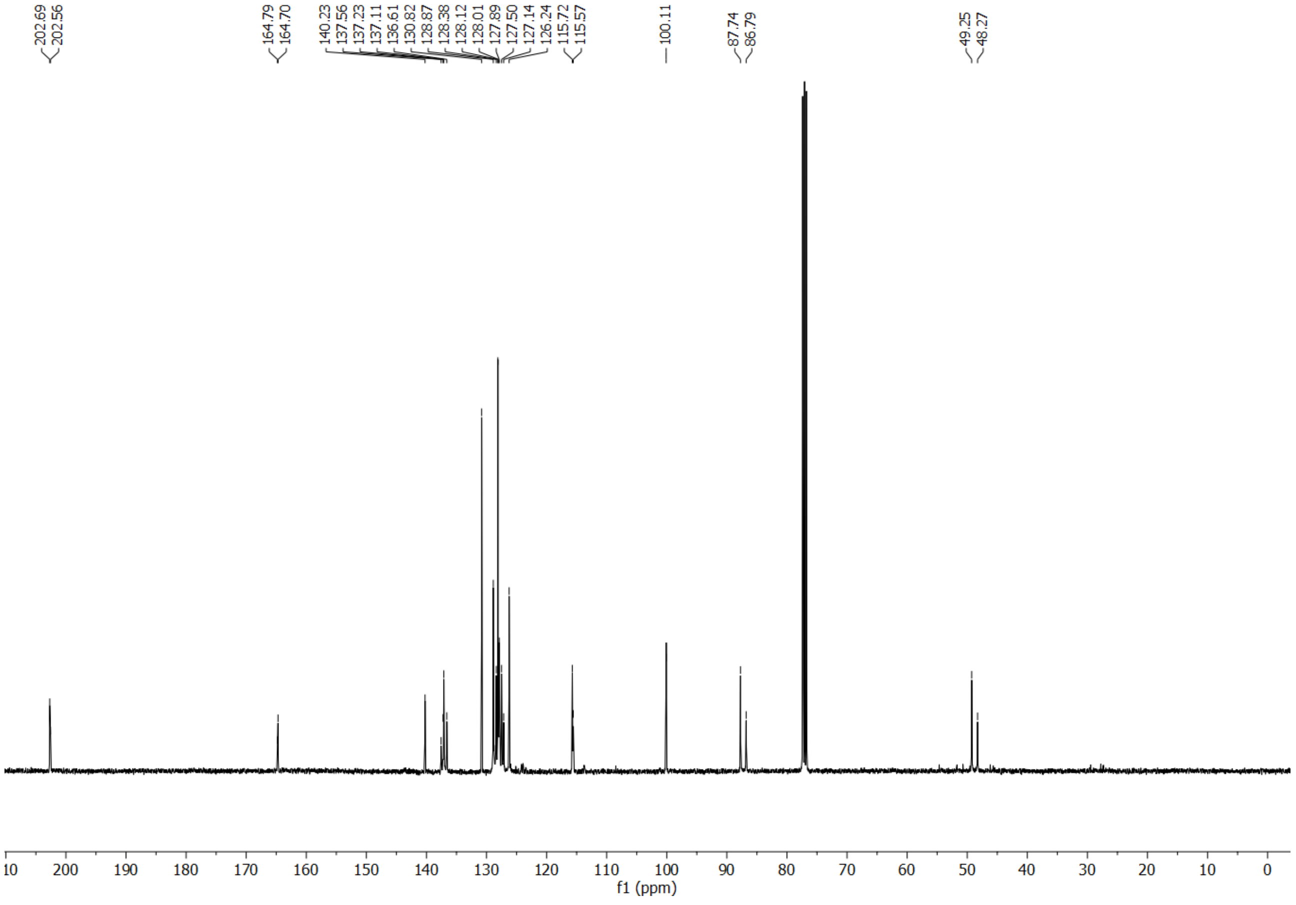

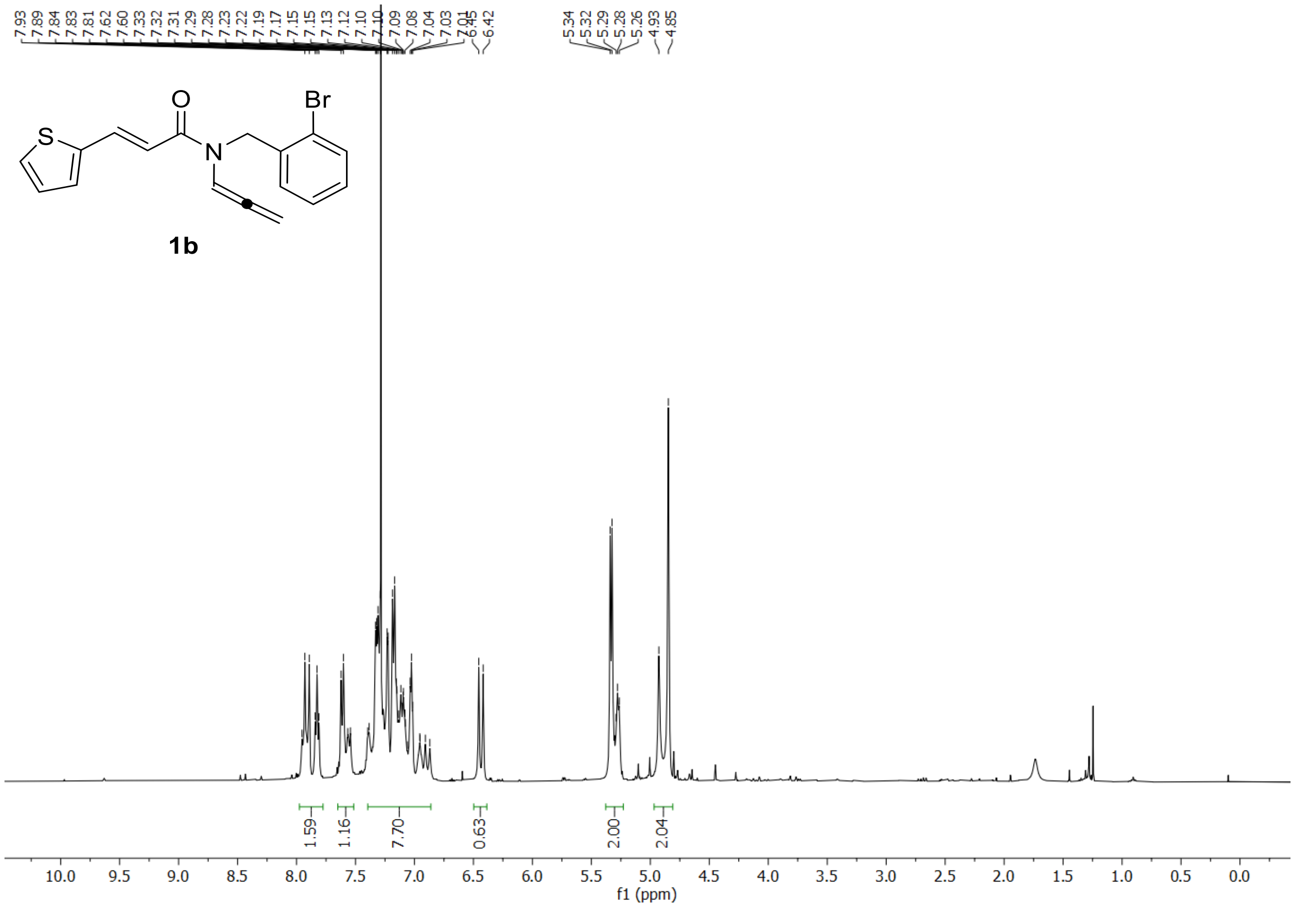

O
Br
S
N
1b
7.93
7.89
7.84
7.83
7.81
7.62
7.60
7.33
7.32
7.31
7.29
7.28
7.23
7.22
7.19
7.17
7.15
7.15
7.13
7.12
7.10
7.10
7.09
7.08
7.04
7.03
7.01
6.45
6.42
5.34
5.32
5.29
5.28
5.26
4.93
4.85
1.59
1.16
7.70
0.63
2.00
2.04
f1 (ppm)


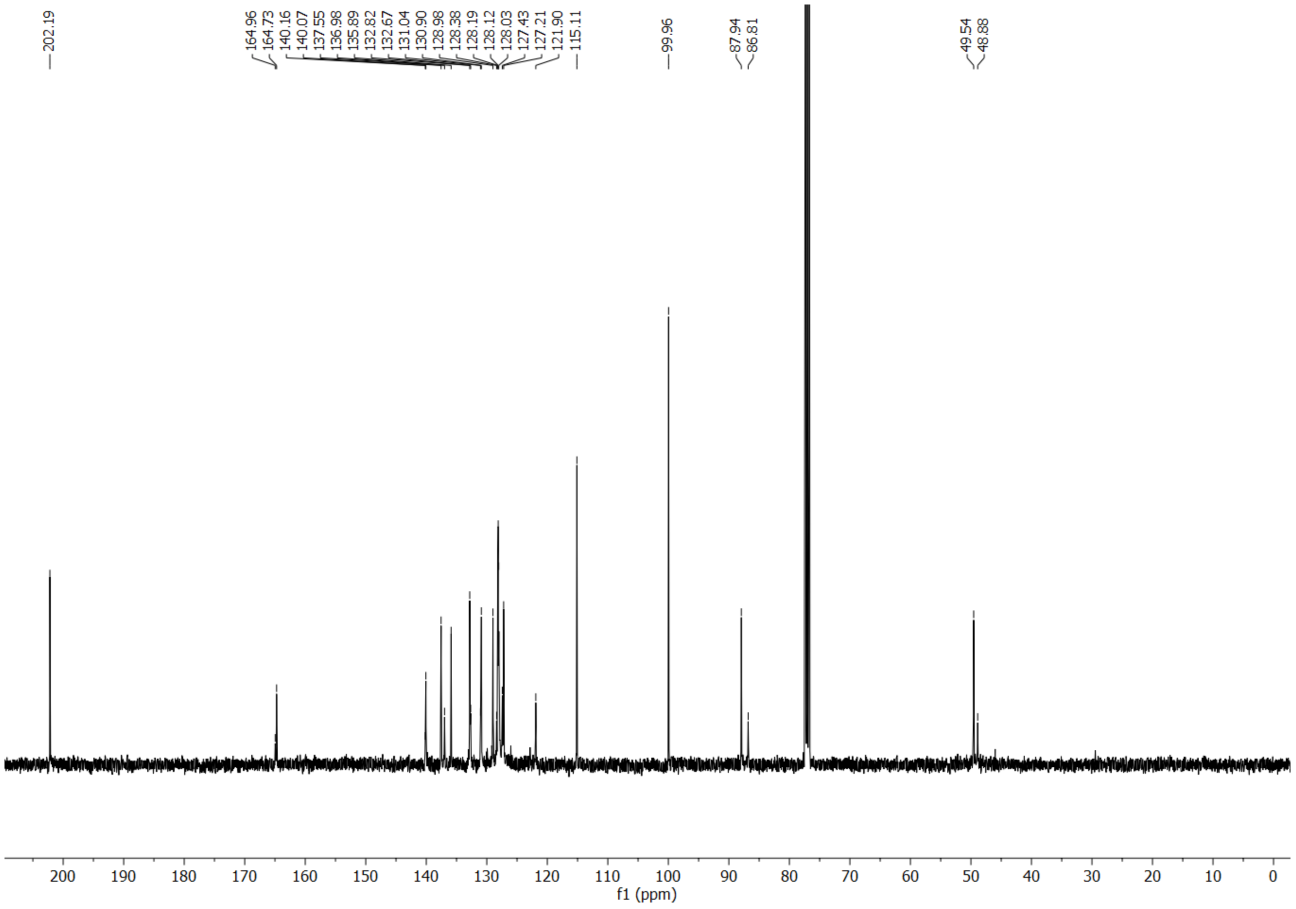

202.19
164.96
164.73
140.16
140.07
137.55
136.98
135.89
132.82
132.67
131.04
130.90
128.98
128.38
128.19
128.12
128.03
127.43
127.21
121.90
115.11
99.96
87.94
86.81
49.54
48.88
f1 (ppm)

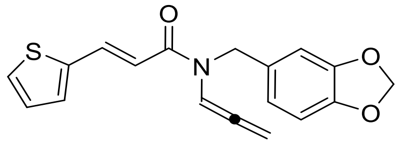
S
O
N
O
O

**1c**

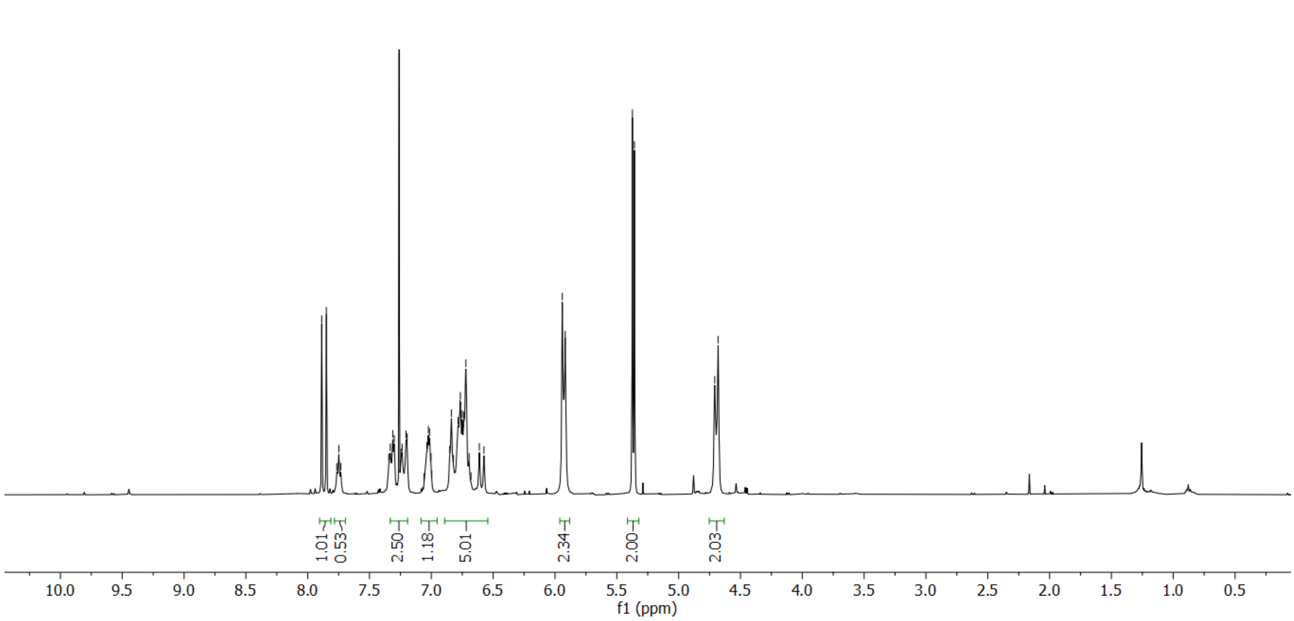
7.89
7.85
7.77
7.75
7.73
7.34
7.33
7.31
7.30
7.25
7.24
7.20
7.20
7.06
7.04
7.02
7.01
7.00
7.00
6.85
6.84
6.82
6.78
6.77
6.77
6.75
6.74
6.74
6.72
6.69
6.68
6.61
6.57
5.94
5.92
5.37
5.36
4.71
4.68
1.01
0.53
2.50
1.18
5.01
2.34
2.00
2.03
f1 (ppm)

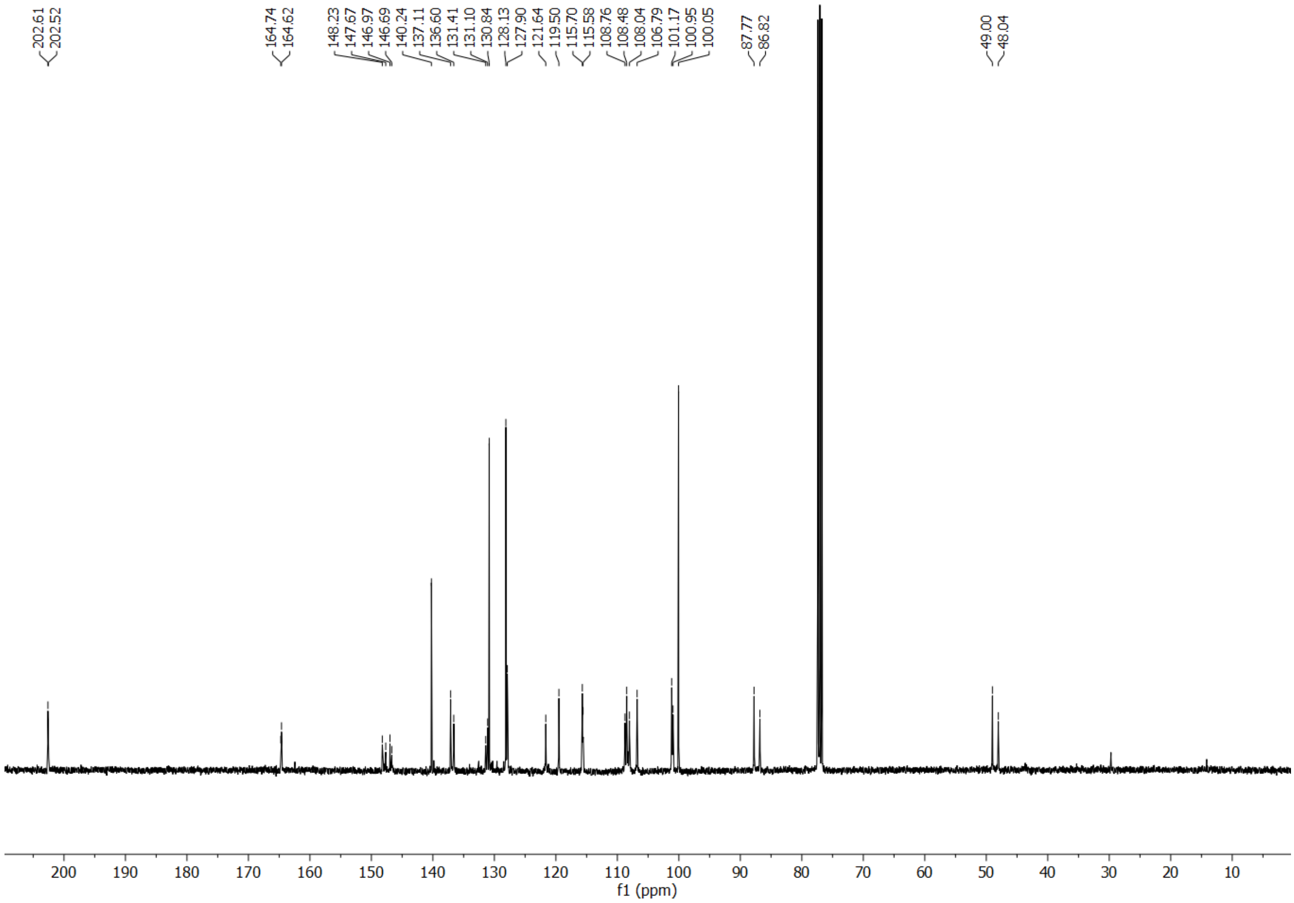
202.61
202.52
164.74
164.62
148.23
147.67
146.97
146.69
140.24
137.11
136.60
131.41
131.10
130.84
128.13
127.90
121.64
119.50
115.70
115.58
108.76
108.48
108.04
106.79
101.17
100.95
100.05
87.77
86.82
49.00
48.04
f1 (ppm)

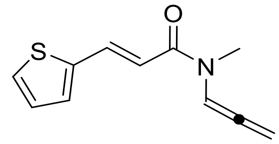

**1d**

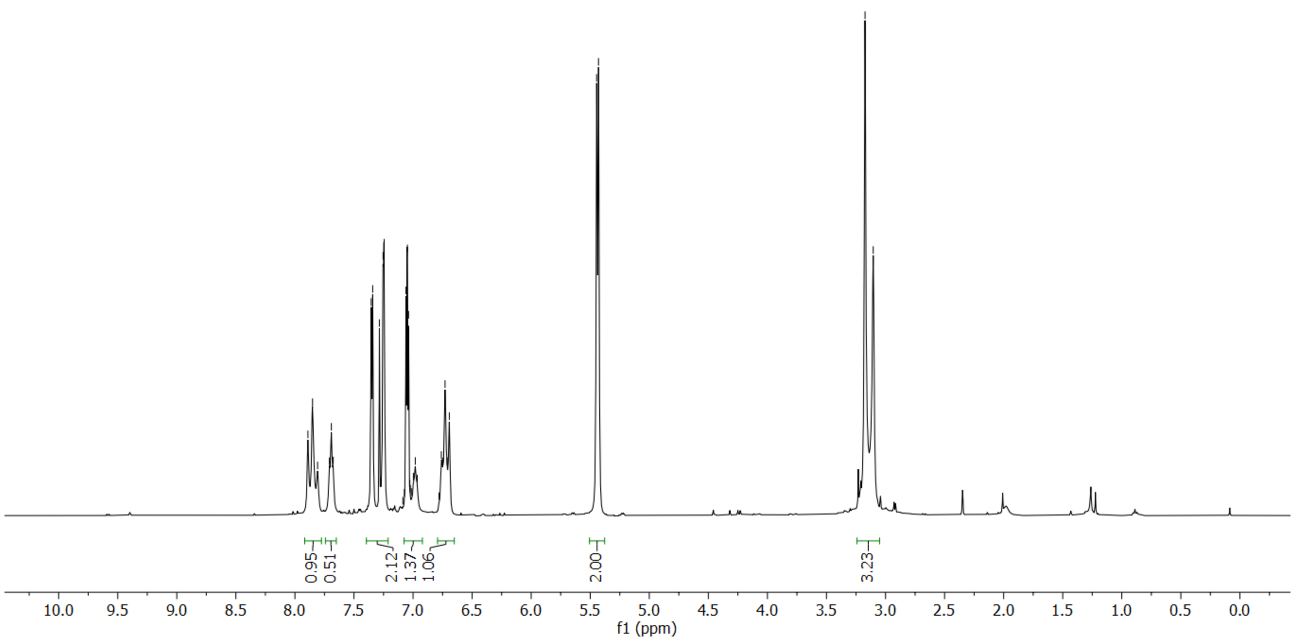
7.89 7.85 7.81 7.71 7.69 7.68 7.35 7.34 7.28 7.27 7.25 7.24 7.09 7.07 7.06 7.05 7.05 7.04 7.03 7.02 7.01 7.00 6.98 6.97 6.78 6.76 6.75 6.73 6.71 6.69 5.45 5.43
3.17 3.10
0.95 0.51 2.12 1.37 1.06 2.00 3.23
10.0 9.5 9.0 8.5 8.0 7.5 7.0 6.5 6.0 5.5 5.0 4.5 4.0 3.5 3.0 2.5 2.0 1.5 1.0 0.5 0.0
f1 (ppm)

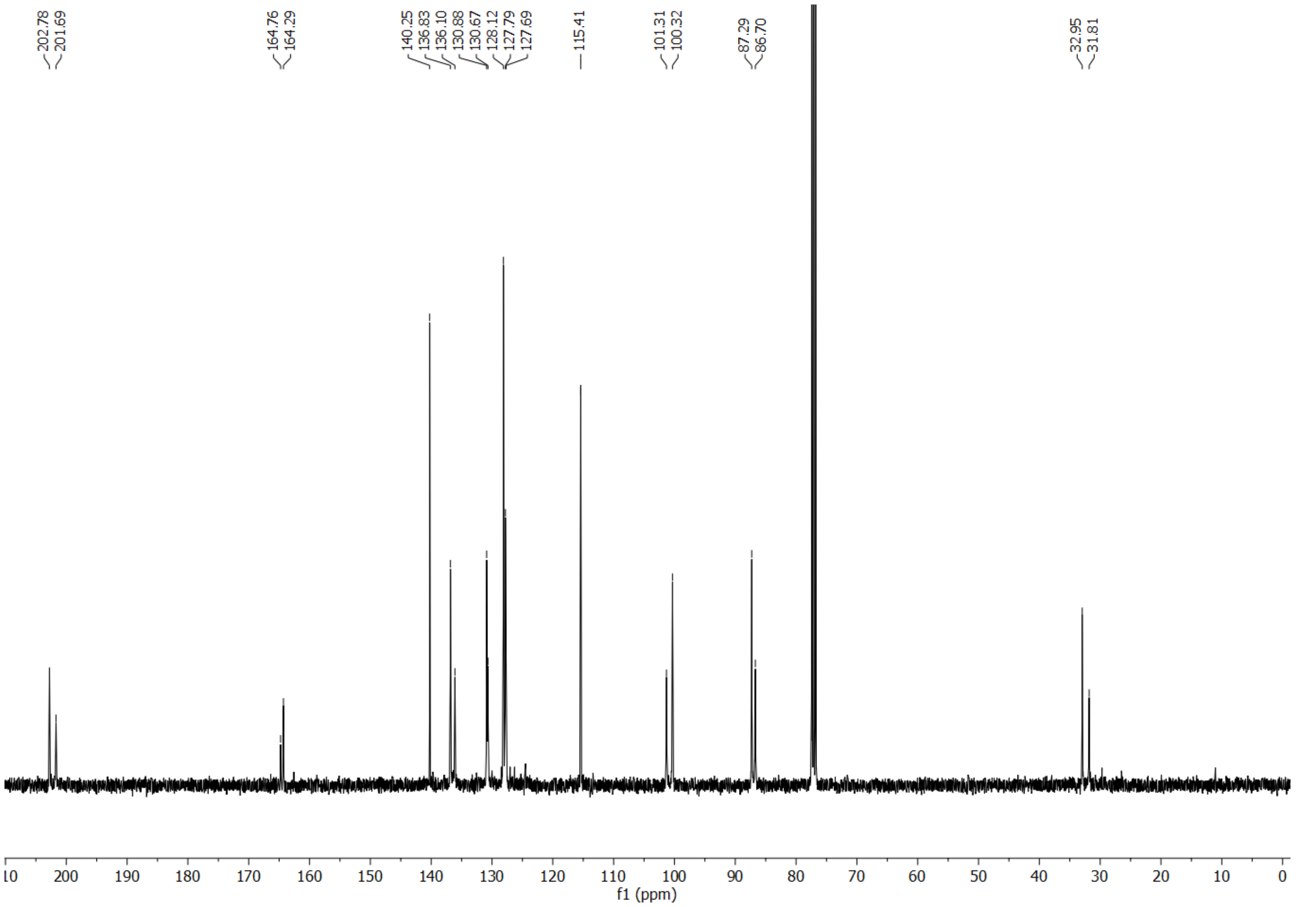
202.78 201.69
164.76 164.29
140.25 136.83 136.10 130.88 130.67 128.12 127.79 127.69
115.41
101.31 100.32
87.29 86.70
32.95 31.81
10 200 190 180 170 160 150 140 130 120 110 100 90 80 70 60 50 40 30 20 10 0
f1 (ppm)

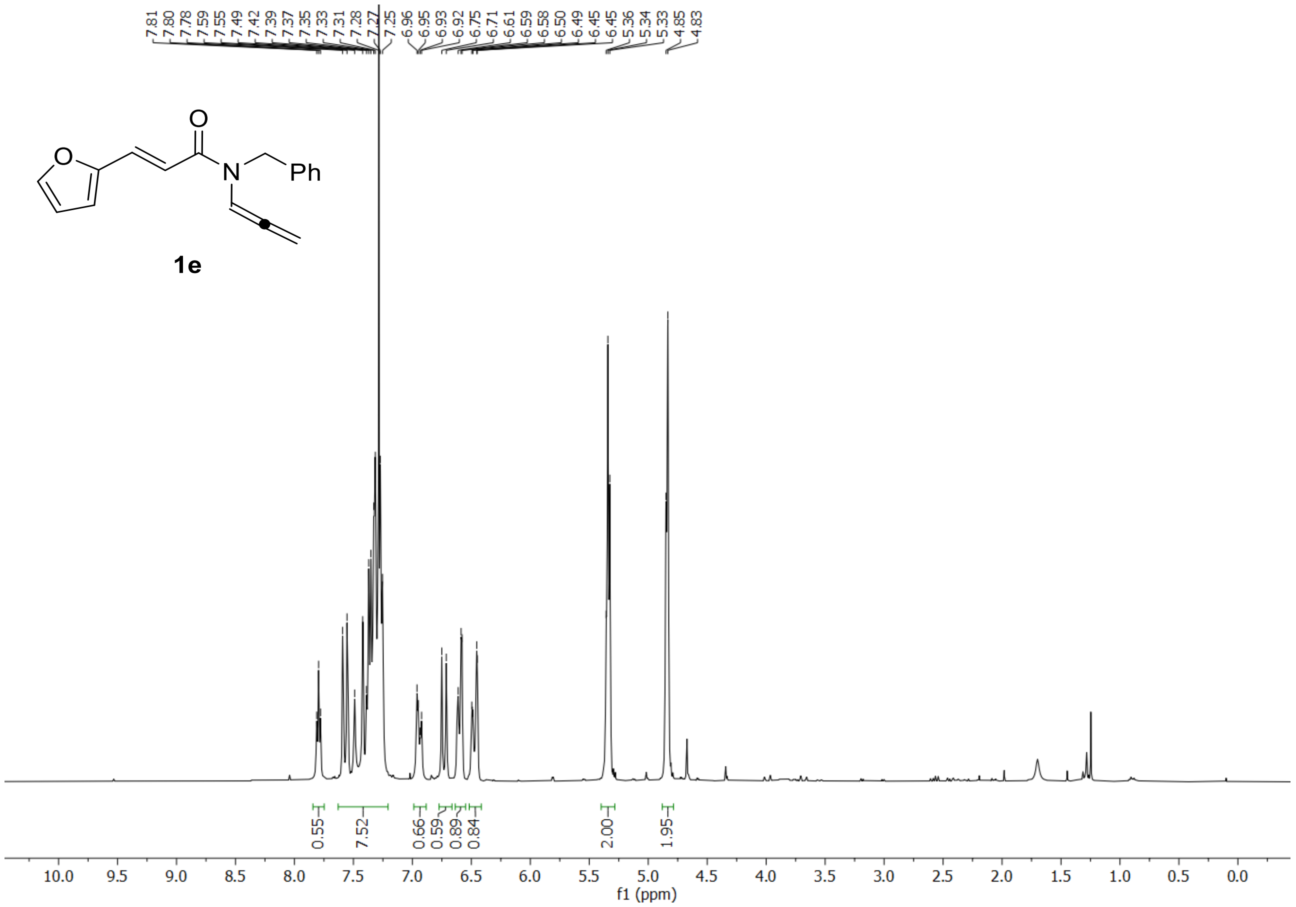

1e
f1 (ppm)


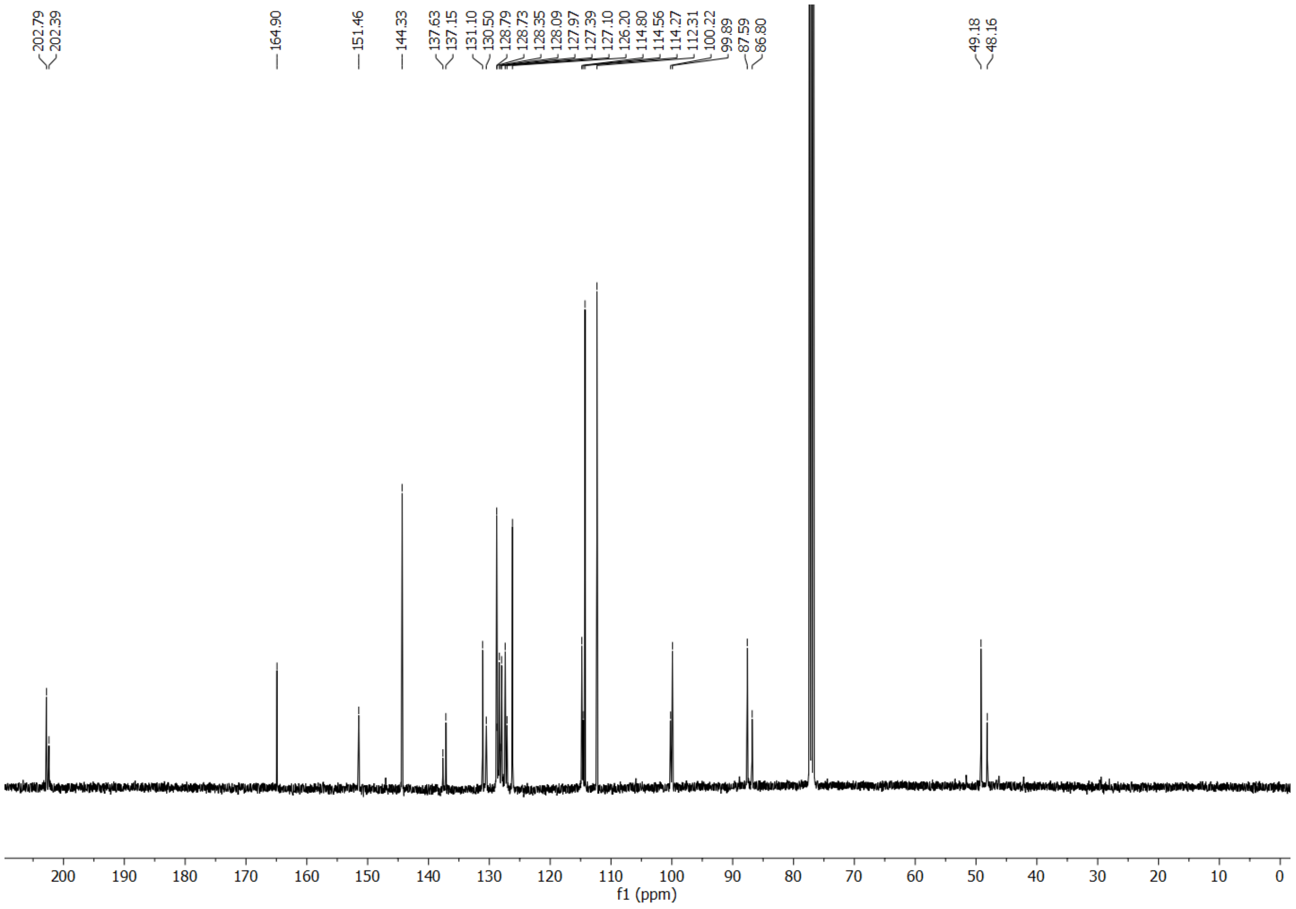

f1 (ppm)

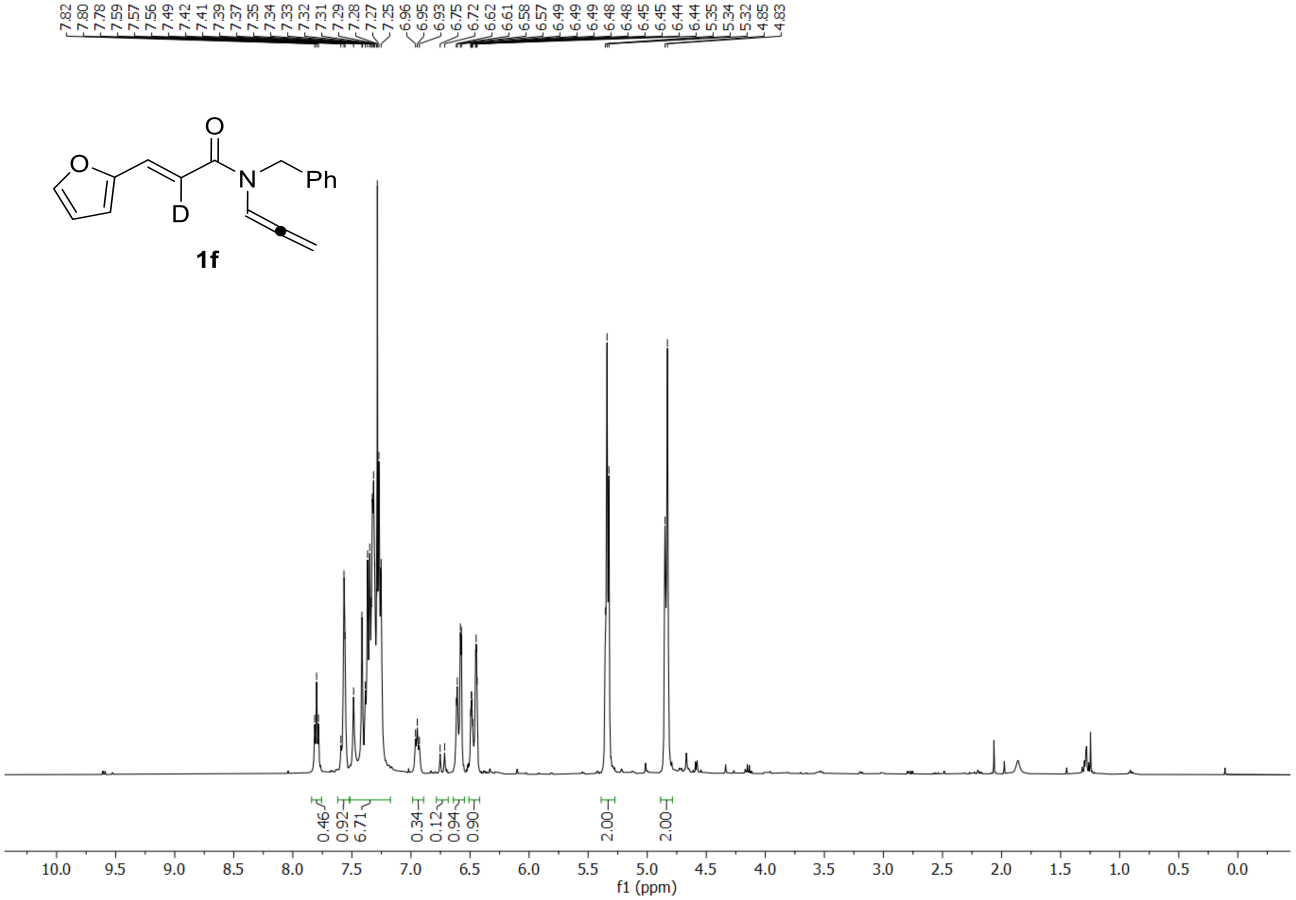
O
N
Ph
D
7.82
7.80
7.78
7.59
7.57
7.56
7.49
7.42
7.41
7.39
7.37
7.35
7.34
7.33
7.32
7.31
7.29
7.28
7.27
7.25
6.96
6.95
6.93
6.75
6.72
6.62
6.61
6.58
6.57
6.49
6.49
6.49
6.48
6.48
6.45
6.45
6.44
6.44
5.35
5.34
5.32
4.85
4.83
0.46
0.92
6.71
0.34
0.12
0.94
0.90
2.00
2.00
10.0 9.5 9.0 8.5 8.0 7.5 7.0 6.5 6.0 5.5 5.0 4.5 4.0 3.5 3.0 2.5 2.0 1.5 1.0 0.5 0.0
f1 (ppm)

**1f**

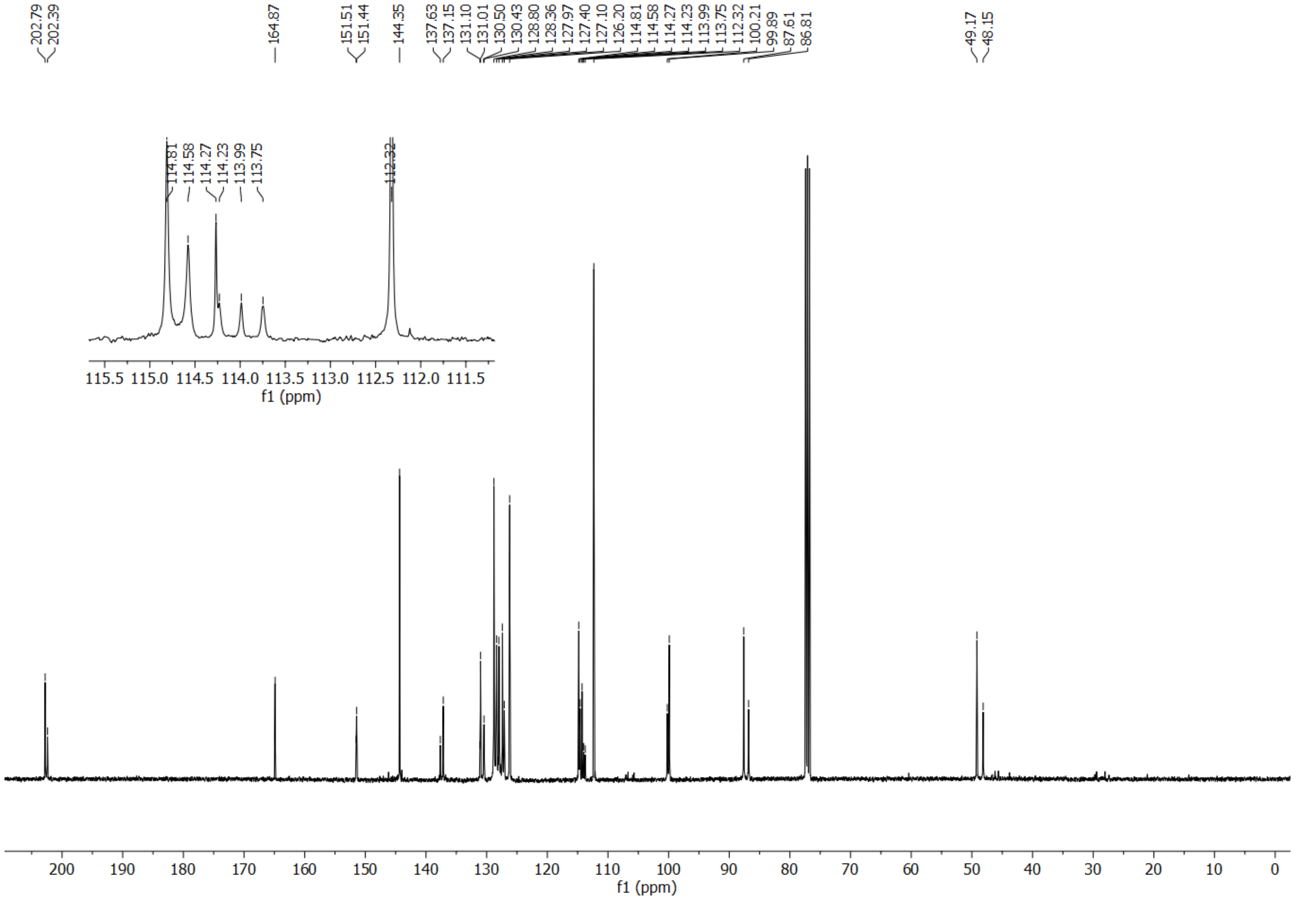
202.79
202.39
164.87
151.51
151.44
144.35
137.63
137.15
131.10
131.01
130.50
130.43
128.80
128.36
127.97
127.40
127.10
126.20
114.81
114.58
114.27
114.23
113.99
113.75
112.32
100.21
99.89
87.61
86.81
49.17
48.15
114.81
114.58
114.27
114.23
113.99
113.75
112.32
115.5 115.0 114.5 114.0 113.5 113.0 112.5 112.0 111.5
f1 (ppm)
200 190 180 170 160 150 140 130 120 110 100 90 80 70 60 50 40 30 20 10 0
f1 (ppm)

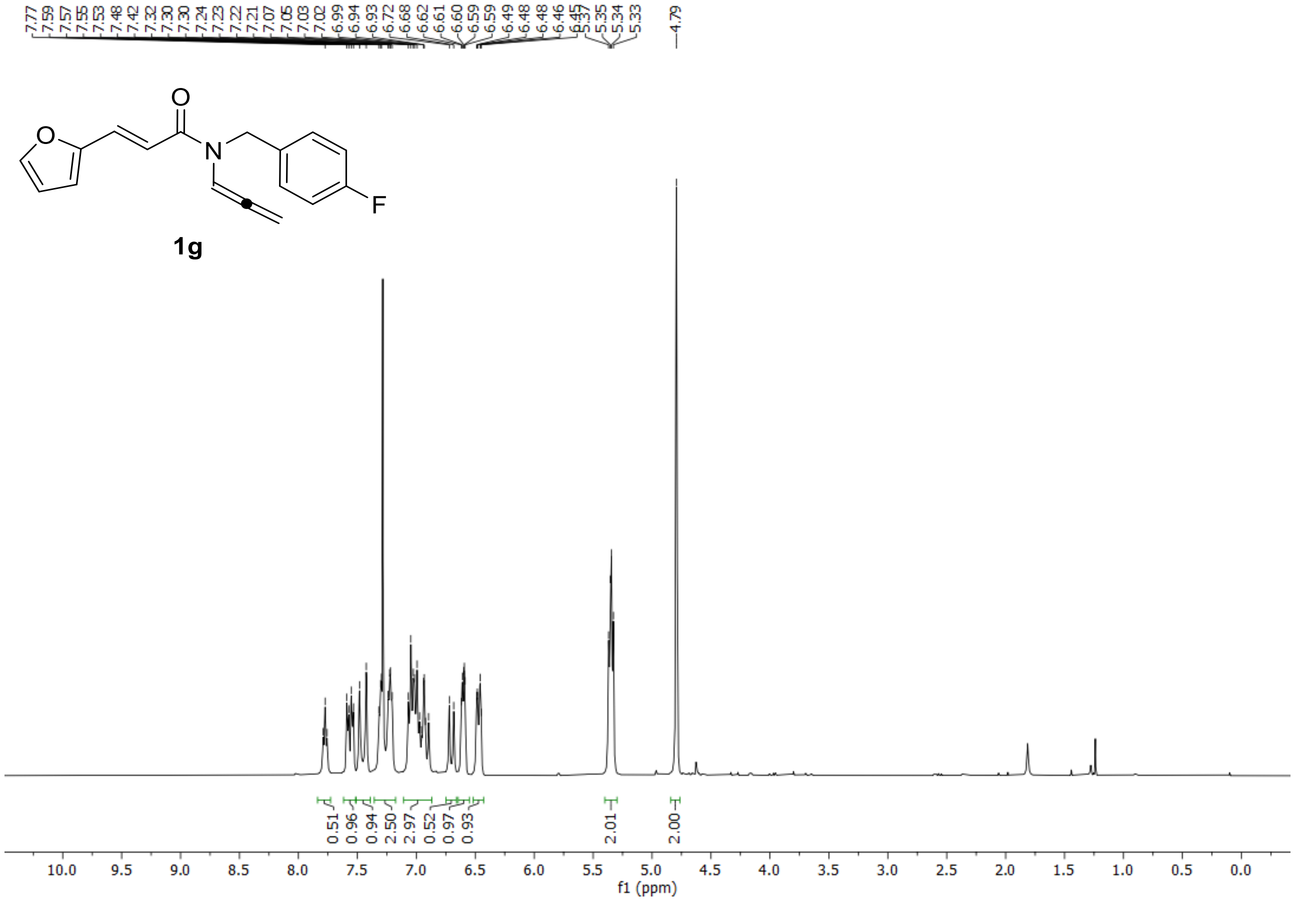

**1g**

7.77
7.59
7.57
7.55
7.53
7.48
7.42
7.32
7.30
7.30
7.24
7.23
7.22
7.21
7.07
7.05
7.03
7.02
6.99
6.94
6.93
6.72
6.68
6.62
6.61
6.60
6.59
6.59
6.49
6.48
6.48
6.46
6.45
5.37
5.35
5.34
5.33
4.79
0.51
0.96
0.94
2.50
2.97
0.52
0.97
0.93
2.01
2.00
10.0 9.5 9.0 8.5 8.0 7.5 7.0 6.5 6.0 5.5 5.0 4.5 4.0 3.5 3.0 2.5 2.0 1.5 1.0 0.5 0.0
f1 (ppm)

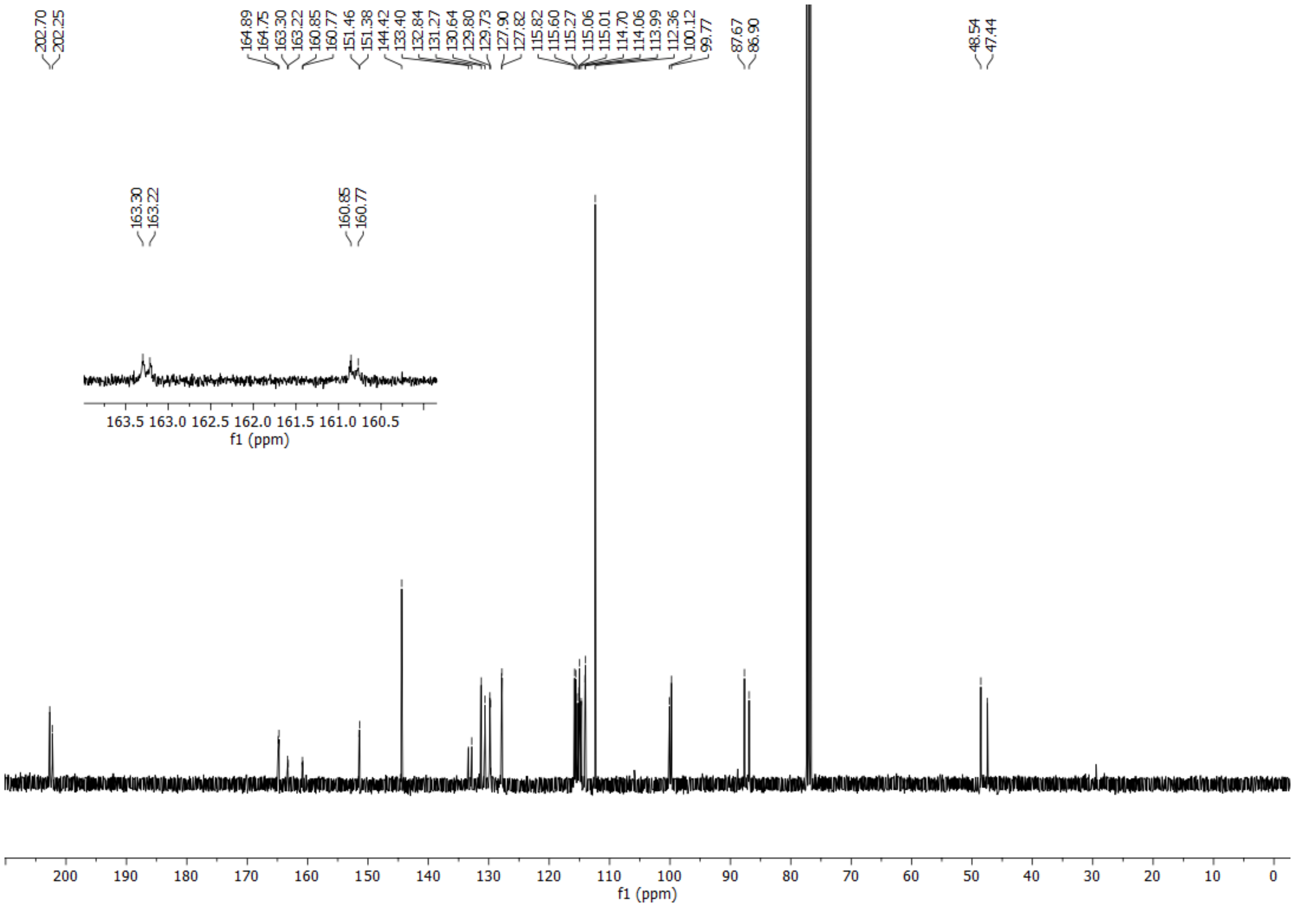
202.70
202.25
164.89
164.75
163.30
163.22
160.85
160.77
151.46
151.38
144.42
133.40
132.84
131.27
130.64
129.80
129.73
127.90
127.82
115.82
115.60
115.27
115.06
115.01
114.70
114.06
113.99
112.36
100.12
99.77
87.67
86.90
48.54
47.44
163.30
163.22
160.85
160.77
163.5 163.0 162.5 162.0 161.5 161.0 160.5
f1 (ppm)
200 190 180 170 160 150 140 130 120 110 100 90 80 70 60 50 40 30 20 10 0
f1 (ppm)

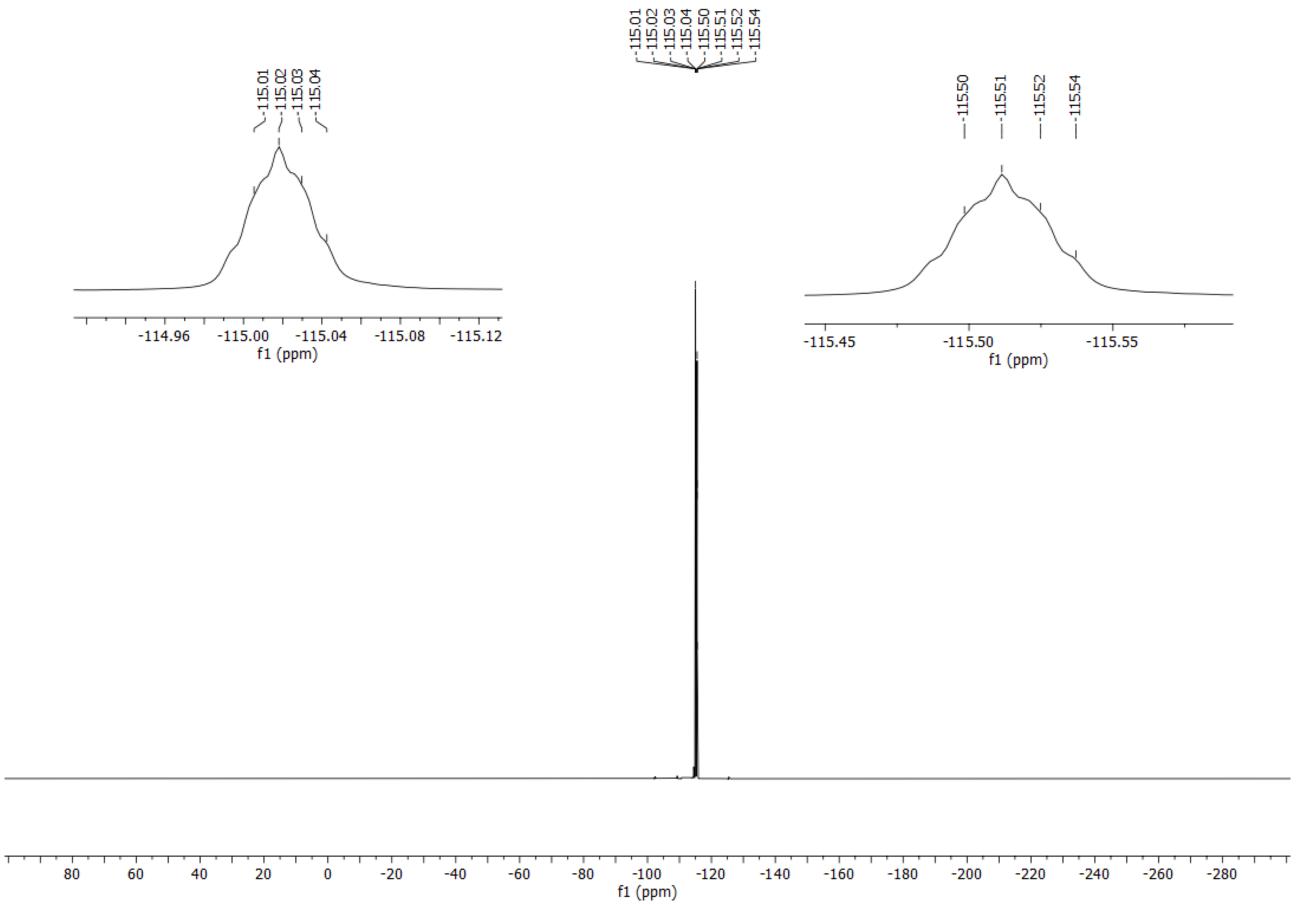

-115.01
-115.02
-115.03
-115.04
-115.50
-115.51
-115.52
-115.54
-114.96 -115.00 -115.04 -115.08 -115.12
f1 (ppm)
-115.45 -115.50 -115.55
f1 (ppm)
80 60 40 20 0 -20 -40 -60 -80 -100 -120 -140 -160 -180 -200 -220 -240 -260 -280
f1 (ppm)

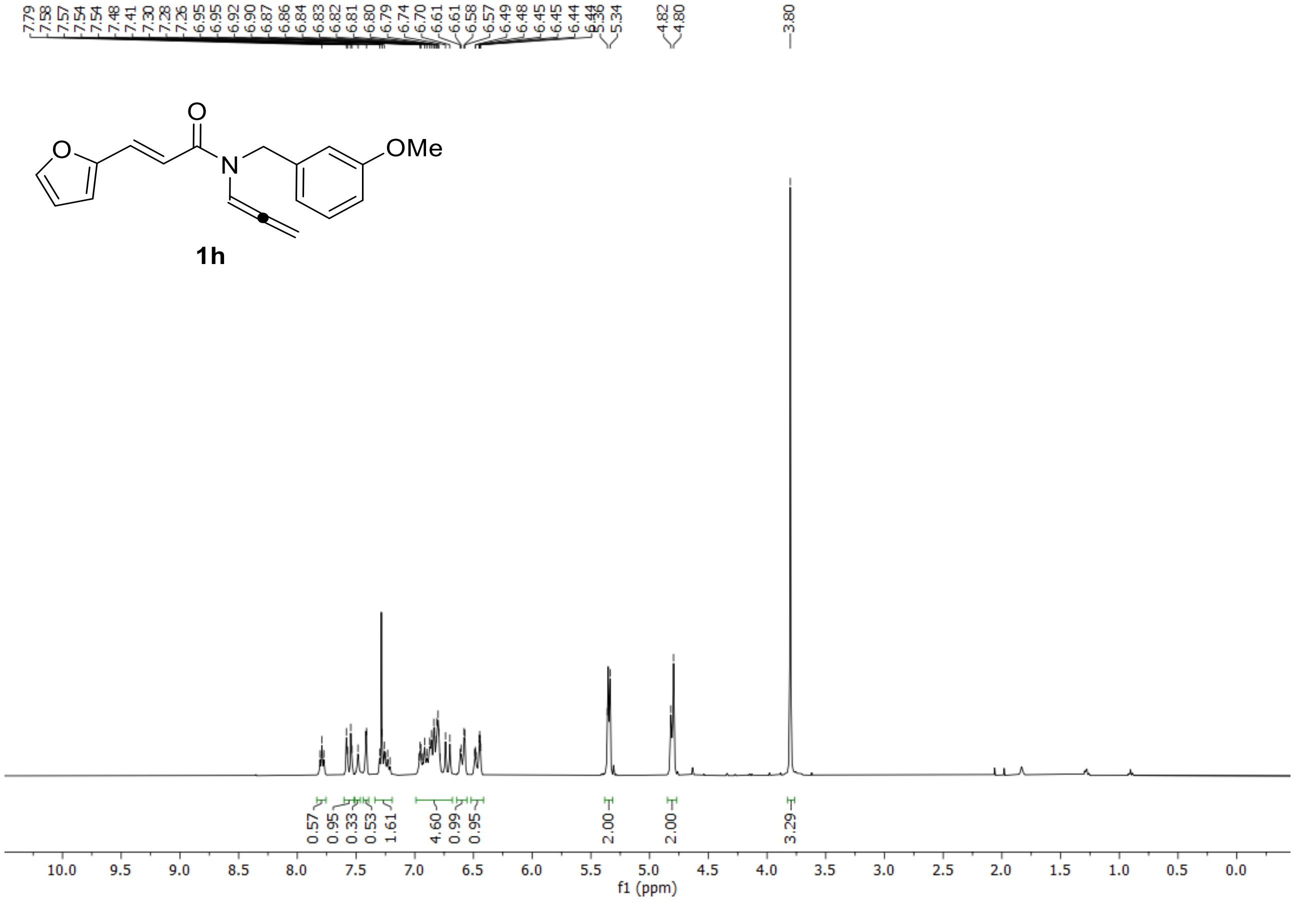
7.79
7.58
7.57
7.54
7.54
7.48
7.41
7.30
7.28
7.26
6.95
6.95
6.92
6.90
6.87
6.86
6.84
6.83
6.82
6.81
6.80
6.79
6.74
6.70
6.61
6.61
6.58
6.57
6.49
6.48
6.45
6.45
6.44
6.44
5.36
5.34
4.82
4.80
3.80
O
O
N
OMe
1h
0.57
0.95
0.33
0.53
1.61
4.60
0.99
0.95
2.00
2.00
3.29
10.0 9.5 9.0 8.5 8.0 7.5 7.0 6.5 6.0 5.5 5.0 4.5 4.0 3.5 3.0 2.5 2.0 1.5 1.0 0.5 0.0
f1 (ppm)

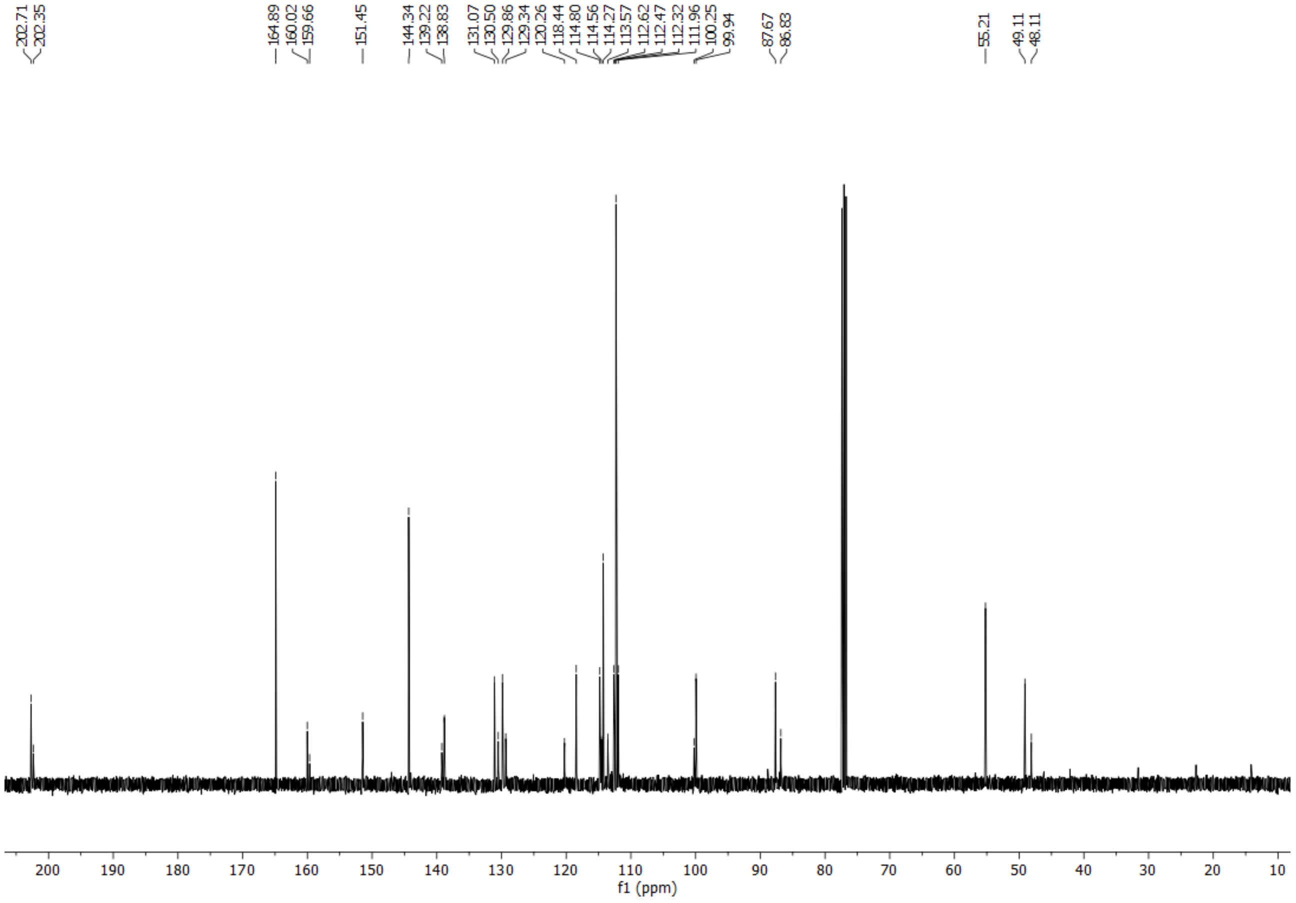
202.71
202.35
164.89
160.02
159.66
151.45
144.34
139.22
138.83
131.07
130.50
129.86
129.34
120.26
118.44
114.80
114.56
114.27
113.57
112.62
112.47
112.32
111.96
100.25
99.94
87.67
86.83
55.21
49.11
48.11
200 190 180 170 160 150 140 130 120 110 100 90 80 70 60 50 40 30 20 10
f1 (ppm)

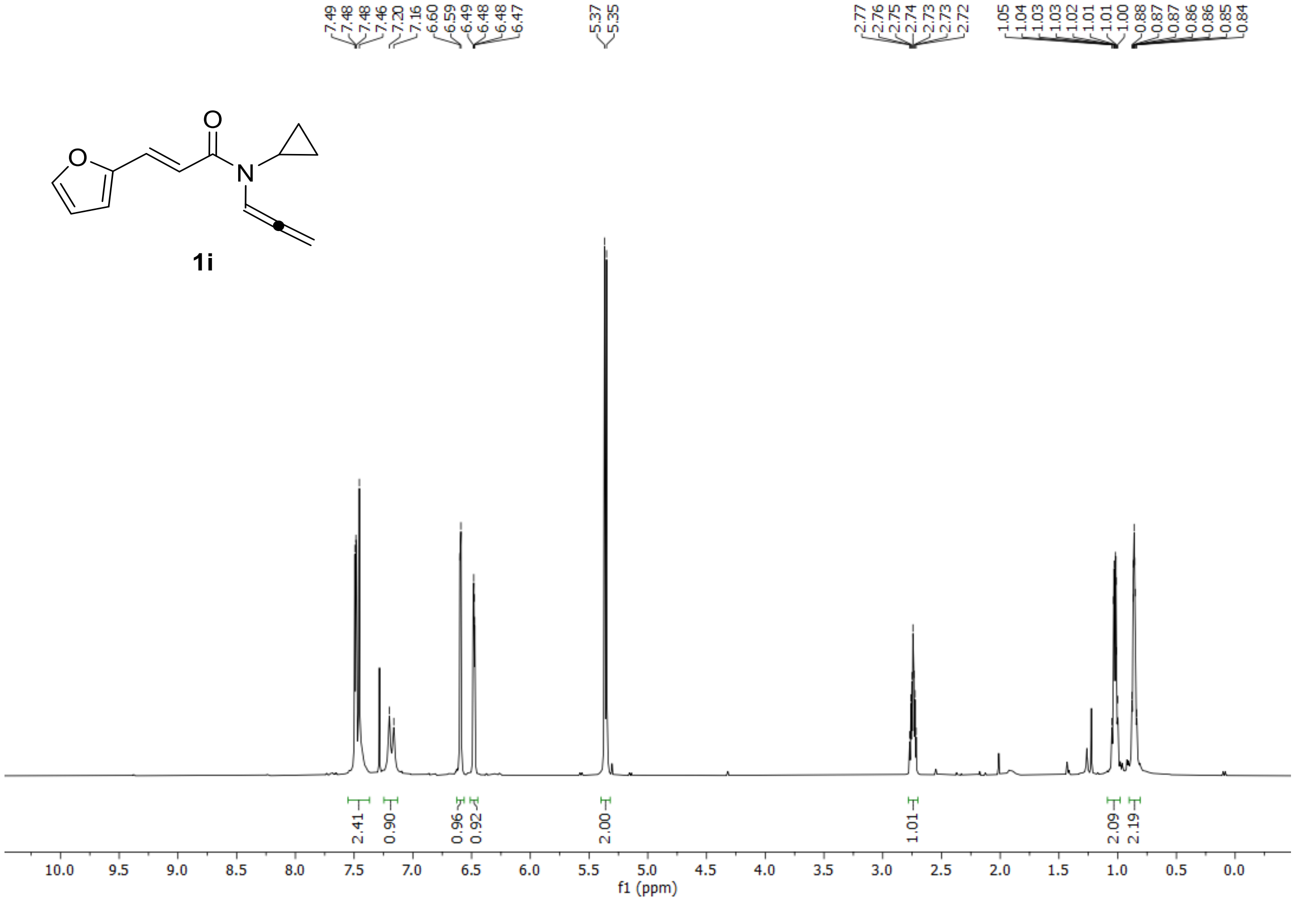

1i
7.49
7.48
7.48
7.46
7.20
7.16
6.60
6.59
6.49
6.48
6.48
6.47
5.37
5.35
2.77
2.76
2.75
2.74
2.73
2.73
2.72
1.05
1.04
1.03
1.03
1.02
1.01
1.01
1.00
0.88
0.87
0.87
0.86
0.86
0.85
0.84
2.41
0.90
0.96
0.92
2.00
1.01
2.09
2.19
10.0 9.5 9.0 8.5 8.0 7.5 7.0 6.5 6.0 5.5 5.0 4.5 4.0 3.5 3.0 2.5 2.0 1.5 1.0 0.5 0.0
f1 (ppm)


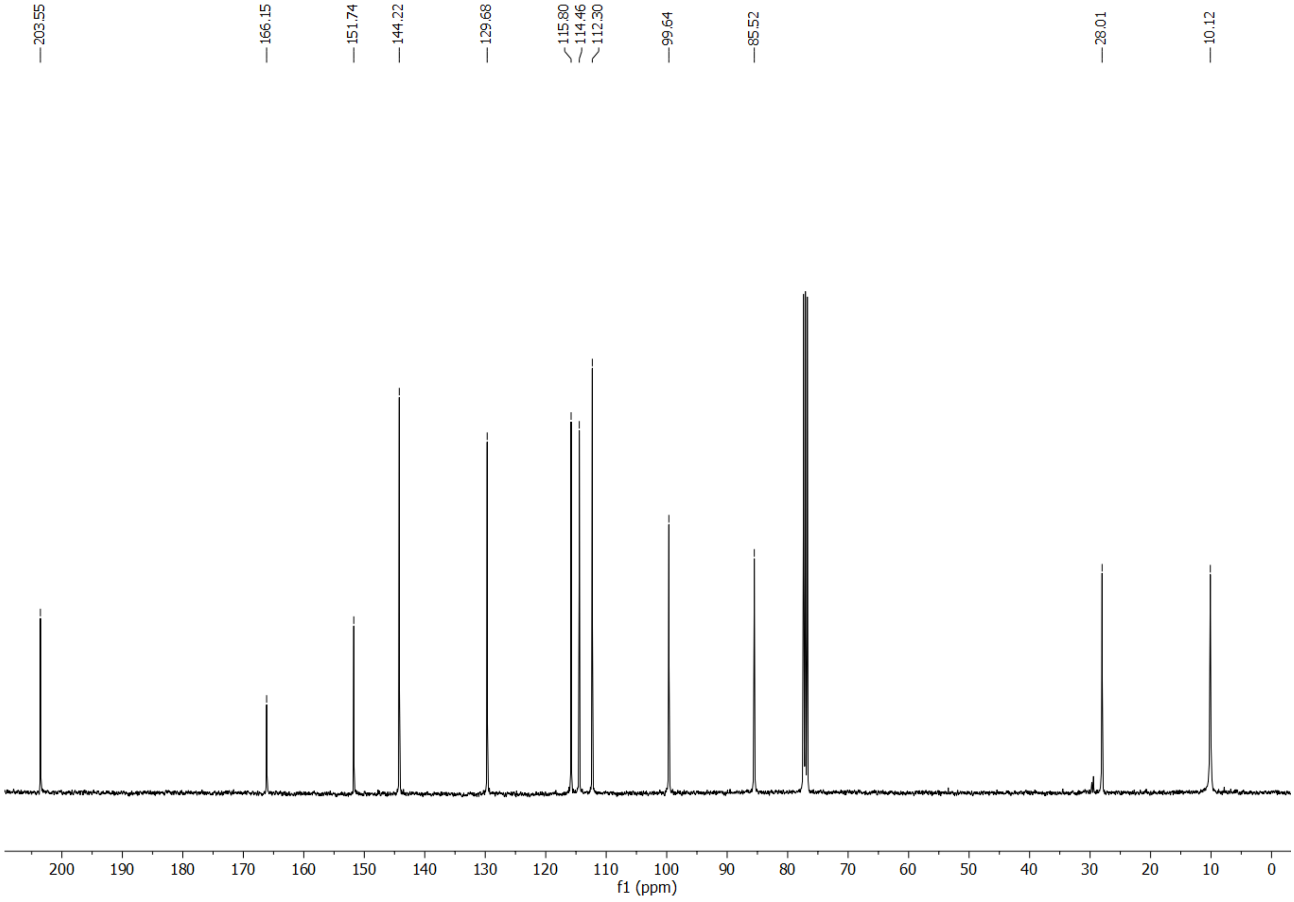

203.55
166.15
151.74
144.22
129.68
115.80
114.46
112.30
99.64
85.52
28.01
10.12
200 190 180 170 160 150 140 130 120 110 100 90 80 70 60 50 40 30 20 10 0
f1 (ppm)

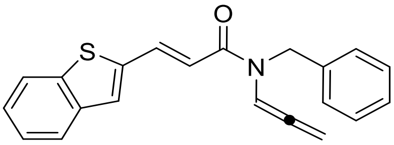
O
S
N

**1j**

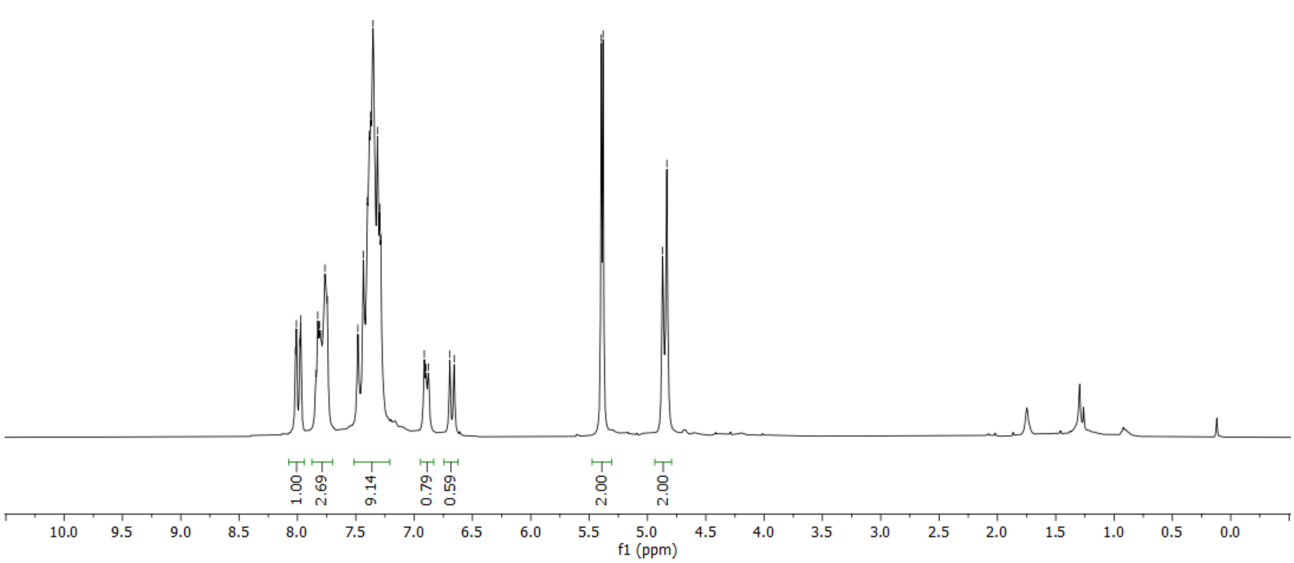
8.02
8.01
7.98
7.97
7.84
7.83
7.81
7.80
7.76
7.74
7.48
7.44
7.42
7.40
7.39
7.37
7.35
7.31
7.29
7.28
6.91
6.90
6.88
6.69
6.66
5.40
5.38
4.87
4.83
1.00
2.69
9.14
0.79
0.59
2.00
2.00
10.0 9.5 9.0 8.5 8.0 7.5 7.0 6.5 6.0 5.5 5.0 4.5 4.0 3.5 3.0 2.5 2.0 1.5 1.0 0.5 0.0
f1 (ppm)

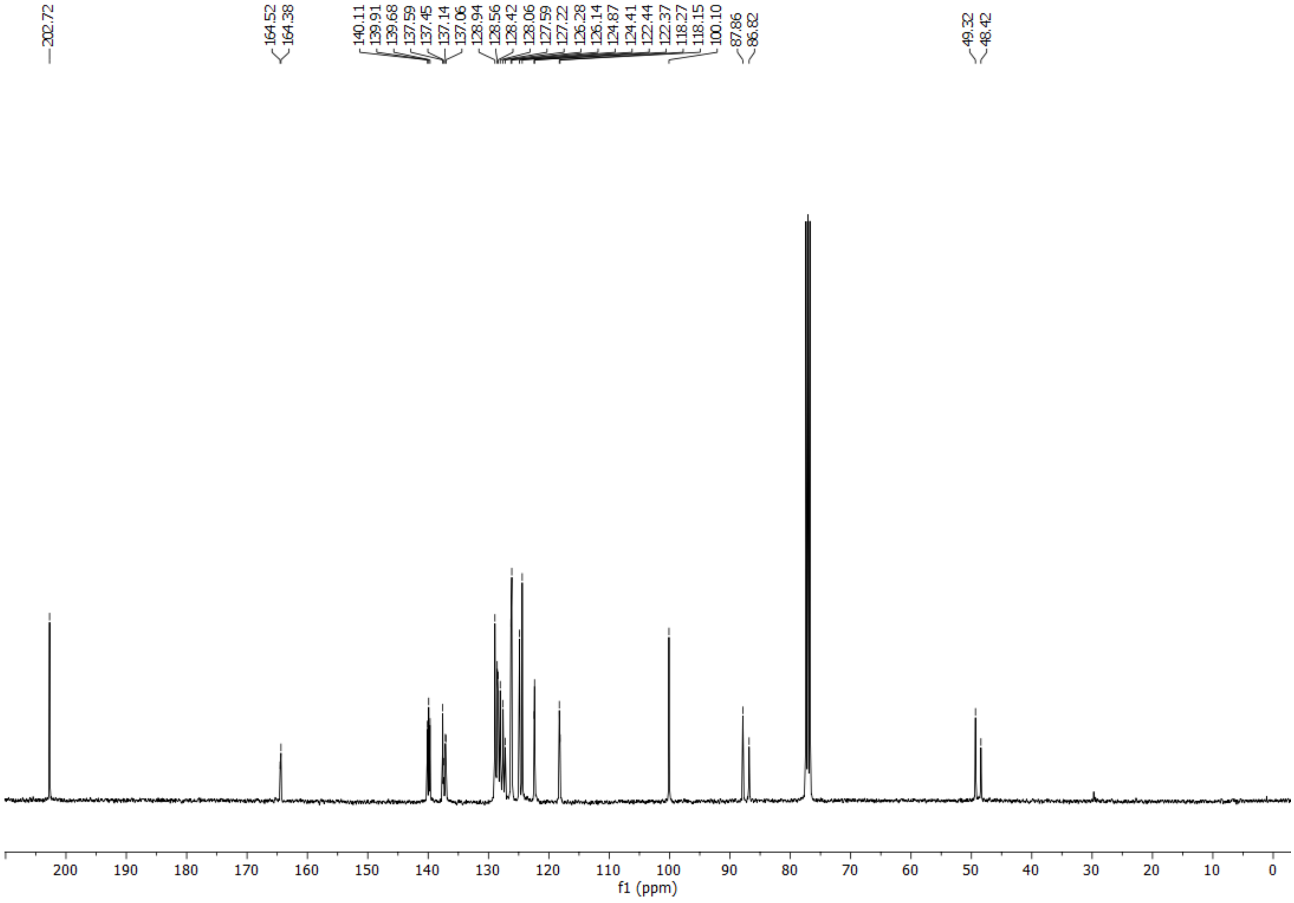
202.72
164.52
164.38
140.11
139.91
139.68
137.59
137.45
137.14
137.06
128.94
128.56
128.42
128.06
127.59
127.22
126.28
126.14
124.87
124.41
122.44
122.37
118.27
118.15
100.10
87.86
86.82
49.32
48.42
200 190 180 170 160 150 140 130 120 110 100 90 80 70 60 50 40 30 20 10 0
f1 (ppm)

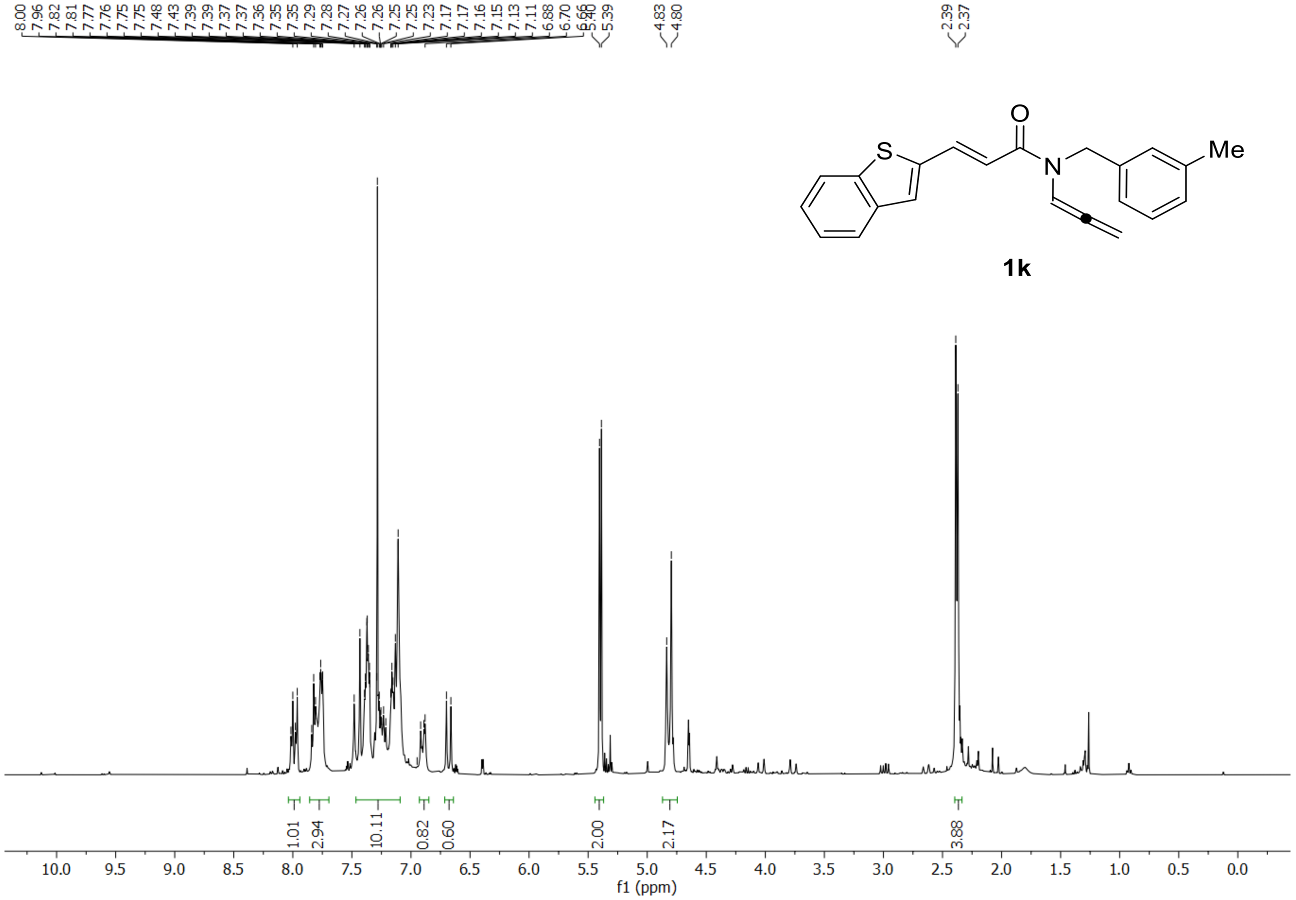
8.00
7.96
7.82
7.81
7.77
7.76
7.75
7.75
7.48
7.43
7.39
7.39
7.37
7.37
7.36
7.35
7.35
7.29
7.28
7.27
7.26
7.26
7.25
7.25
7.23
7.17
7.17
7.16
7.15
7.13
7.11
6.88
6.70
6.66
5.40
5.39
4.83
4.80
2.39
2.37
O
S
N
Me
1k
1.01
2.94
10.11
0.82
0.60
2.00
2.17
3.88
10.0 9.5 9.0 8.5 8.0 7.5 7.0 6.5 6.0 5.5 5.0 4.5 4.0 3.5 3.0 2.5 2.0 1.5 1.0 0.5 0.0
f1 (ppm)

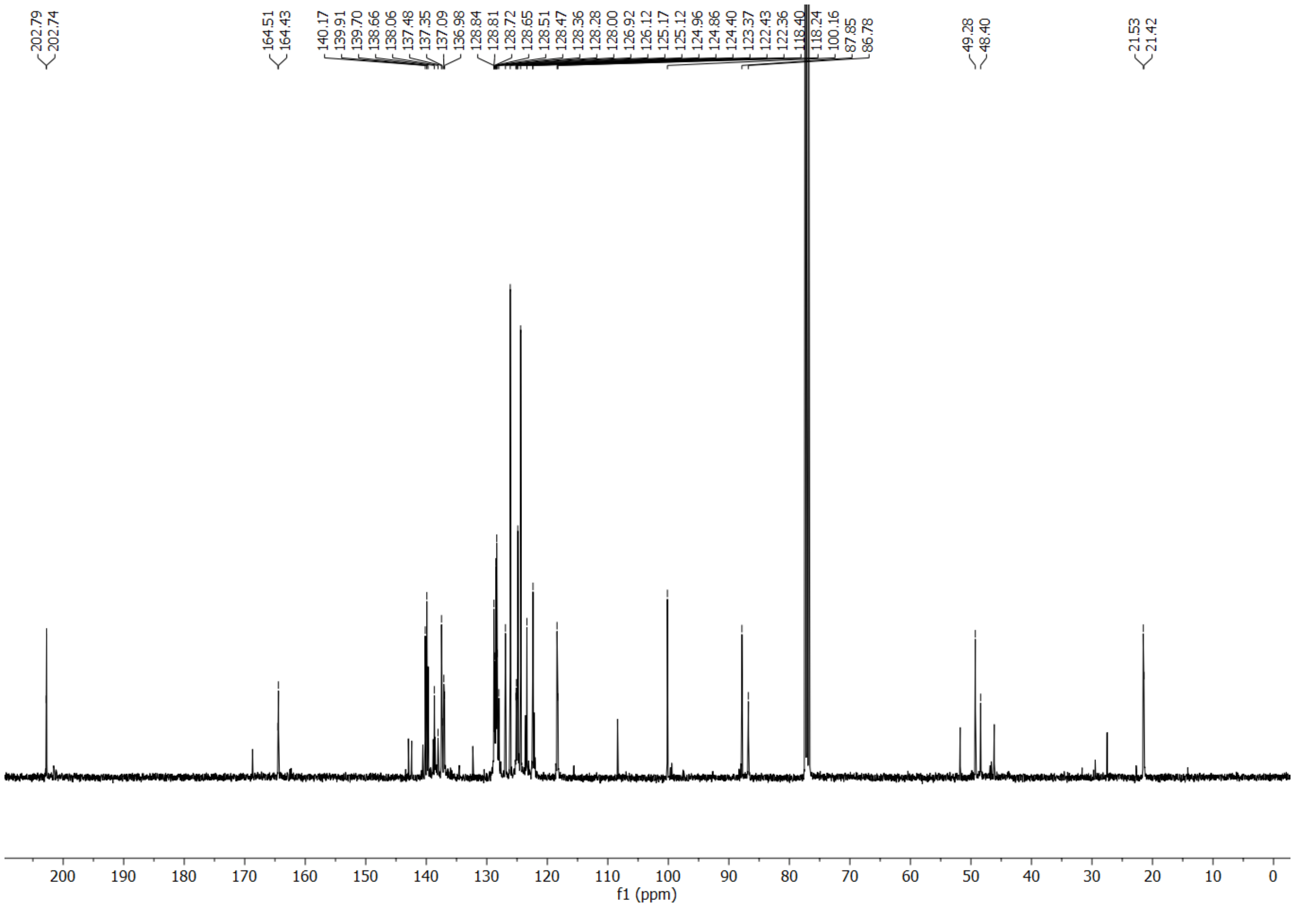
202.79
202.74
164.51
164.43
140.17
139.91
139.70
138.66
138.06
137.48
137.35
137.09
136.98
128.84
128.81
128.72
128.65
128.51
128.47
128.36
128.28
128.00
126.92
126.12
125.17
125.12
124.96
124.86
124.40
123.37
122.43
122.36
118.40
118.24
100.16
87.85
86.78
49.28
48.40
21.53
21.42
200 190 180 170 160 150 140 130 120 110 100 90 80 70 60 50 40 30 20 10 0
f1 (ppm)

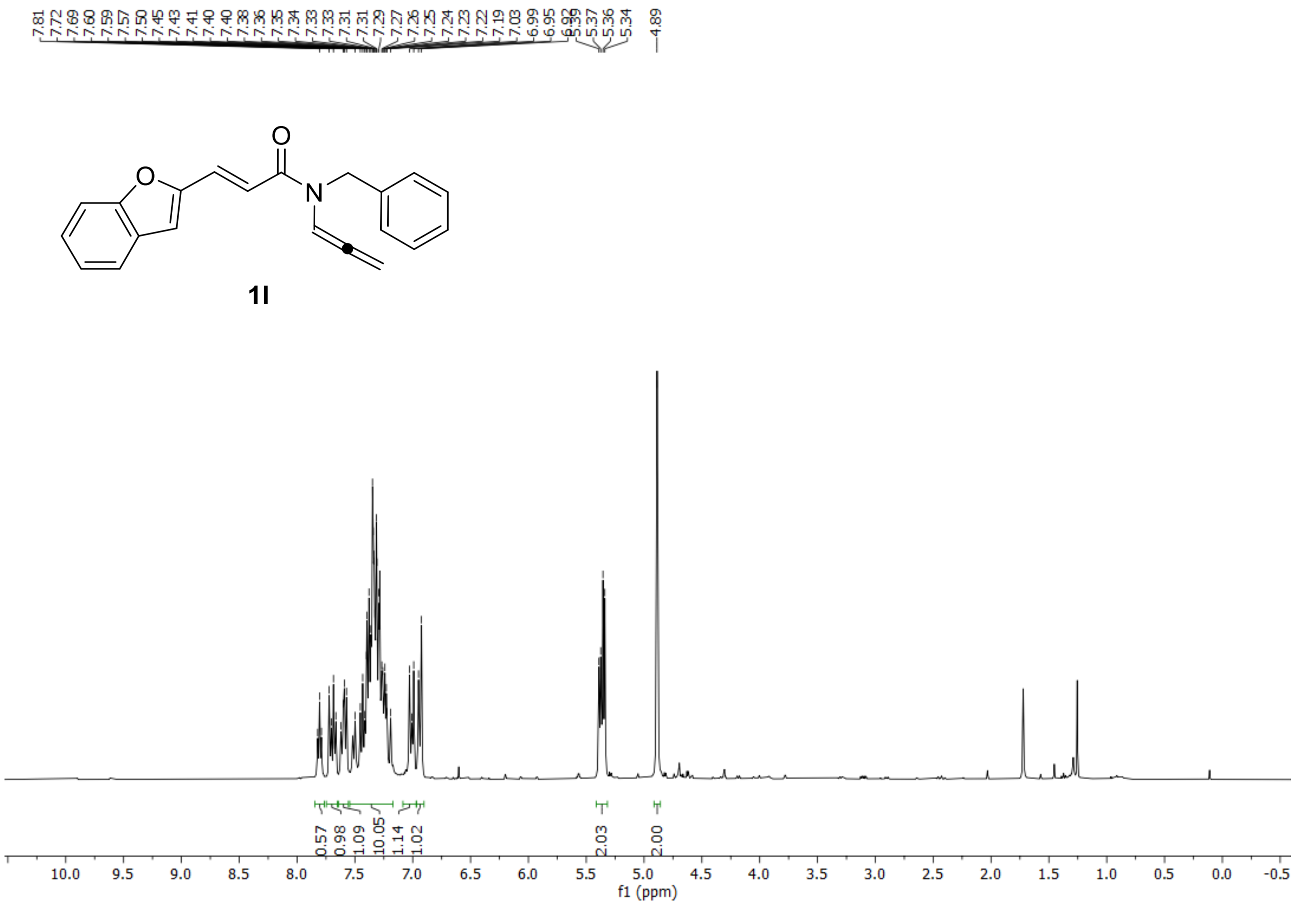
1l
7.81
7.72
7.69
7.60
7.59
7.57
7.50
7.45
7.43
7.41
7.40
7.38
7.36
7.35
7.34
7.33
7.33
7.31
7.31
7.29
7.27
7.26
7.25
7.24
7.23
7.22
7.19
7.03
6.99
6.95
6.92
5.39
5.37
5.36
5.34
4.89
0.57
0.98
1.09
10.05
1.14
1.02
2.03
2.00
10.0 9.5 9.0 8.5 8.0 7.5 7.0 6.5 6.0 5.5 5.0 4.5 4.0 3.5 3.0 2.5 2.0 1.5 1.0 0.5 0.0 -0.5
f1 (ppm)

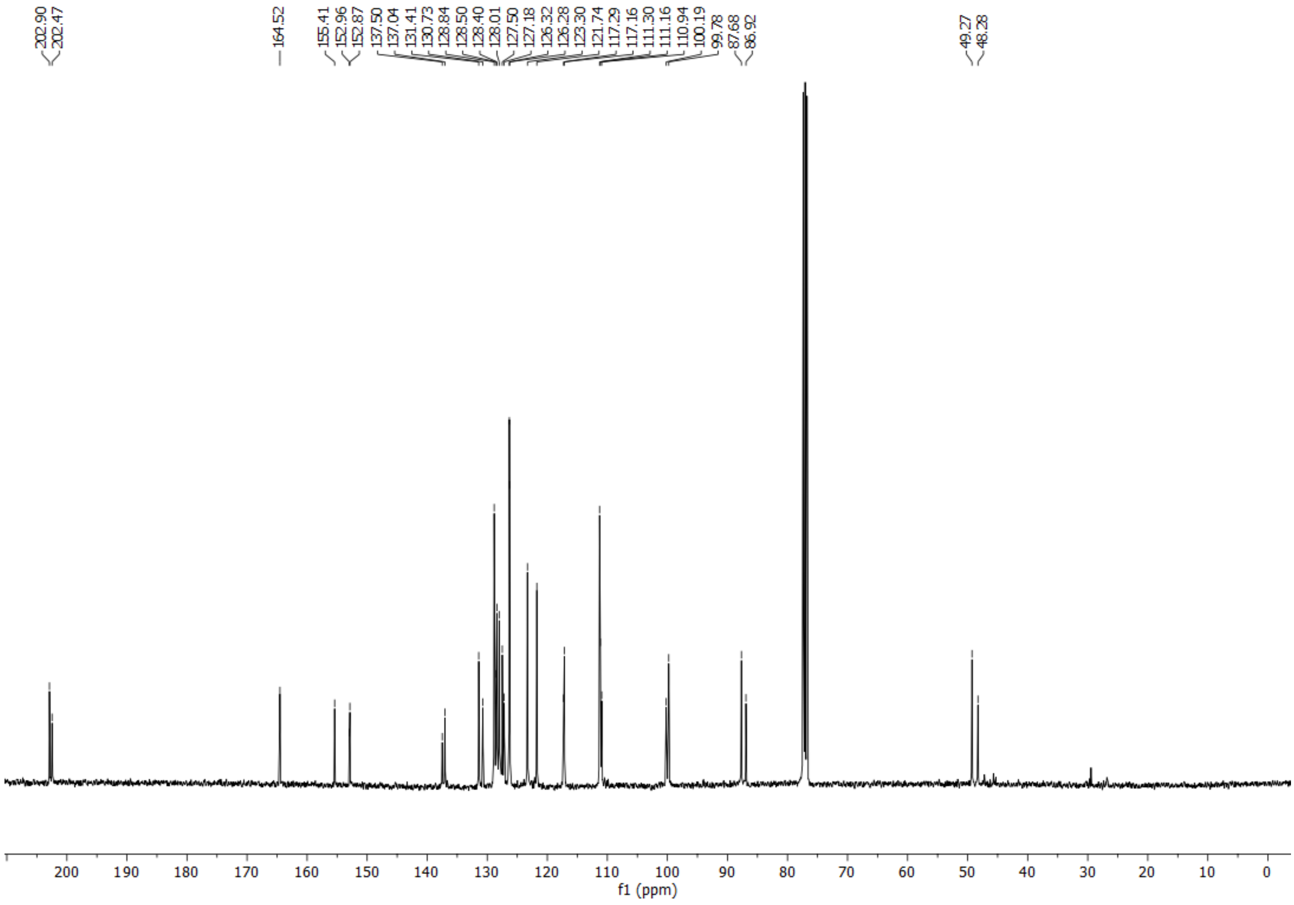
202.90
202.47
164.52
155.41
152.96
152.87
137.50
137.04
131.41
130.73
128.84
128.50
128.40
128.01
127.50
127.18
126.32
126.28
123.30
121.74
117.29
117.16
111.30
111.16
110.94
100.19
99.78
87.68
86.92
49.27
48.28
200 190 180 170 160 150 140 130 120 110 100 90 80 70 60 50 40 30 20 10 0
f1 (ppm)

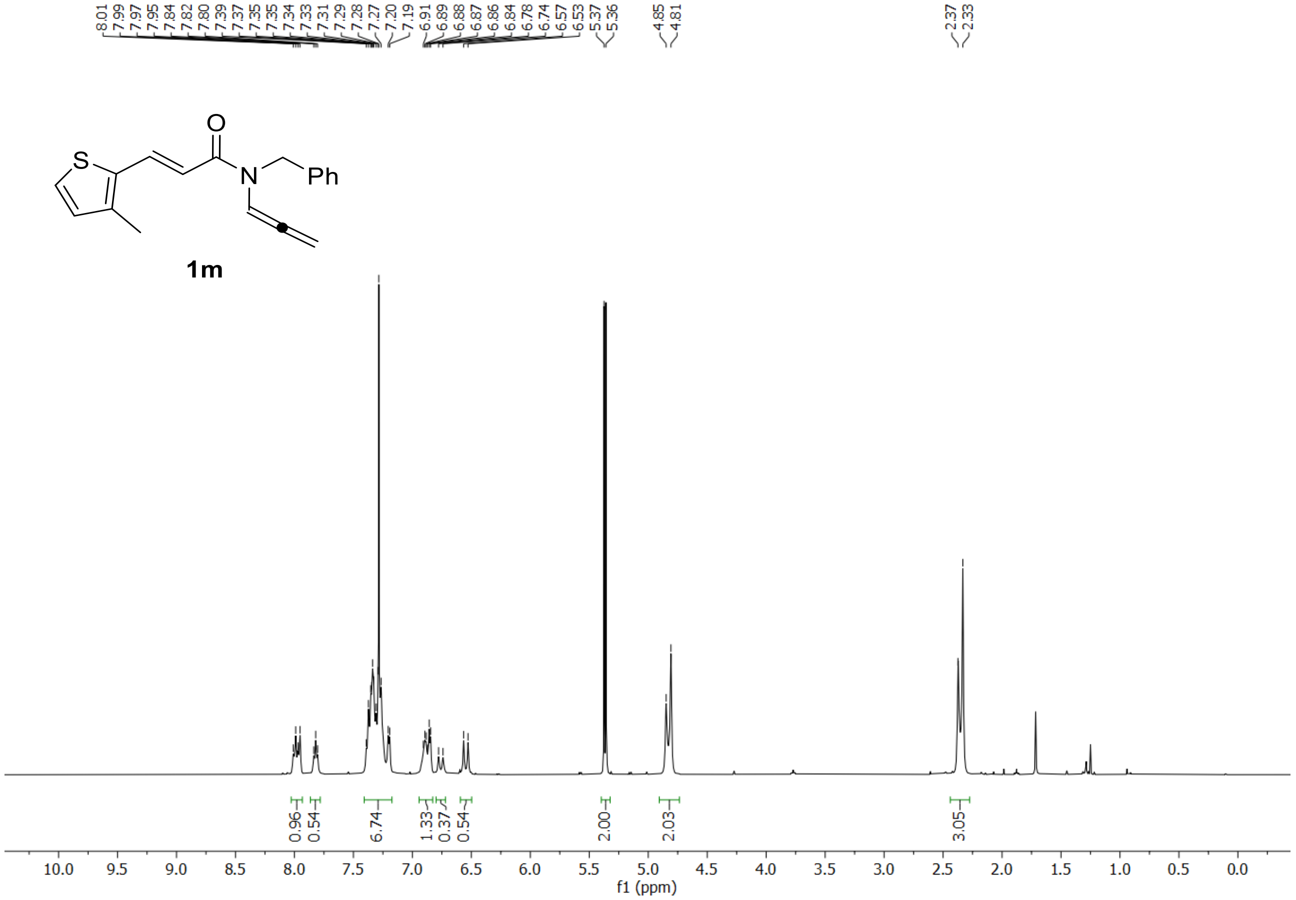
1m
8.01 7.99 7.97 7.95 7.84 7.82 7.80 7.39 7.37 7.35 7.35 7.34 7.33 7.31 7.29 7.28 7.27 7.20 7.19 6.91 6.89 6.88 6.87 6.86 6.84 6.78 6.74 6.57 6.53 5.37 5.36
4.85 4.81
2.37 2.33
0.96 0.54 6.74 1.33 0.37 0.54 2.00 2.03 3.05
10.0 9.5 9.0 8.5 8.0 7.5 7.0 6.5 6.0 5.5 5.0 4.5 4.0 3.5 3.0 2.5 2.0 1.5 1.0 0.5 0.0
f1 (ppm)

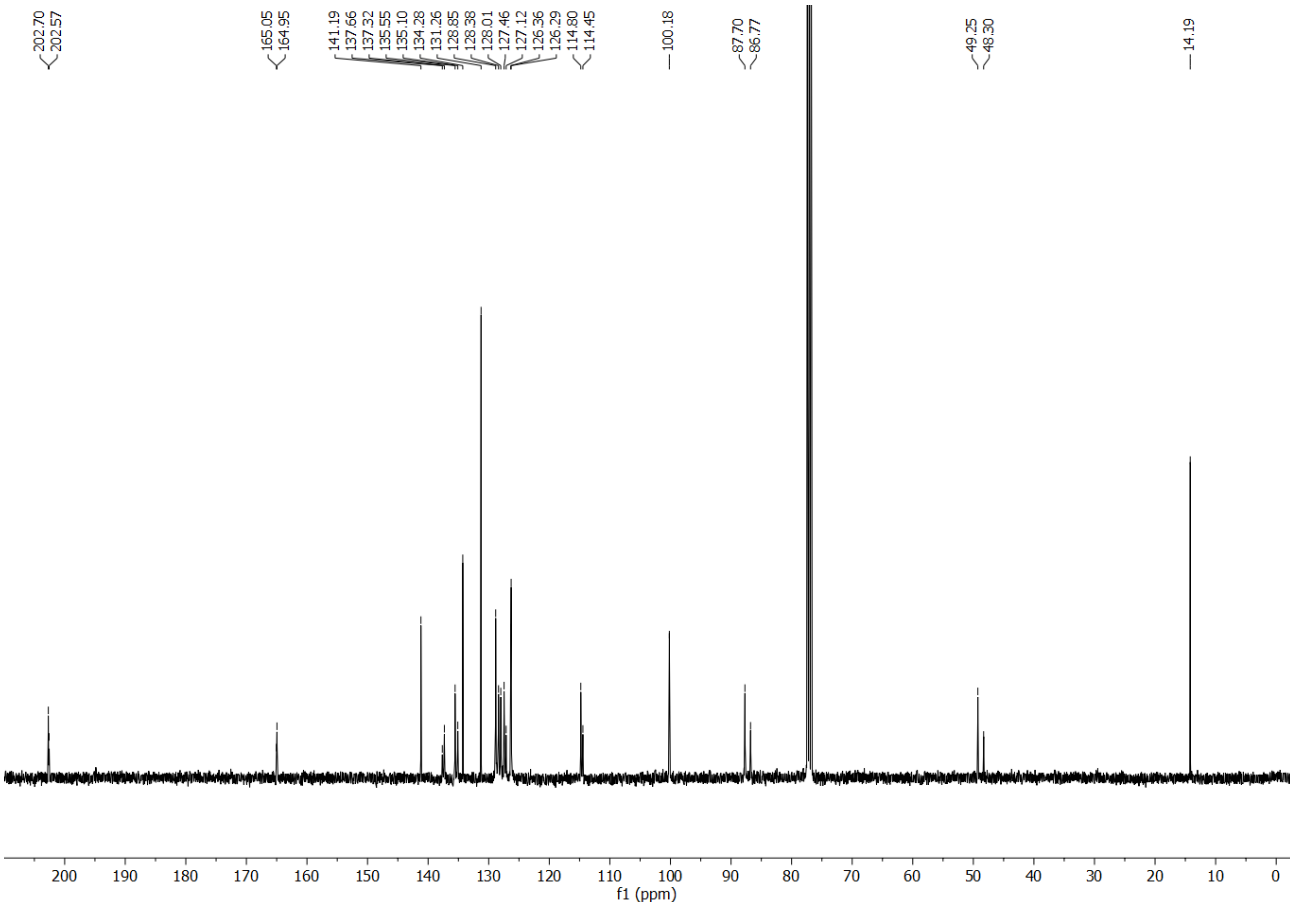
202.70 202.57
165.05 164.95
141.19 137.66 137.32 135.55 135.10 134.28 131.26 128.85 128.38 128.01 127.46 127.12 126.36 126.29 114.80 114.45
100.18
87.70 86.77
49.25 48.30
14.19
200 190 180 170 160 150 140 130 120 110 100 90 80 70 60 50 40 30 20 10 0
f1 (ppm)

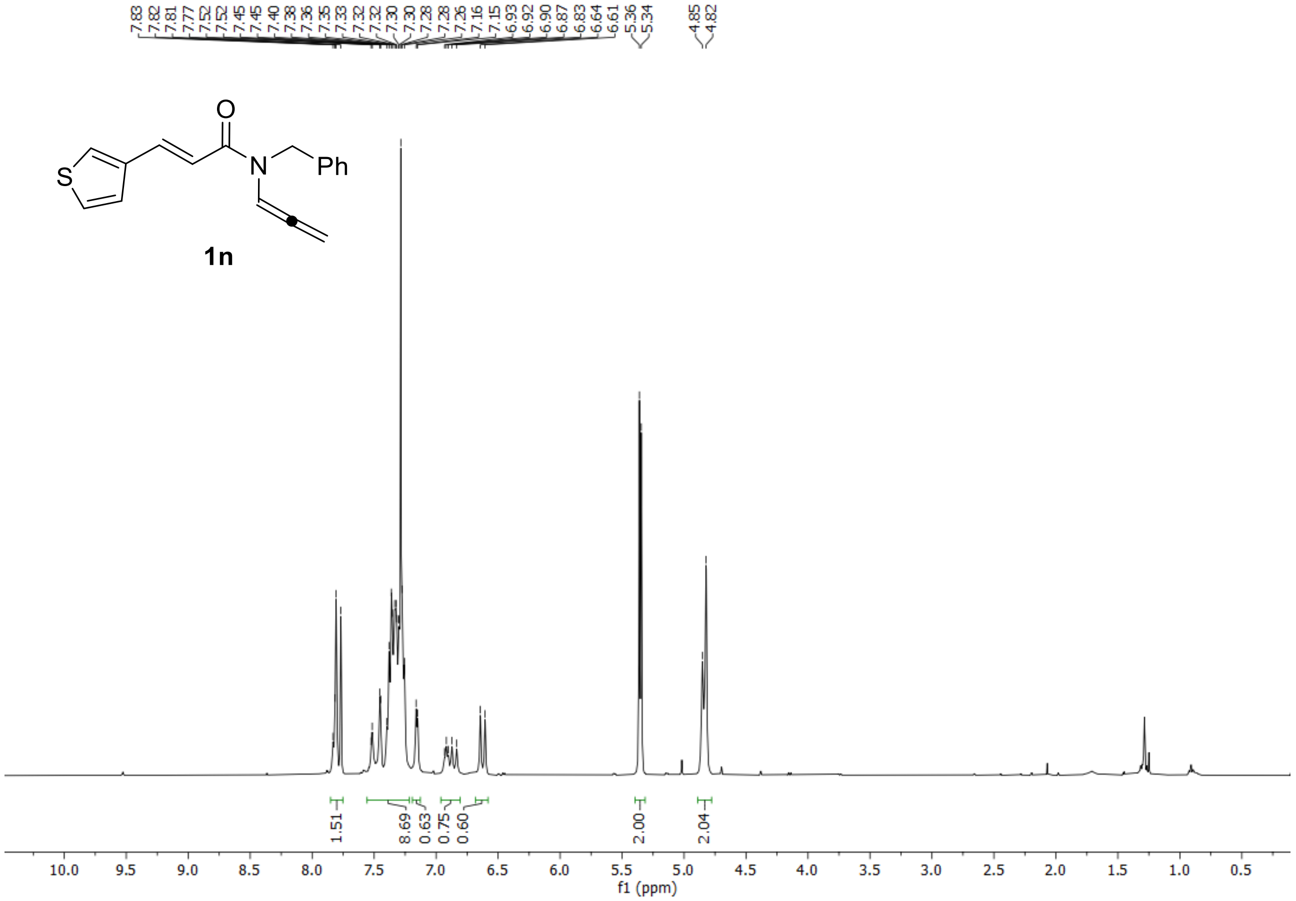

7.83 7.82 7.81 7.77 7.52 7.52 7.45 7.45 7.40 7.38 7.36 7.35 7.33 7.32 7.32 7.30 7.30 7.28 7.28 7.26 7.16 7.15 6.93 6.92 6.90 6.87 6.83 6.64 6.61 5.36 5.34 4.85 4.82
1.51
8.69
0.63
0.75
0.60
2.00
2.04
10.0 9.5 9.0 8.5 8.0 7.5 7.0 6.5 6.0 5.5 5.0 4.5 4.0 3.5 3.0 2.5 2.0 1.5 1.0 0.5
f1 (ppm)


**1n**

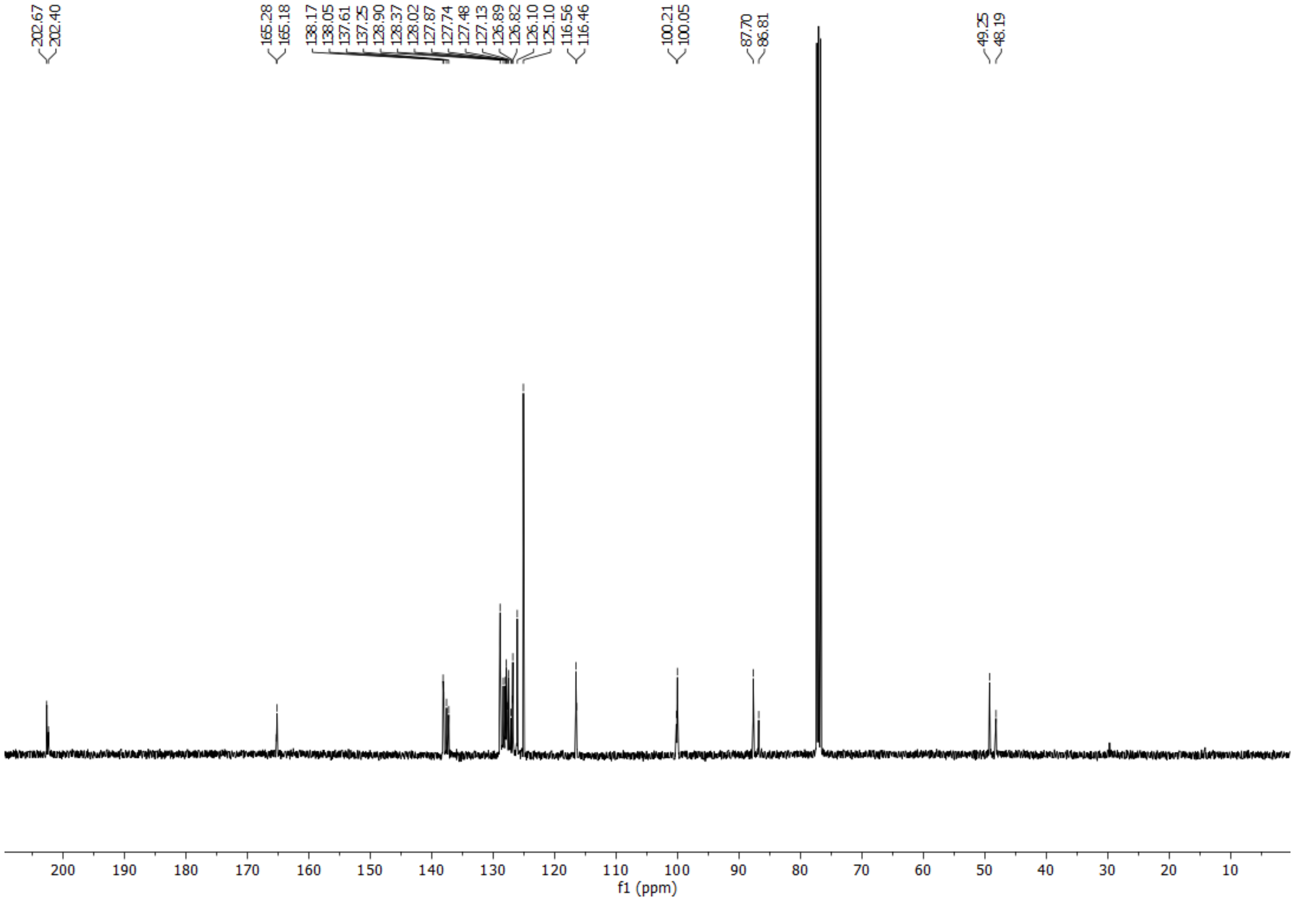

202.67 202.40 165.28 165.18 138.17 138.05 137.61 137.25 128.90 128.37 128.02 127.87 127.74 127.48 127.13 126.89 126.82 126.10 125.10 116.56 116.46 100.21 100.05 87.70 86.81 49.25 48.19
200 190 180 170 160 150 140 130 120 110 100 90 80 70 60 50 40 30 20 10
f1 (ppm)

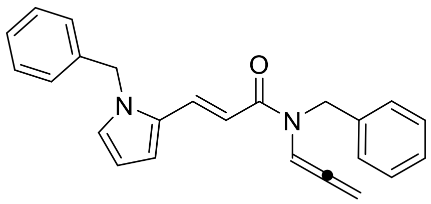
O
N
N

**1o**

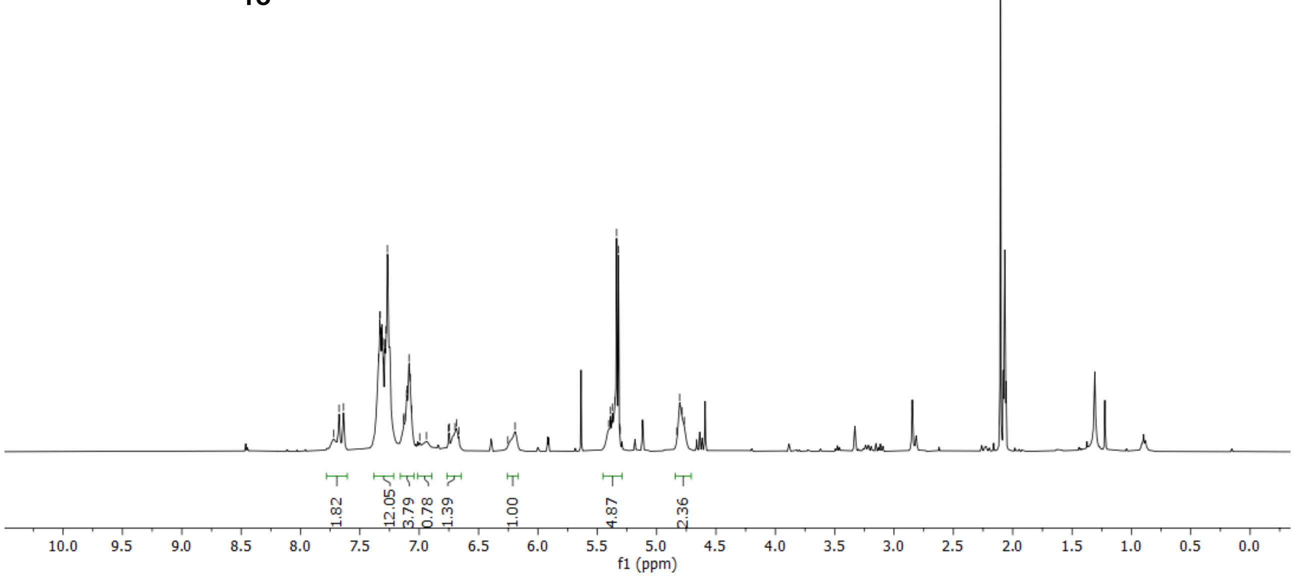
7.72
7.68
7.64
7.34
7.34
7.33
7.33
7.32
7.31
7.31
7.31
7.30
7.29
7.29
7.29
7.28
7.27
7.26
7.13
7.13
7.11
7.10
7.09
7.08
7.06
7.00
6.94
6.75
6.75
6.70
6.69
6.67
6.25
6.19
5.40
5.39
5.37
5.35
5.34
5.32
5.31
5.31
4.83
4.81
4.79
4.77
1.82
12.05
3.79
0.78
1.39
1.00
4.87
2.36
10.0 9.5 9.0 8.5 8.0 7.5 7.0 6.5 6.0 5.5 5.0 4.5 4.0 3.5 3.0 2.5 2.0 1.5 1.0 0.5 0.0
f1 (ppm)

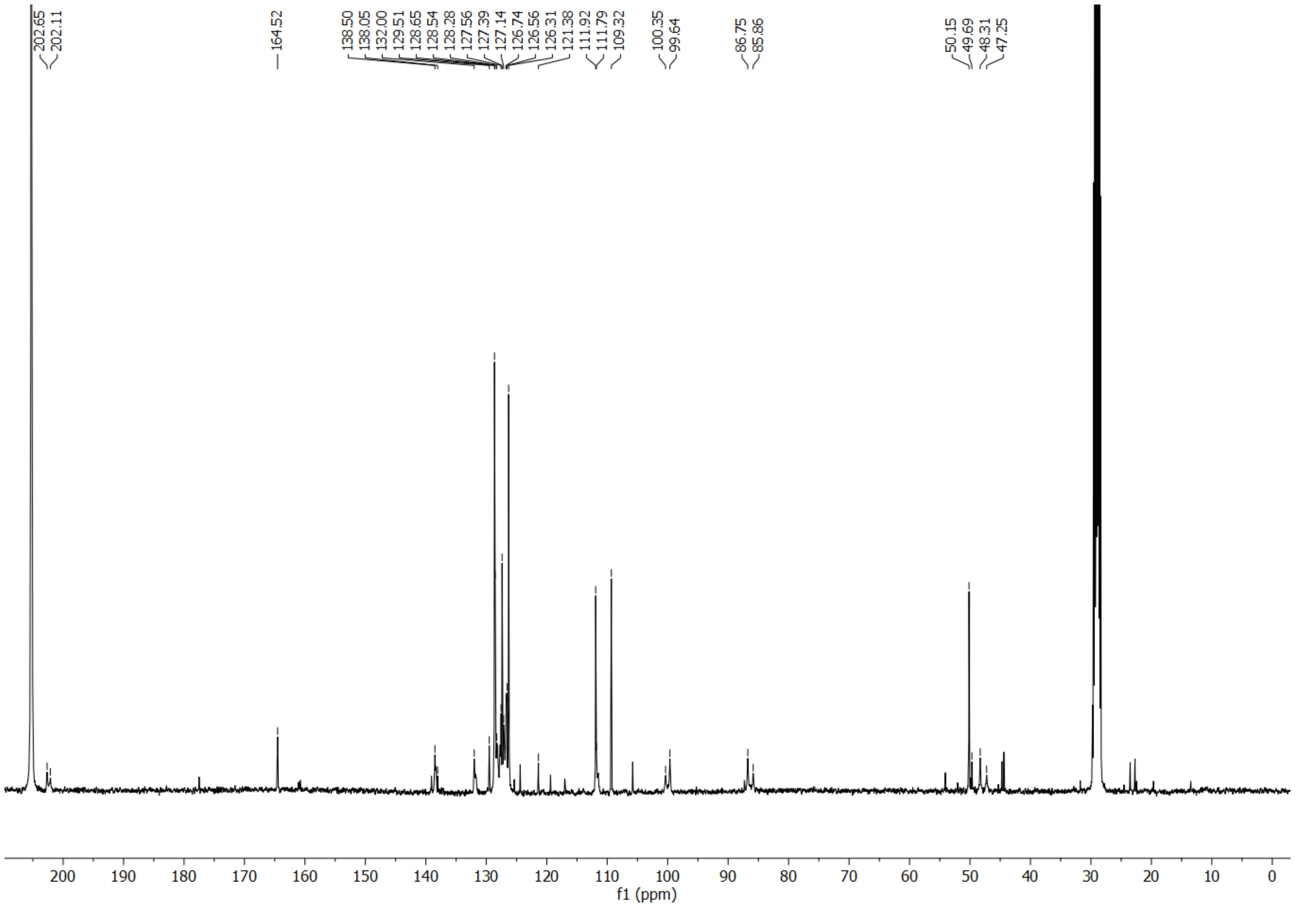
202.65
202.11
164.52
138.50
138.05
132.00
129.51
128.65
128.54
128.28
127.56
127.39
127.14
126.74
126.56
126.31
121.38
111.92
111.79
109.32
100.35
99.64
86.75
85.86
50.15
49.69
48.31
47.25
200 190 180 170 160 150 140 130 120 110 100 90 80 70 60 50 40 30 20 10 0
f1 (ppm)

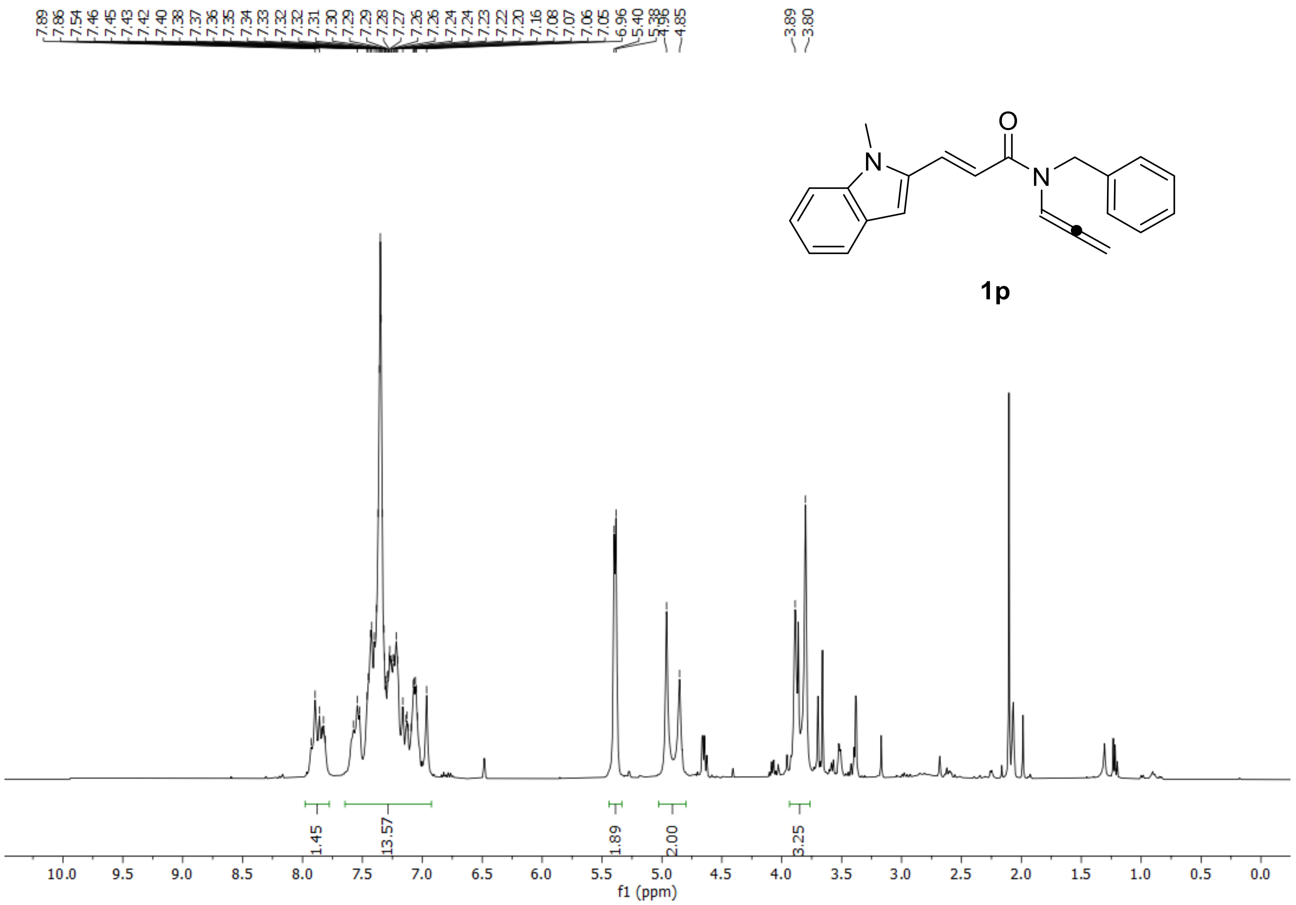
1p
1.45
13.57
1.89
2.00
3.25
f1 (ppm)

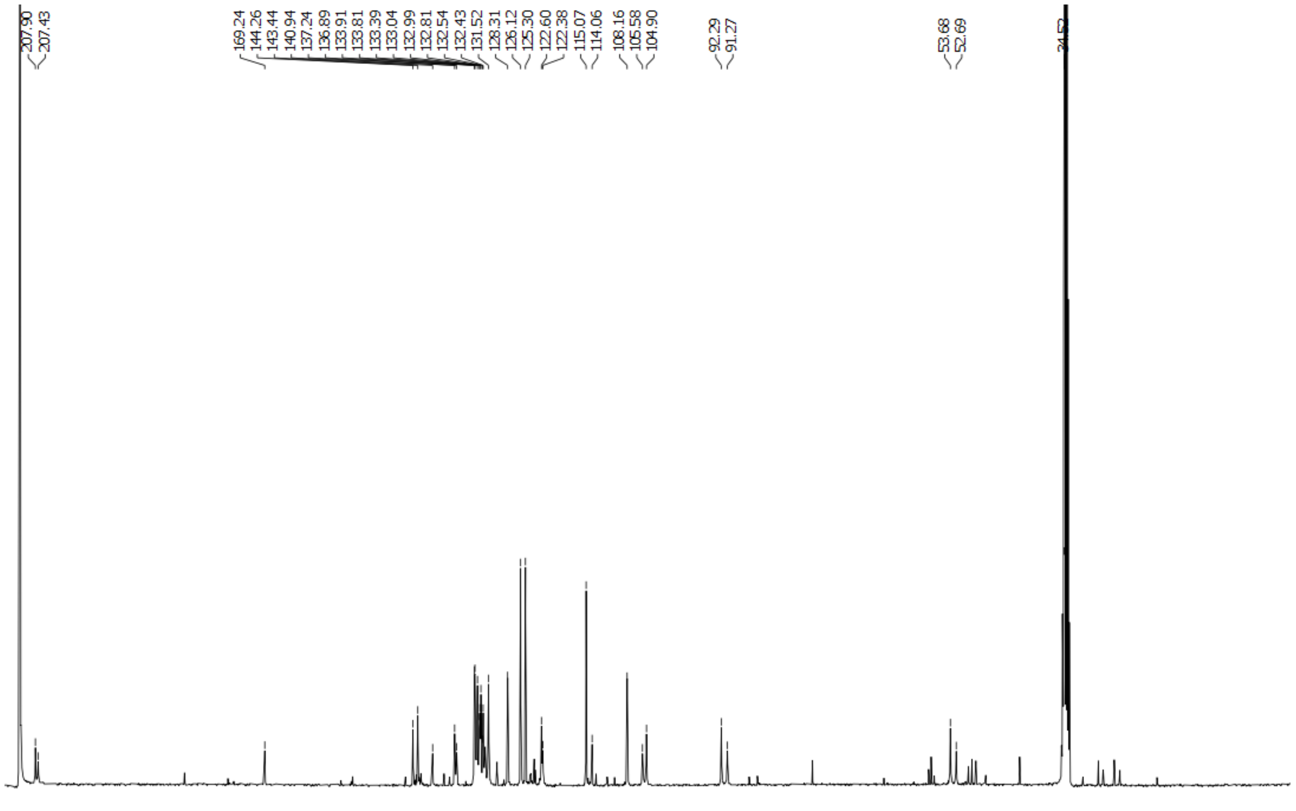
f1 (ppm)

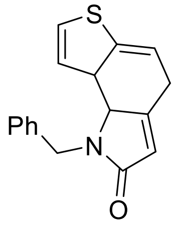
S
Ph
N
O

**2a**

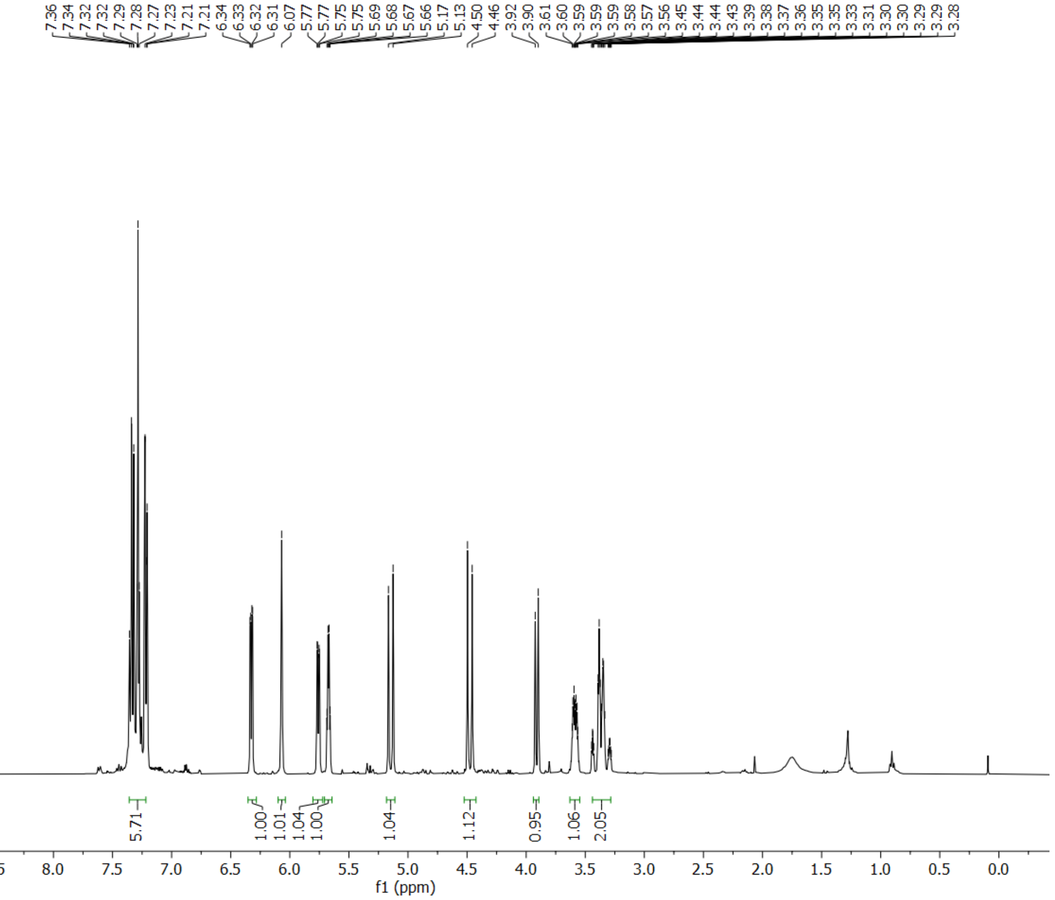
7.36
7.34
7.32
7.32
7.29
7.28
7.27
7.23
7.21
7.21
6.34
6.33
6.32
6.31
6.07
5.77
5.77
5.75
5.75
5.69
5.68
5.67
5.66
5.17
5.13
4.50
4.46
3.92
3.90
3.61
3.60
3.59
3.59
3.59
3.58
3.57
3.56
3.45
3.44
3.44
3.43
3.39
3.38
3.37
3.36
3.35
3.35
3.33
3.31
3.30
3.30
3.29
3.29
3.28
5.71
1.00
1.01
1.04
1.00
1.04
1.12
0.95
1.06
2.05
10.0 9.5 9.0 8.5 8.0 7.5 7.0 6.5 6.0 5.5 5.0 4.5 4.0 3.5 3.0 2.5 2.0 1.5 1.0 0.5 0.0
f1 (ppm)

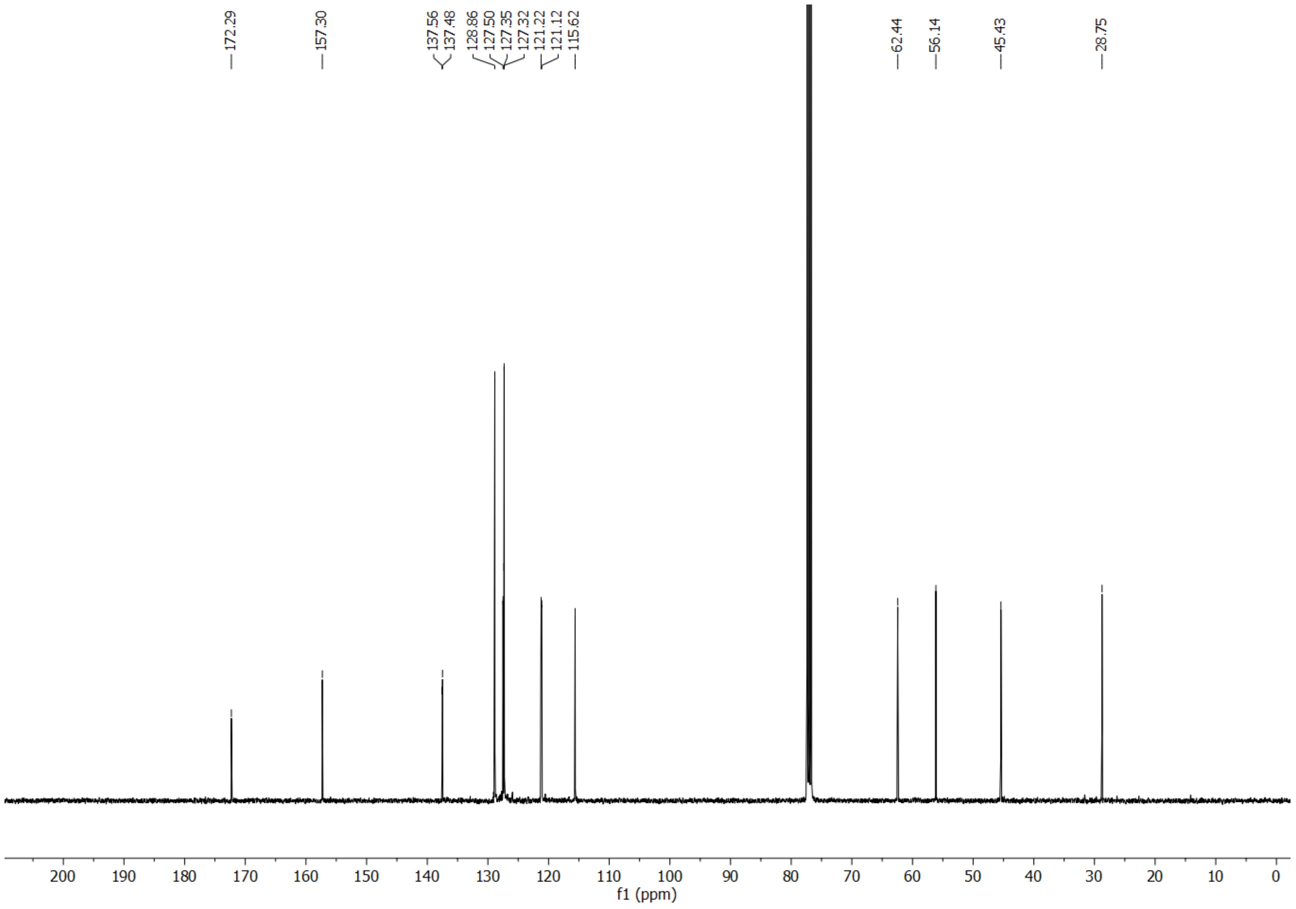
172.29
157.30
137.56
137.48
128.86
127.50
127.35
127.32
121.22
121.12
115.62
62.44
56.14
45.43
28.75
200 190 180 170 160 150 140 130 120 110 100 90 80 70 60 50 40 30 20 10 0
f1 (ppm)

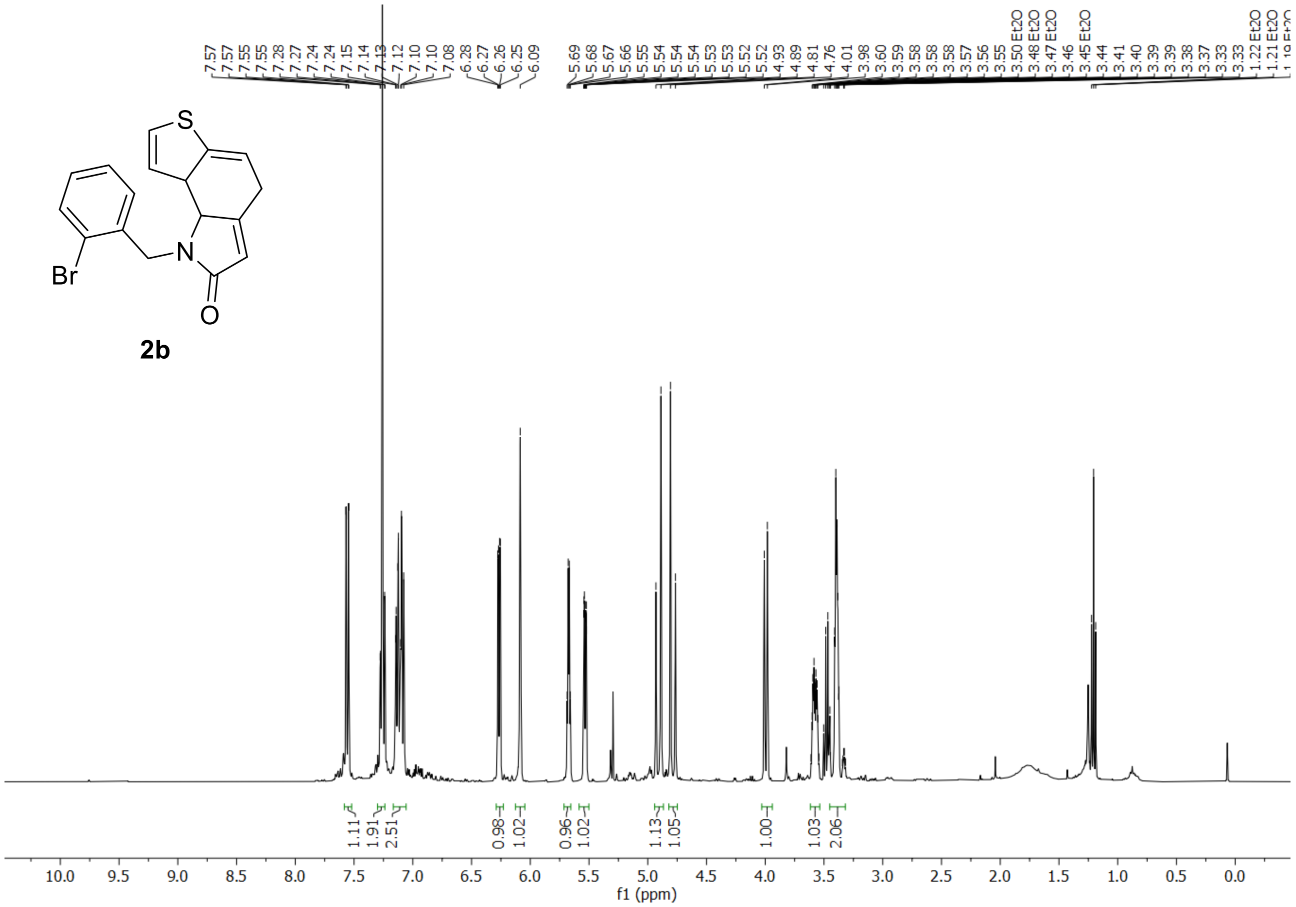
S
N
Br
O
2b
7.57 7.57 7.55 7.55 7.28 7.27 7.24 7.24 7.15 7.14 7.13 7.12 7.10 7.10 7.08 6.28 6.27 6.26 6.25 6.09 5.69 5.68 5.67 5.66 5.55 5.54 5.54 5.54 5.53 5.53 5.52 5.52 4.93 4.89 4.81 4.76 4.01 3.98 3.60 3.59 3.58 3.58 3.58 3.57 3.56 3.55 3.50 Et2O 3.48 Et2O 3.47 Et2O 3.46 3.45 Et2O 3.44 3.41 3.40 3.39 3.39 3.38 3.37 3.33 3.33 1.22 Et2O 1.21 Et2O
1.11 1.91 2.51 0.98 1.02 0.96 1.02 1.13 1.05 1.00 1.03 2.06
10.0 9.5 9.0 8.5 8.0 7.5 7.0 6.5 6.0 5.5 5.0 4.5 4.0 3.5 3.0 2.5 2.0 1.5 1.0 0.5 0.0
f1 (ppm)

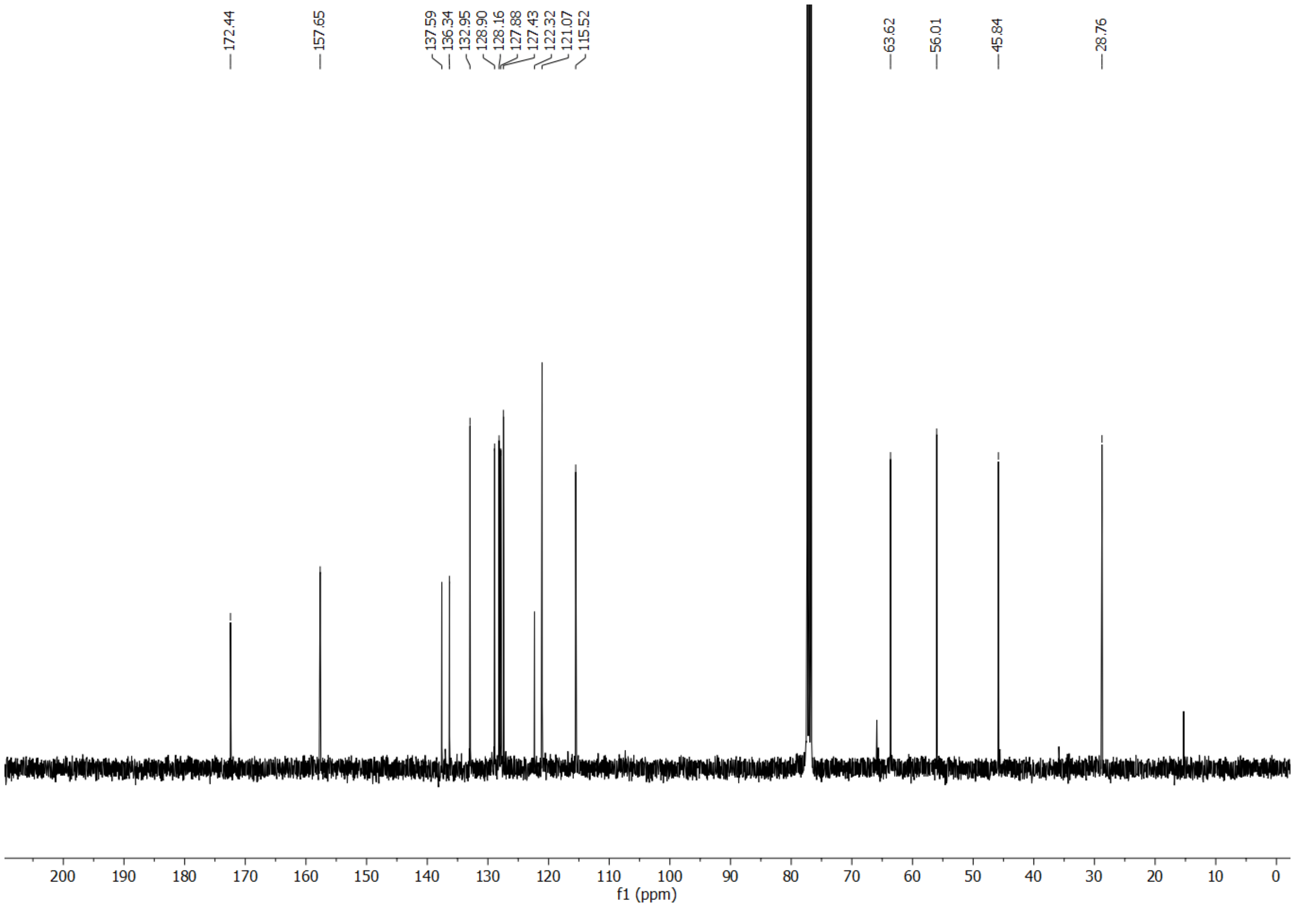
172.44 157.65 137.59 136.34 132.95 128.90 128.16 127.88 127.43 122.32 121.07 115.52 63.62 56.01 45.84 28.76
200 190 180 170 160 150 140 130 120 110 100 90 80 70 60 50 40 30 20 10 0
f1 (ppm)

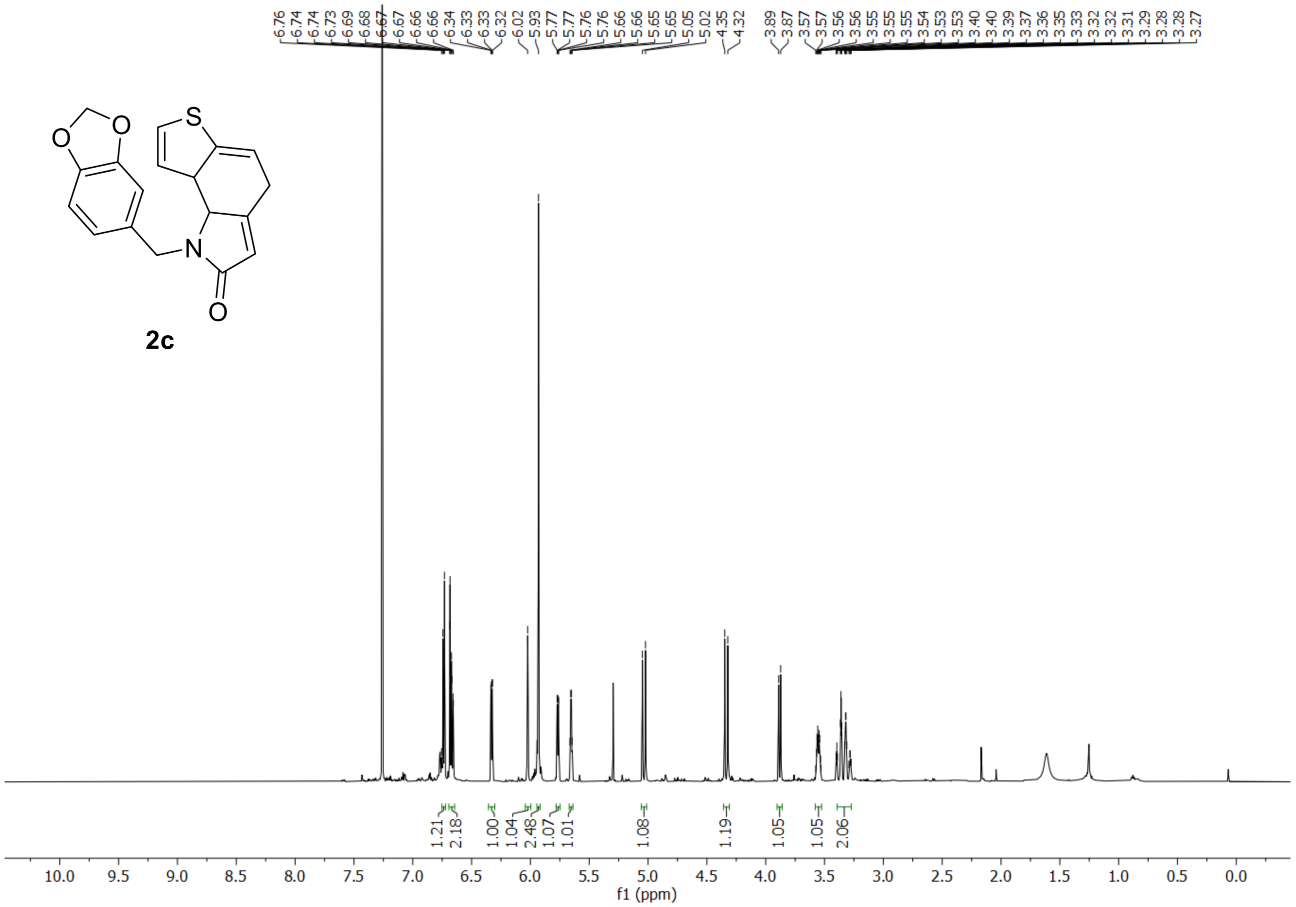

**2c**
6.76 6.74 6.74 6.73 6.69 6.68 6.67 6.67 6.66 6.66 6.34 6.33 6.33 6.32 6.02 5.93 5.77 5.77 5.76 5.76 5.66 5.66 5.65 5.65 5.05 5.02 4.35 4.32 3.89 3.87 3.57 3.57 3.56 3.56 3.55 3.55 3.55 3.54 3.53 3.53 3.40 3.39 3.37 3.36 3.35 3.33 3.32 3.32 3.31 3.29 3.28 3.28 3.27
1.21 2.18 1.00 1.04 2.48 1.07 1.01 1.08 1.19 1.05 1.05 2.06
10.0 9.5 9.0 8.5 8.0 7.5 7.0 6.5 6.0 5.5 5.0 4.5 4.0 3.5 3.0 2.5 2.0 1.5 1.0 0.5 0.0
f1 (ppm)

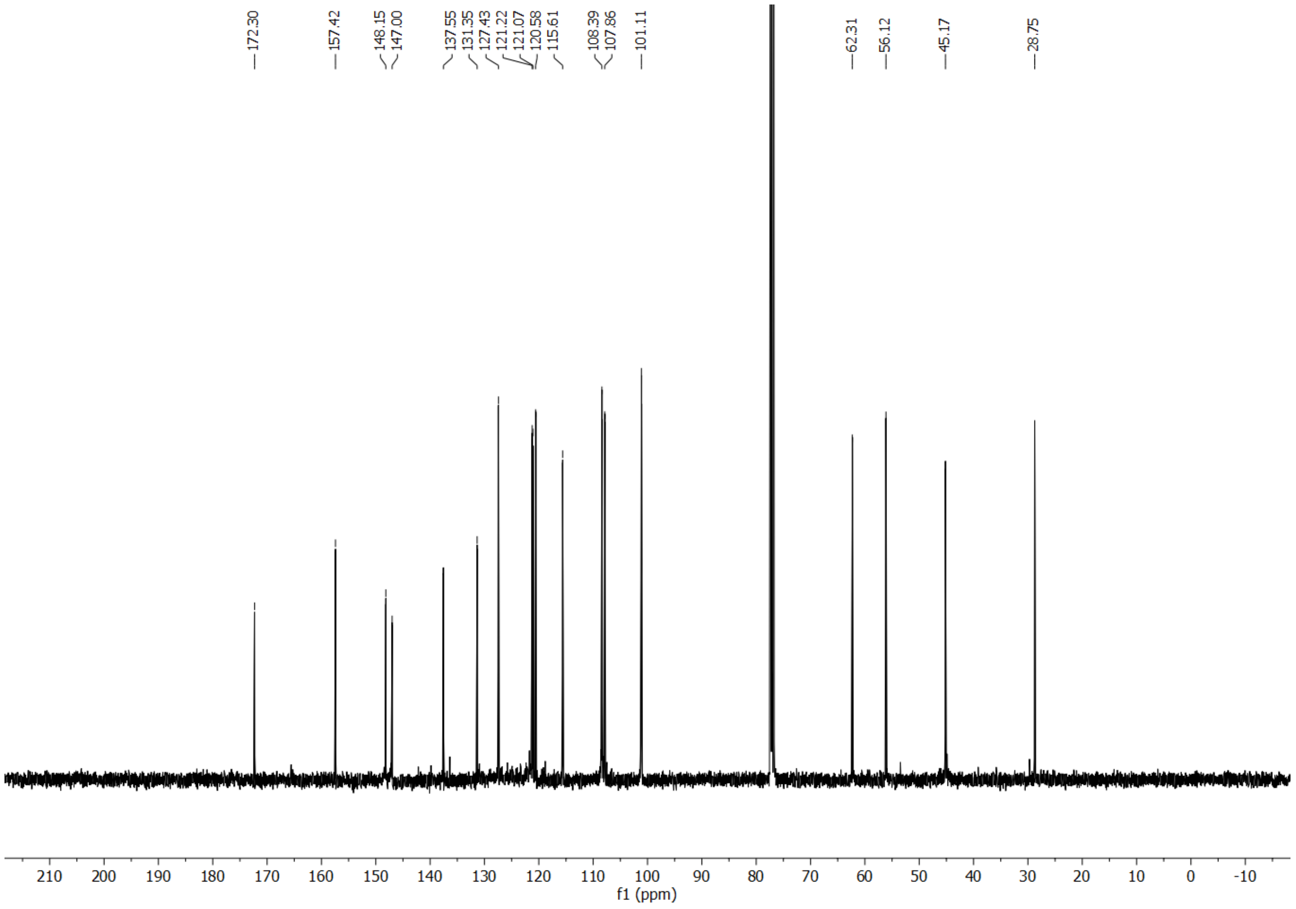
172.30 157.42 148.15 147.00 137.55 131.35 127.43 121.22 121.07 120.58 115.61 108.39 107.86 101.11 62.31 56.12 45.17 28.75
210 200 190 180 170 160 150 140 130 120 110 100 90 80 70 60 50 40 30 20 10 0 -10
f1 (ppm)

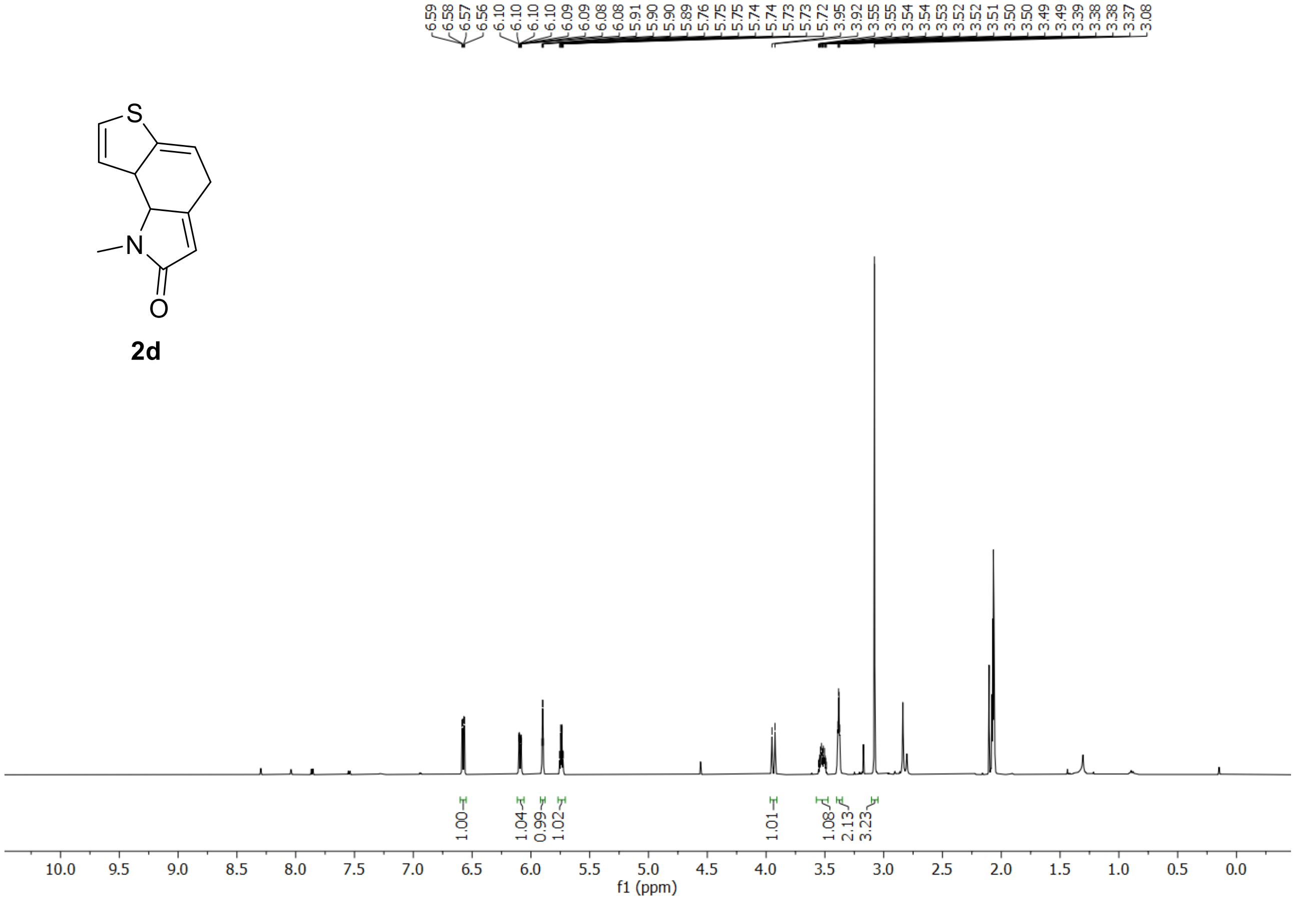
6.59
6.58
6.57
6.56
6.10
6.10
6.10
6.10
6.09
6.08
6.08
5.91
5.90
5.90
5.89
5.76
5.75
5.75
5.74
5.74
5.73
5.73
5.72
3.95
3.92
3.55
3.55
3.54
3.54
3.53
3.52
3.52
3.51
3.50
3.50
3.49
3.49
3.39
3.38
3.38
3.37
3.08
S
N
O
2d
1.00
1.04
0.99
1.02
1.01
1.08
2.13
3.23
f1 (ppm)

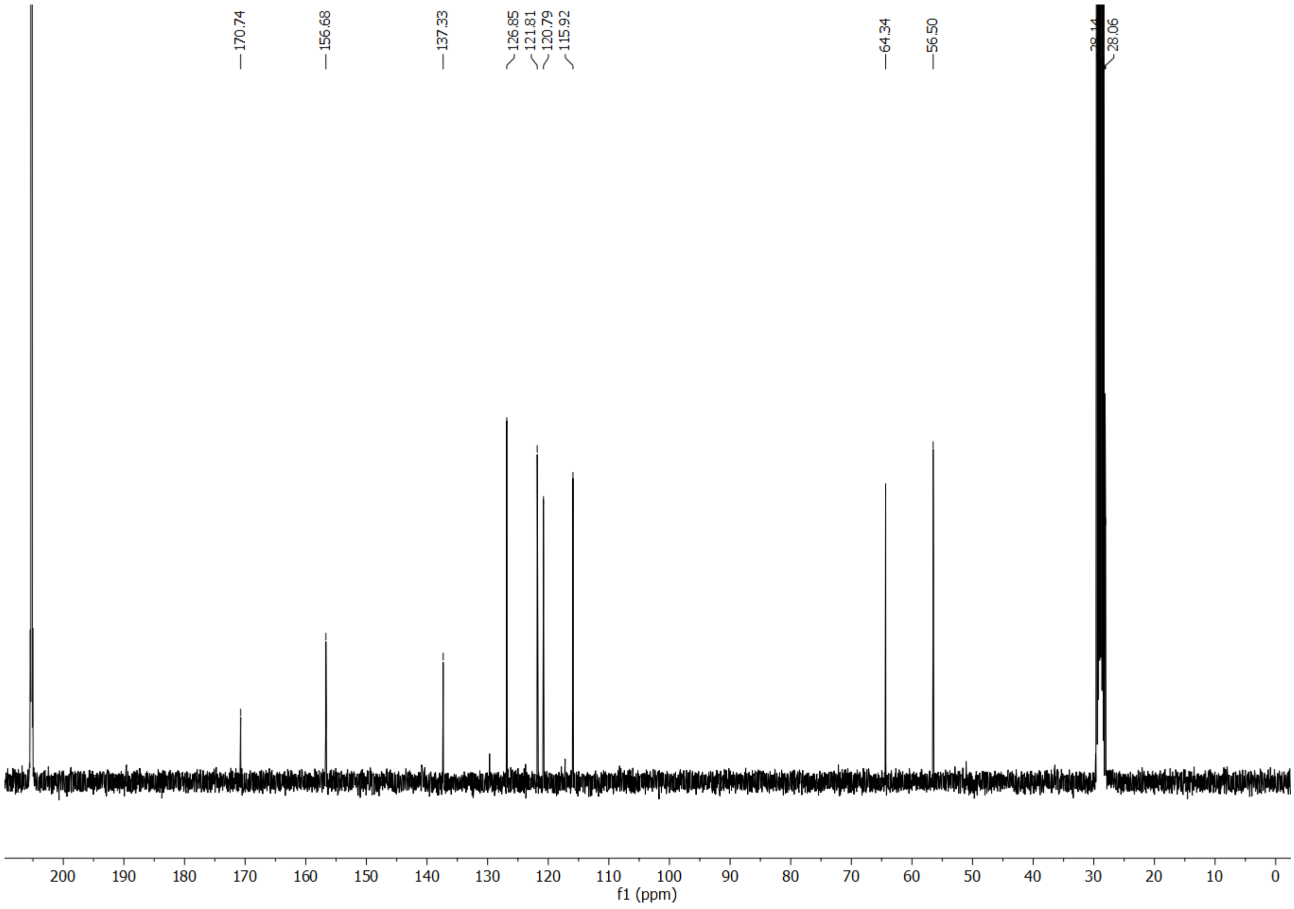
170.74
156.68
137.33
126.85
121.81
120.79
115.92
64.34
56.50
28.06
f1 (ppm)

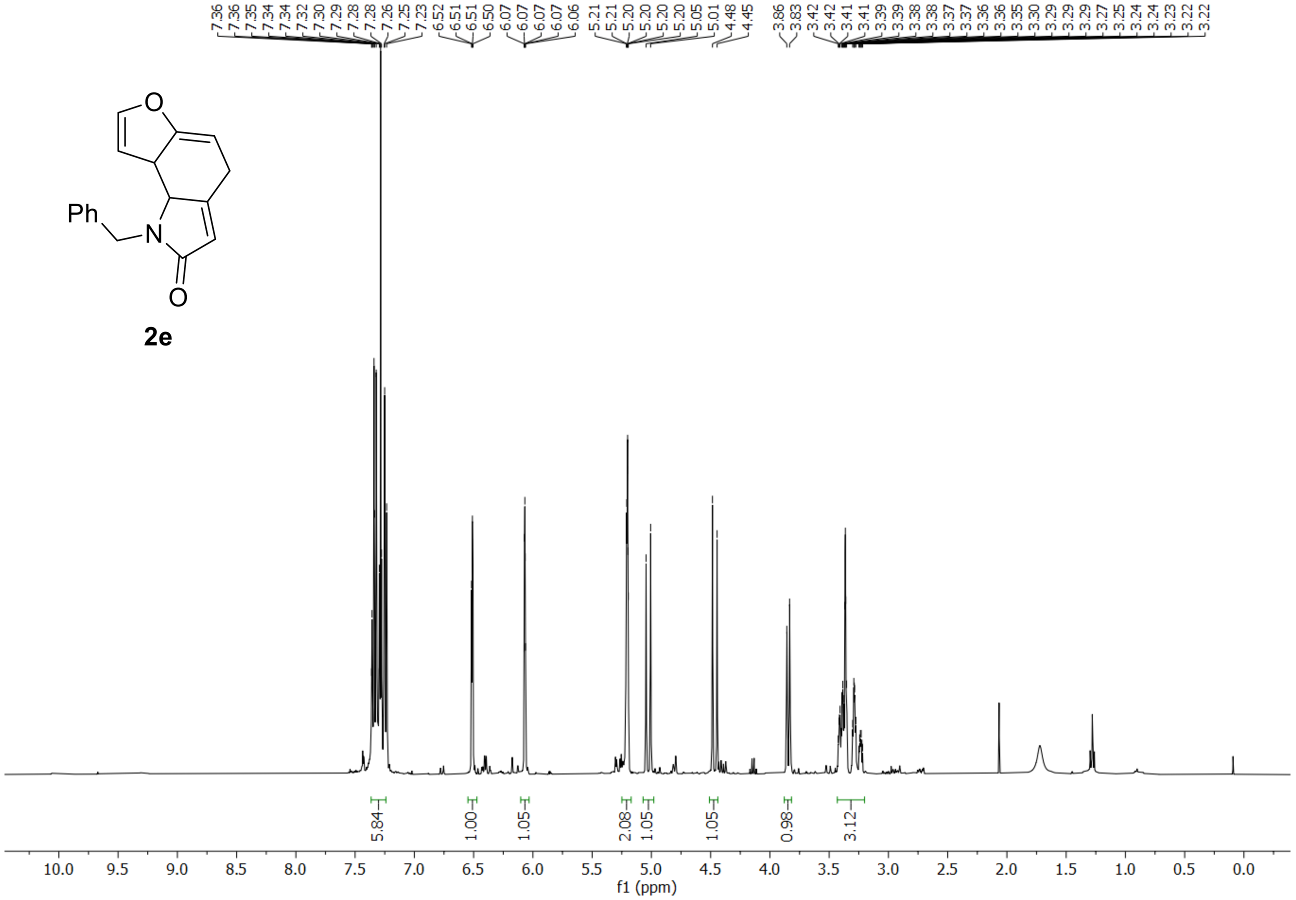
O
Ph
N
O
2e
7.36 7.36 7.35 7.34 7.34 7.32 7.30 7.29 7.28 7.28 7.26 7.25 7.23 6.52 6.51 6.51 6.50 6.07 6.07 6.07 6.07 6.06 5.21 5.21 5.20 5.20 5.20 5.20 5.05 5.01 4.48 4.45 3.86 3.83 3.42 3.41 3.41 3.39 3.39 3.38 3.38 3.37 3.37 3.36 3.36 3.35 3.30 3.29 3.29 3.29 3.27 3.25 3.24 3.24 3.23 3.22 3.22
5.84 1.00 1.05 2.08 1.05 1.05 0.98 3.12
10.0 9.5 9.0 8.5 8.0 7.5 7.0 6.5 6.0 5.5 5.0 4.5 4.0 3.5 3.0 2.5 2.0 1.5 1.0 0.5 0.0
f1 (ppm)

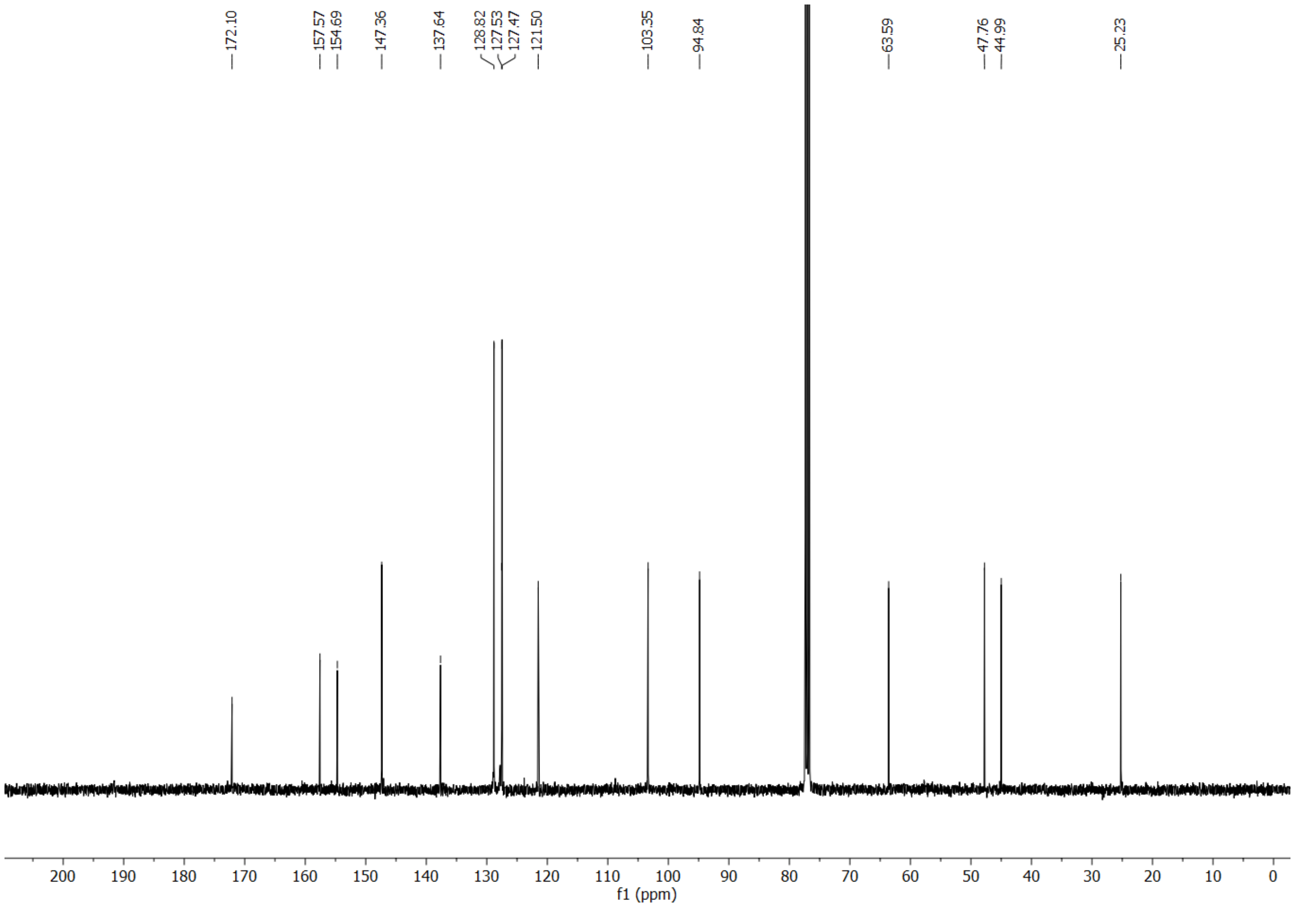
172.10 157.57 154.69 147.36 137.64 128.82 127.53 127.47 121.50 103.35 94.84 63.59 47.76 44.99 25.23
200 190 180 170 160 150 140 130 120 110 100 90 80 70 60 50 40 30 20 10 0
f1 (ppm)

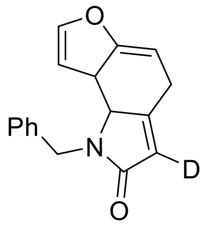
Ph
N
O
D
O

**2f**

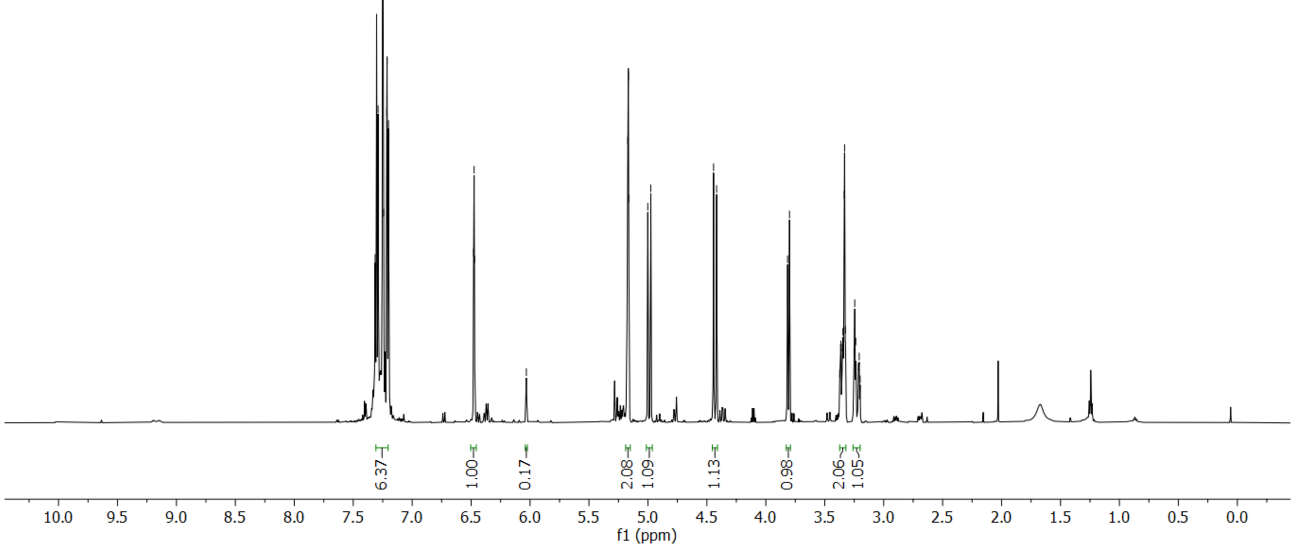
7.31
7.30
7.30
7.29
7.25
7.24
7.22
7.21
7.20
7.20
6.48
6.47
6.47
6.03
5.17
5.17
5.17
5.16
5.00
4.98
4.44
4.42
3.82
3.80
3.37
3.37
3.36
3.35
3.35
3.35
3.33
3.33
3.32
3.25
3.25
3.24
3.22
3.21
3.20
6.37
1.00
0.17
2.08
1.09
1.13
0.98
2.06
1.05
f1 (ppm)

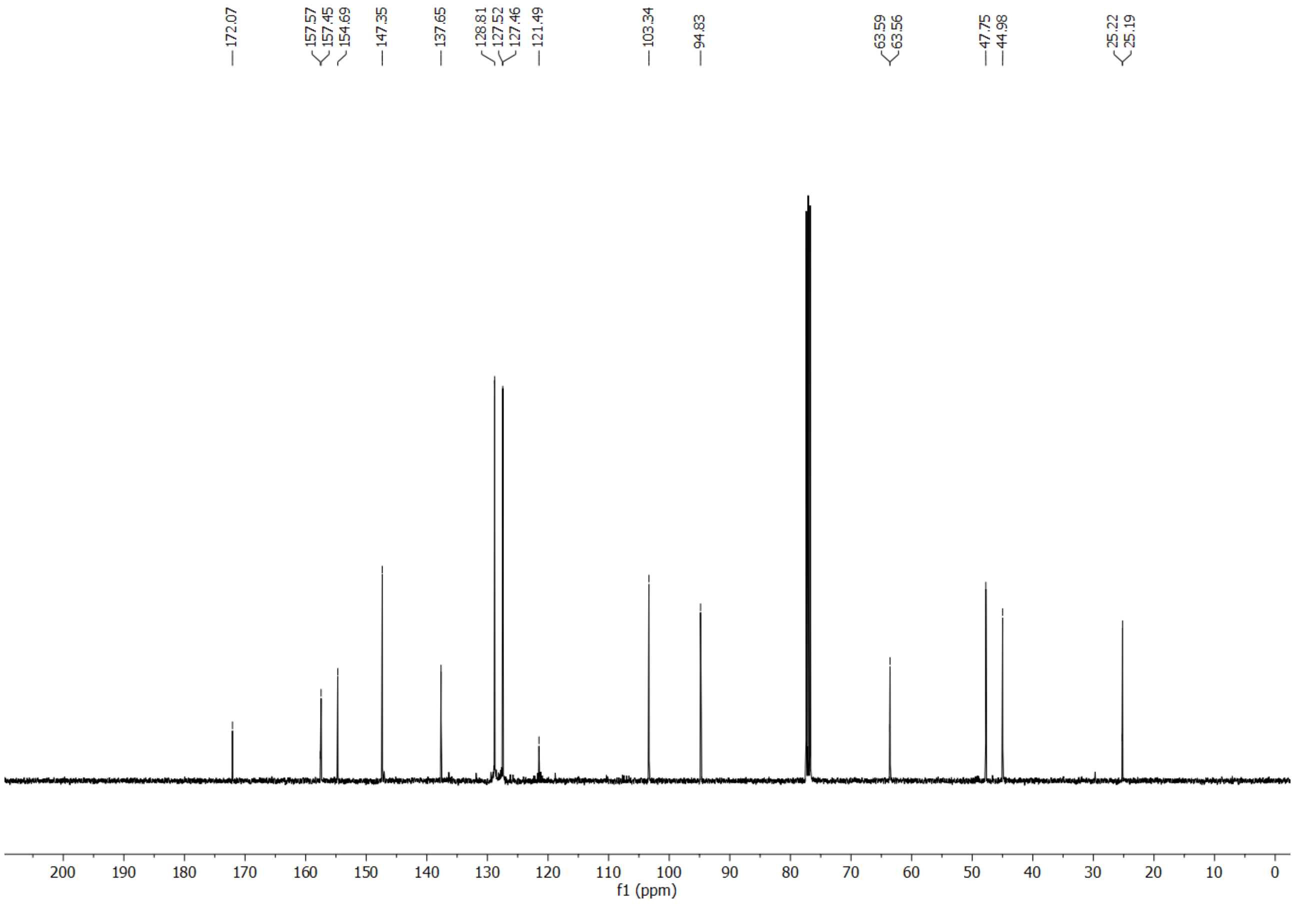
172.07
157.57
157.45
154.69
147.35
137.65
128.81
127.52
127.46
121.49
103.34
94.83
63.59
63.56
47.75
44.98
25.22
25.19
f1 (ppm)

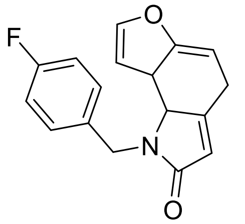

**2g**

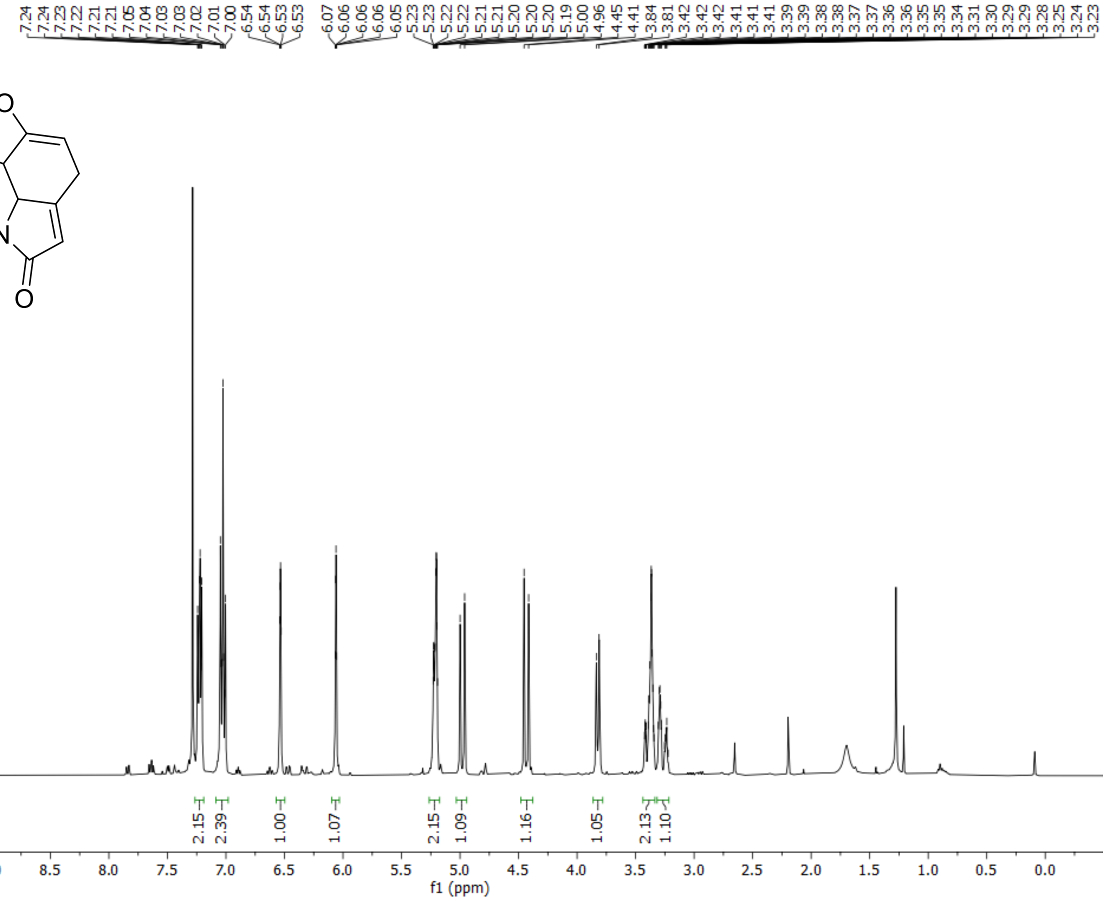
7.24 7.24 7.23 7.22 7.21 7.21 7.05 7.04 7.03 7.03 7.02 7.01 7.00 6.54 6.54 6.53 6.53 6.07 6.06 6.06 6.06 6.05 5.23 5.23 5.22 5.22 5.21 5.21 5.20 5.20 5.20 5.19 5.00 4.96 4.45 4.41 3.84 3.81 3.42 3.42 3.41 3.41 3.41 3.39 3.39 3.38 3.38 3.37 3.37 3.36 3.36 3.35 3.35 3.34 3.31 3.30 3.29 3.29 3.28 3.25 3.24 3.23
2.15 2.39 1.00 1.07 2.15 1.09 1.16 1.05 2.13 1.10
10.0 9.5 9.0 8.5 8.0 7.5 7.0 6.5 6.0 5.5 5.0 4.5 4.0 3.5 3.0 2.5 2.0 1.5 1.0 0.5 0.0
f1 (ppm)

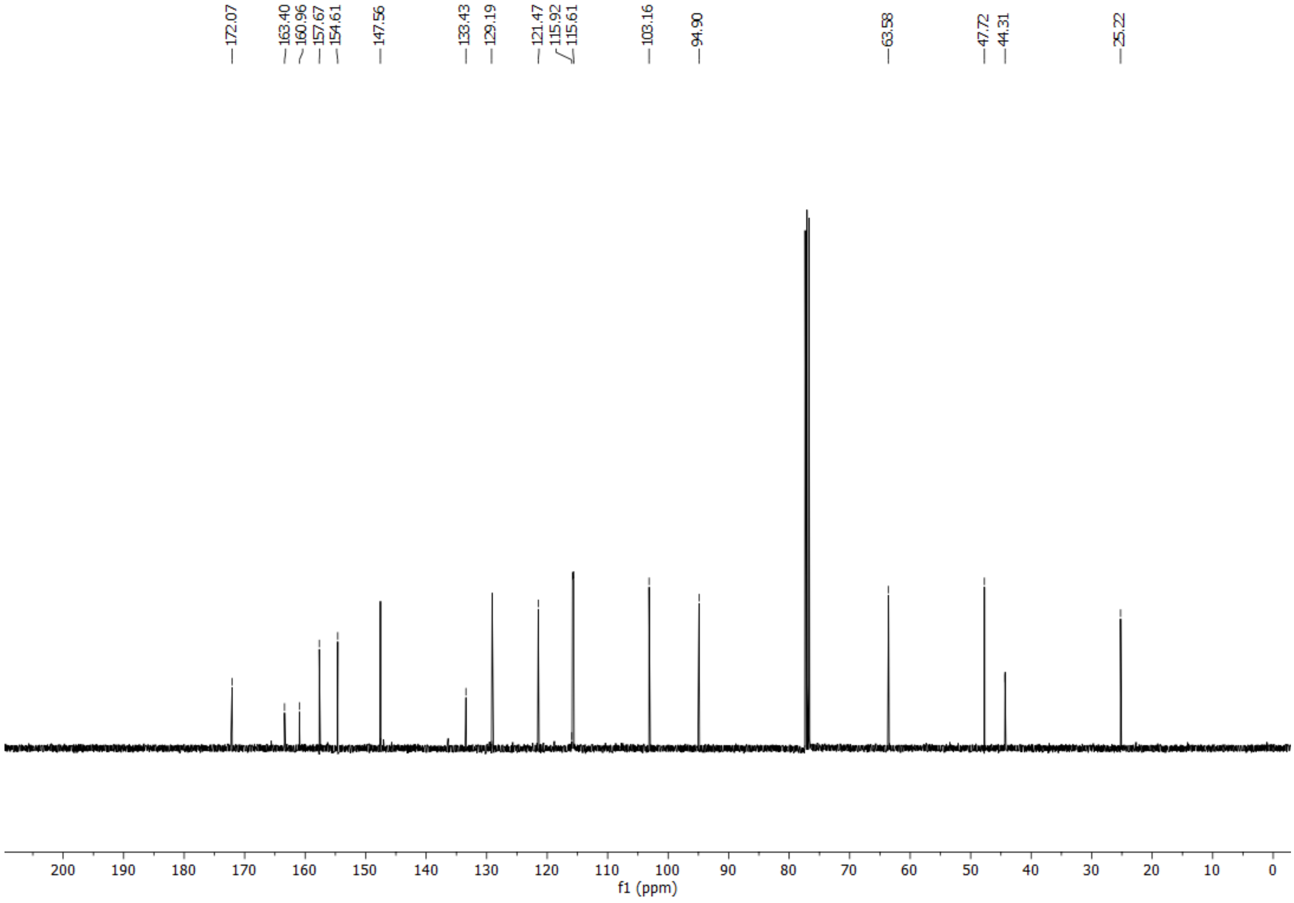
172.07 163.40 160.96 157.67 154.61 147.56 133.43 129.19 121.47 115.92 115.61 103.16 94.90 63.58 47.72 44.31 25.22
200 190 180 170 160 150 140 130 120 110 100 90 80 70 60 50 40 30 20 10 0
f1 (ppm)

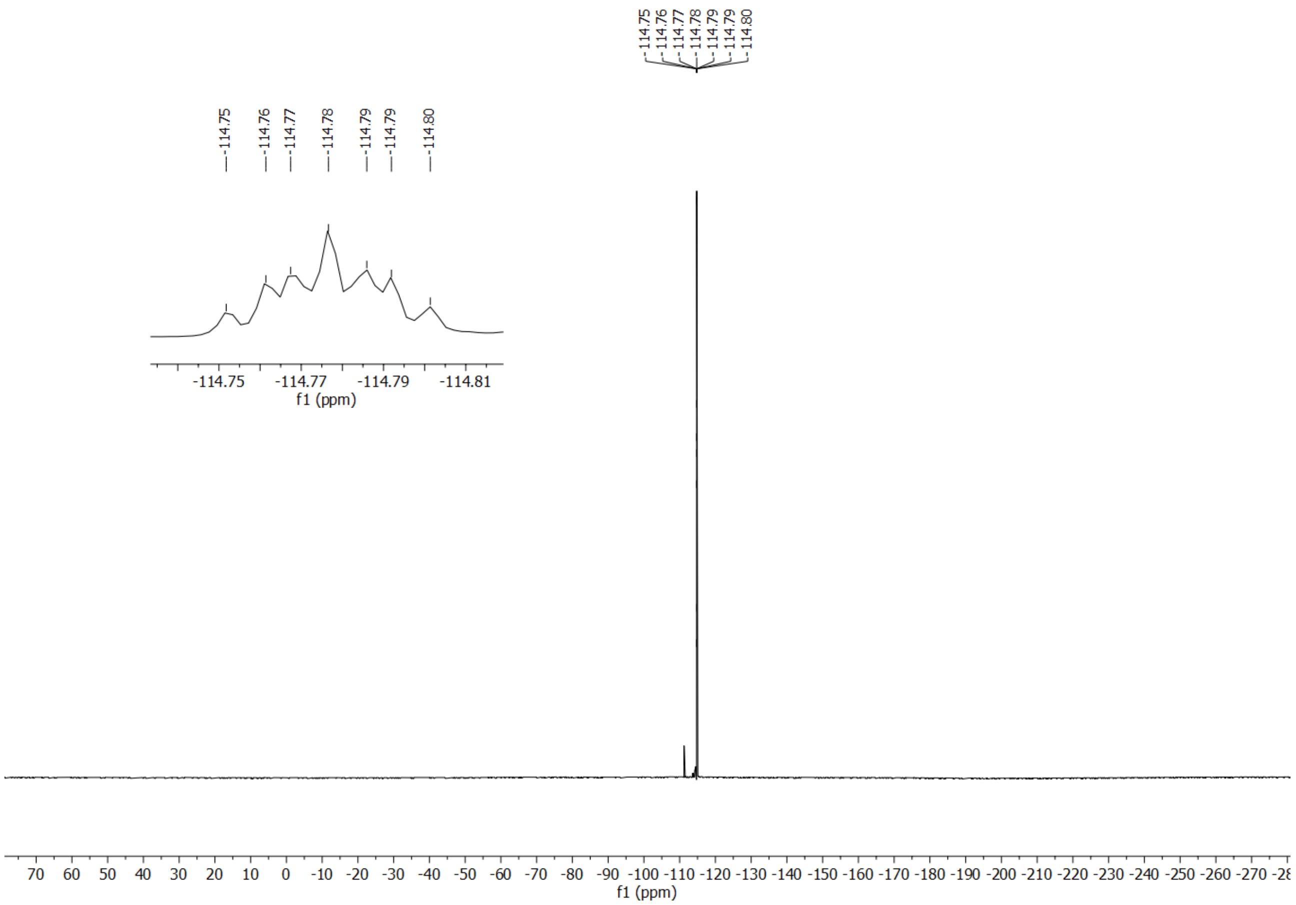
-114.75
-114.76
-114.77
-114.78
-114.79
-114.79
-114.80
-114.75
-114.77
-114.79
-114.81
f1 (ppm)
70 60 50 40 30 20 10 0 -10 -20 -30 -40 -50 -60 -70 -80 -90 -100 -110 -120 -130 -140 -150 -160 -170 -180 -190 -200 -210 -220 -230 -240 -250 -260 -270
f1 (ppm)

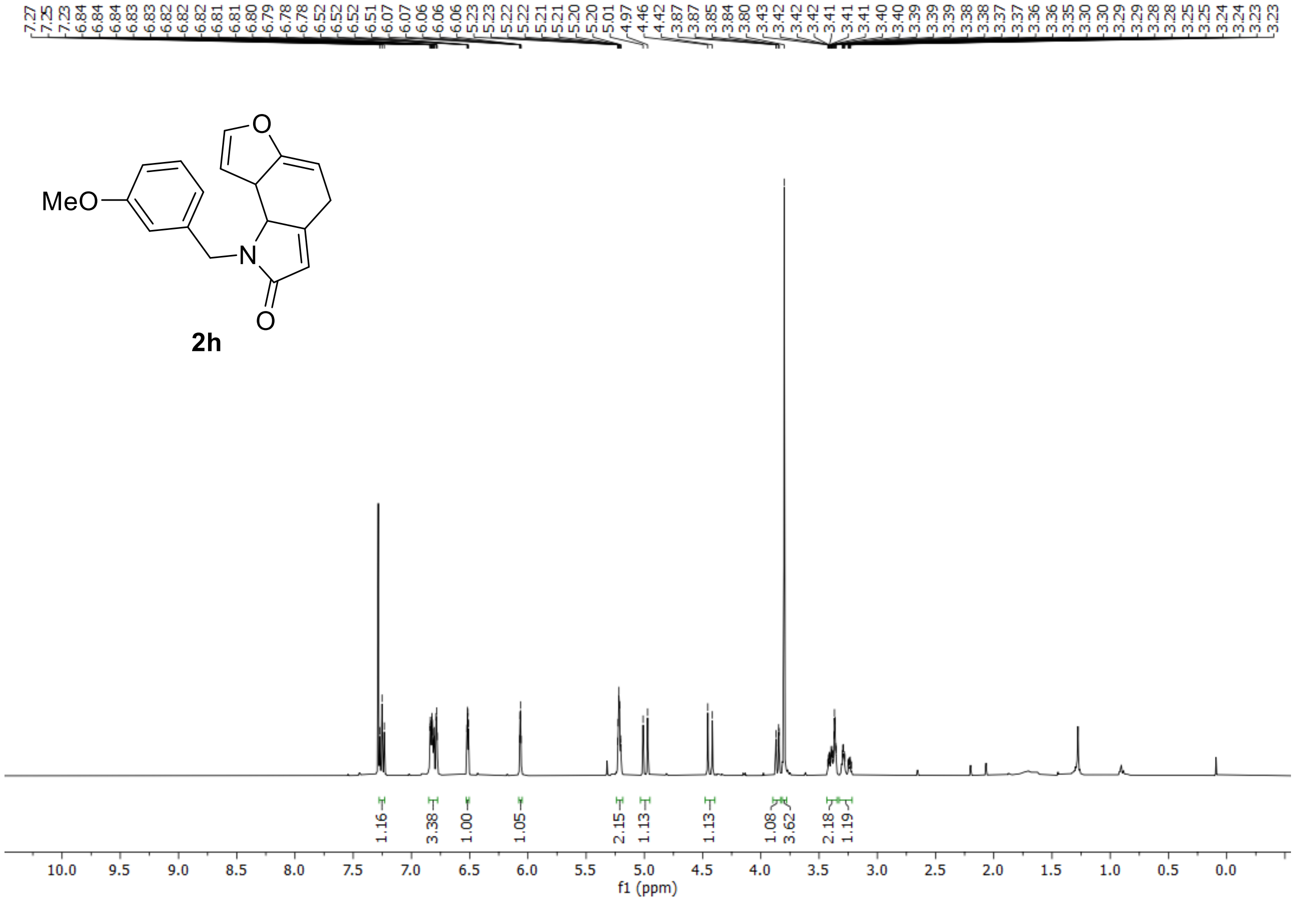
MeO
N
O
O
2h
7.27 7.25 7.23 6.84 6.84 6.83 6.83 6.82 6.82 6.82 6.81 6.81 6.80 6.79 6.78 6.78 6.52 6.52 6.52 6.51 6.07 6.07 6.06 6.06 5.23 5.23 5.22 5.22 5.21 5.21 5.20 5.20 5.01 4.97 4.46 4.42 3.87 3.87 3.85 3.84 3.80 3.43 3.42 3.42 3.41 3.41 3.41 3.40 3.40 3.39 3.39 3.39 3.38 3.38 3.37 3.37 3.36 3.36 3.35 3.30 3.29 3.29 3.28 3.28 3.25 3.25 3.24 3.24 3.23 3.23
1.16 3.38 1.00 1.05 2.15 1.13 1.13 1.08 3.62 2.18 1.19
f1 (ppm)

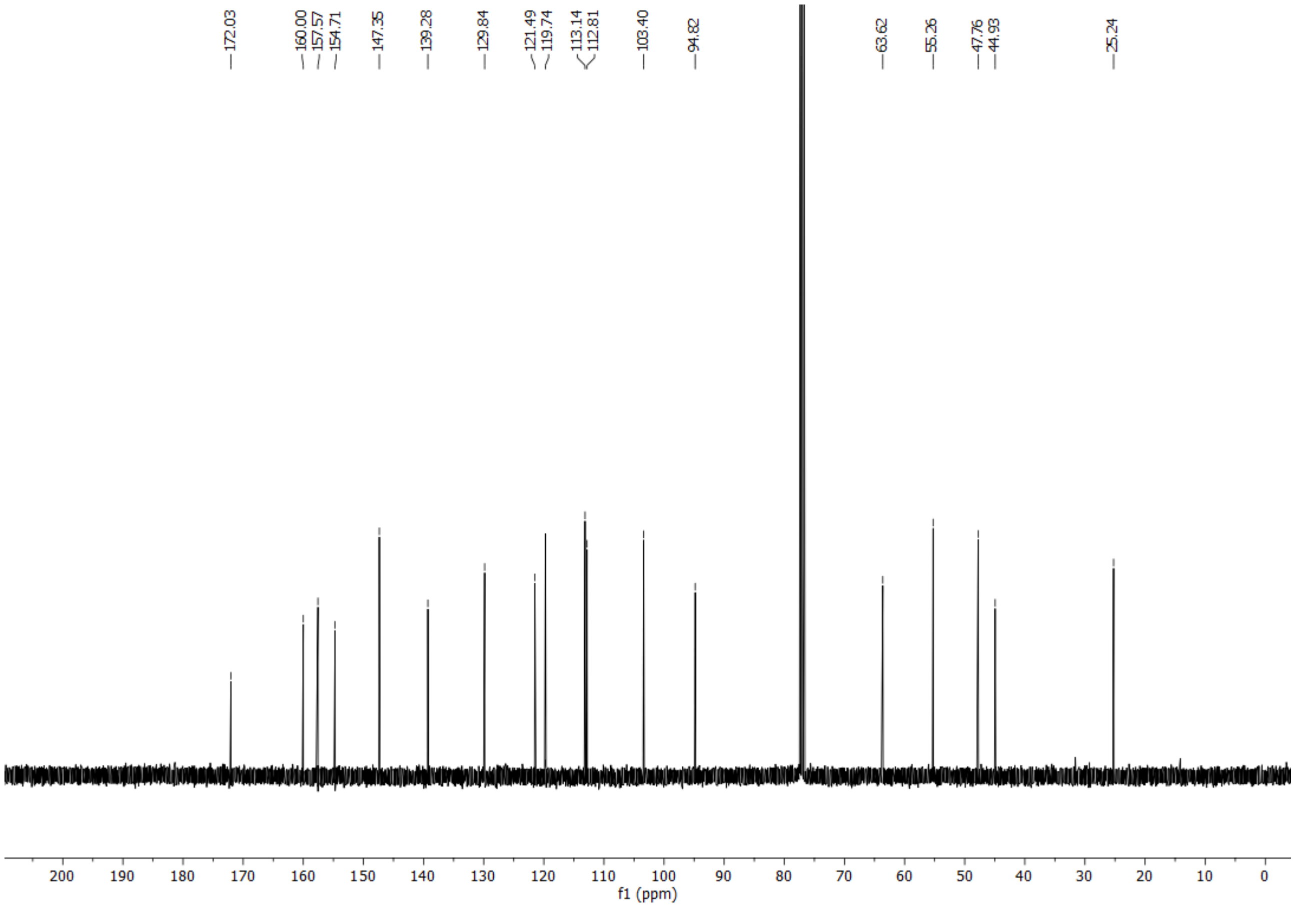
172.03 160.00 157.57 154.71 147.35 139.28 129.84 121.49 119.74 113.14 112.81 103.40 94.82 63.62 55.26 47.76 44.93 25.24
f1 (ppm)

**2i**

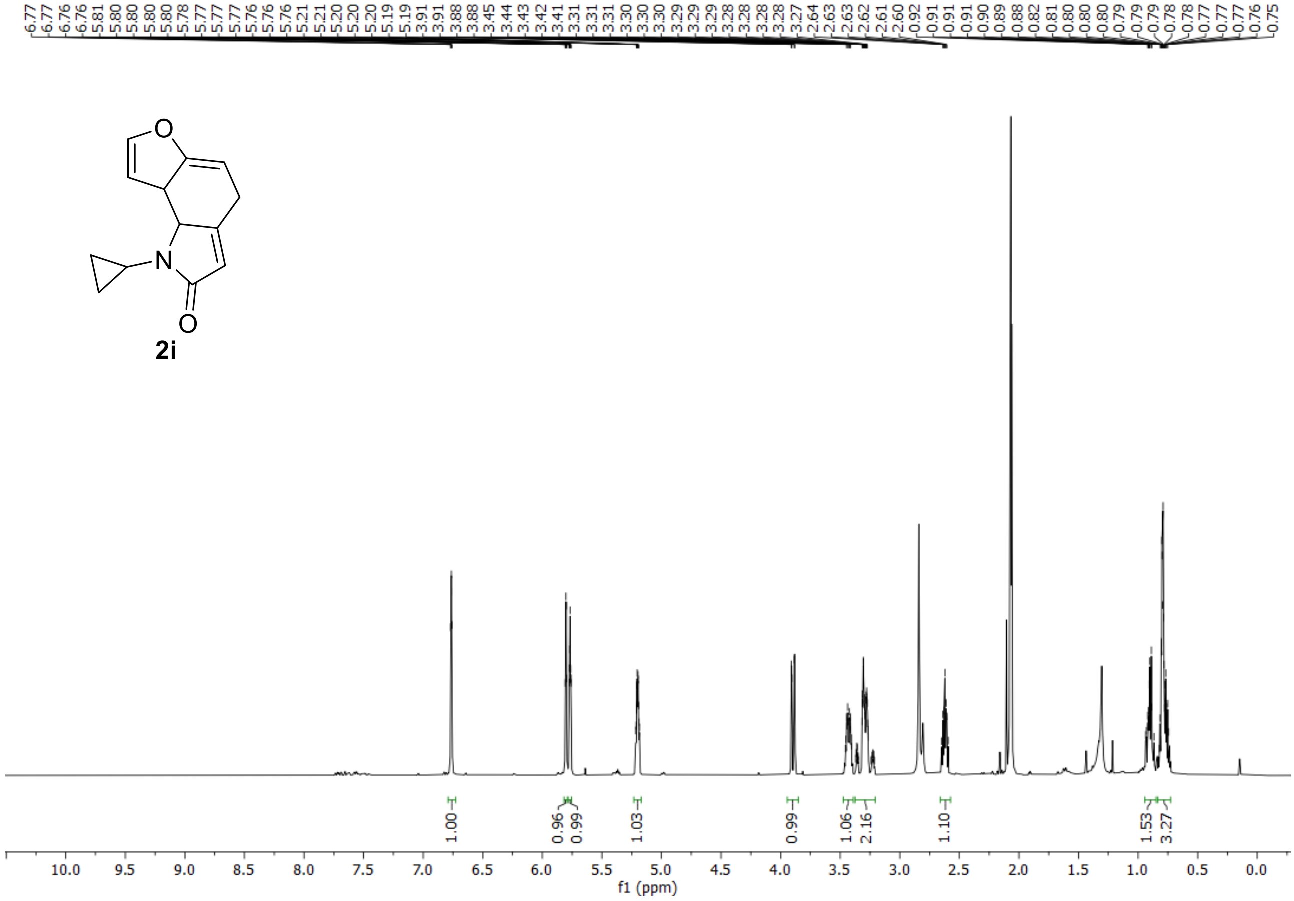

6.77 6.77 6.76 6.76 5.81 5.80 5.80 5.80 5.80 5.78 5.77 5.77 5.77 5.76 5.76 5.76 5.21 5.21 5.20 5.20 5.20 5.19 5.19 3.91 3.91 3.88 3.88 3.45 3.44 3.43 3.42 3.41 3.31 3.31 3.31 3.30 3.30 3.30 3.29 3.29 3.29 3.28 3.28 3.28 3.27 2.64 2.63 2.63 2.62 2.61 2.60 0.92 0.91 0.91 0.91 0.90 0.89 0.88 0.82 0.81 0.80 0.80 0.80 0.79 0.79 0.78 0.78 0.77 0.77 0.77 0.76 0.75
1.00 0.96 0.99 1.03 0.99 1.06 2.16 1.10 1.53 3.27
10.0 9.5 9.0 8.5 8.0 7.5 7.0 6.5 6.0 5.5 5.0 4.5 4.0 3.5 3.0 2.5 2.0 1.5 1.0 0.5 0.0
f1 (ppm)


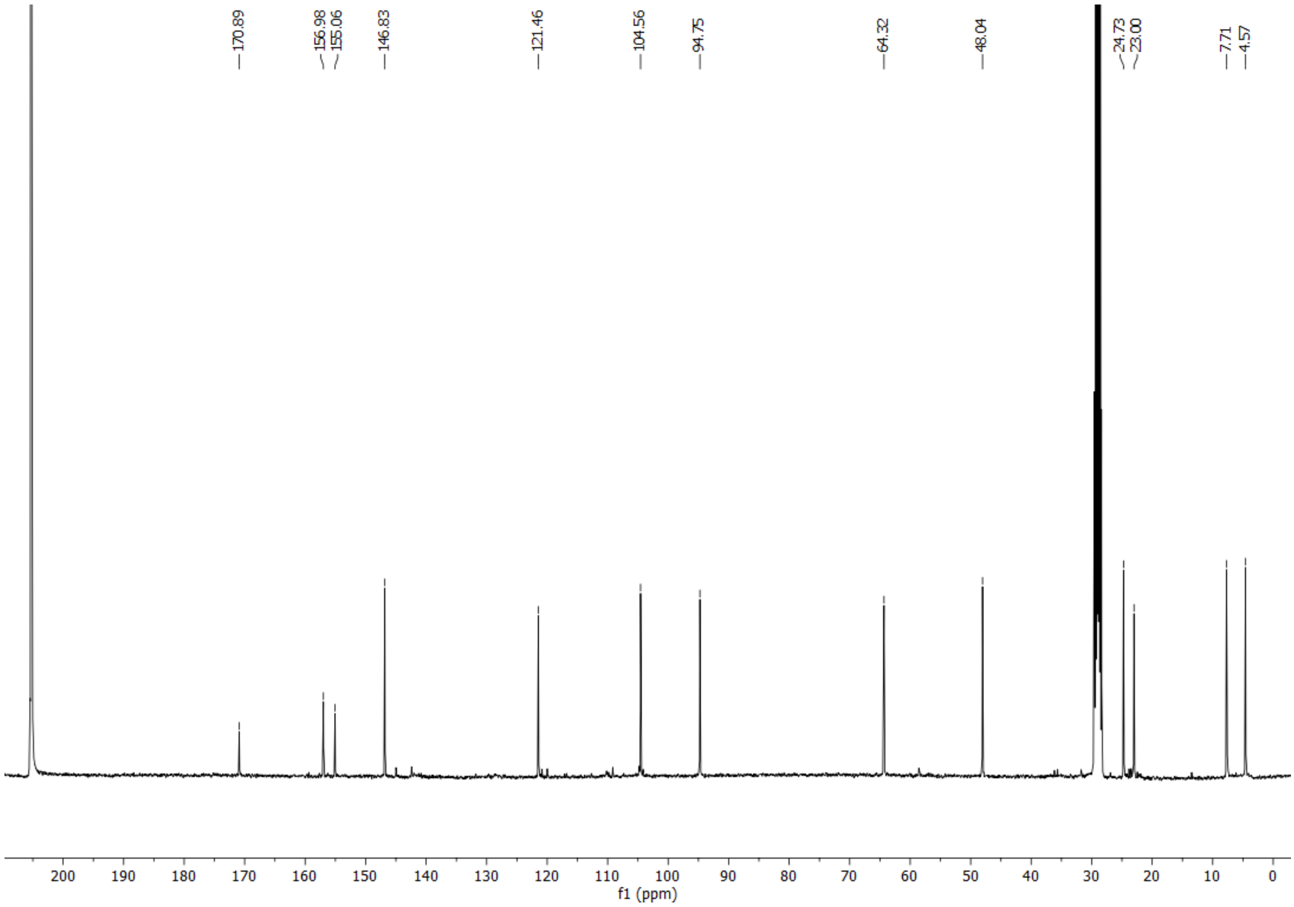

170.89 156.98 155.06 146.83 121.46 104.56 94.75 64.32 48.04 24.73 23.00 7.71 4.57
200 190 180 170 160 150 140 130 120 110 100 90 80 70 60 50 40 30 20 10 0
f1 (ppm)

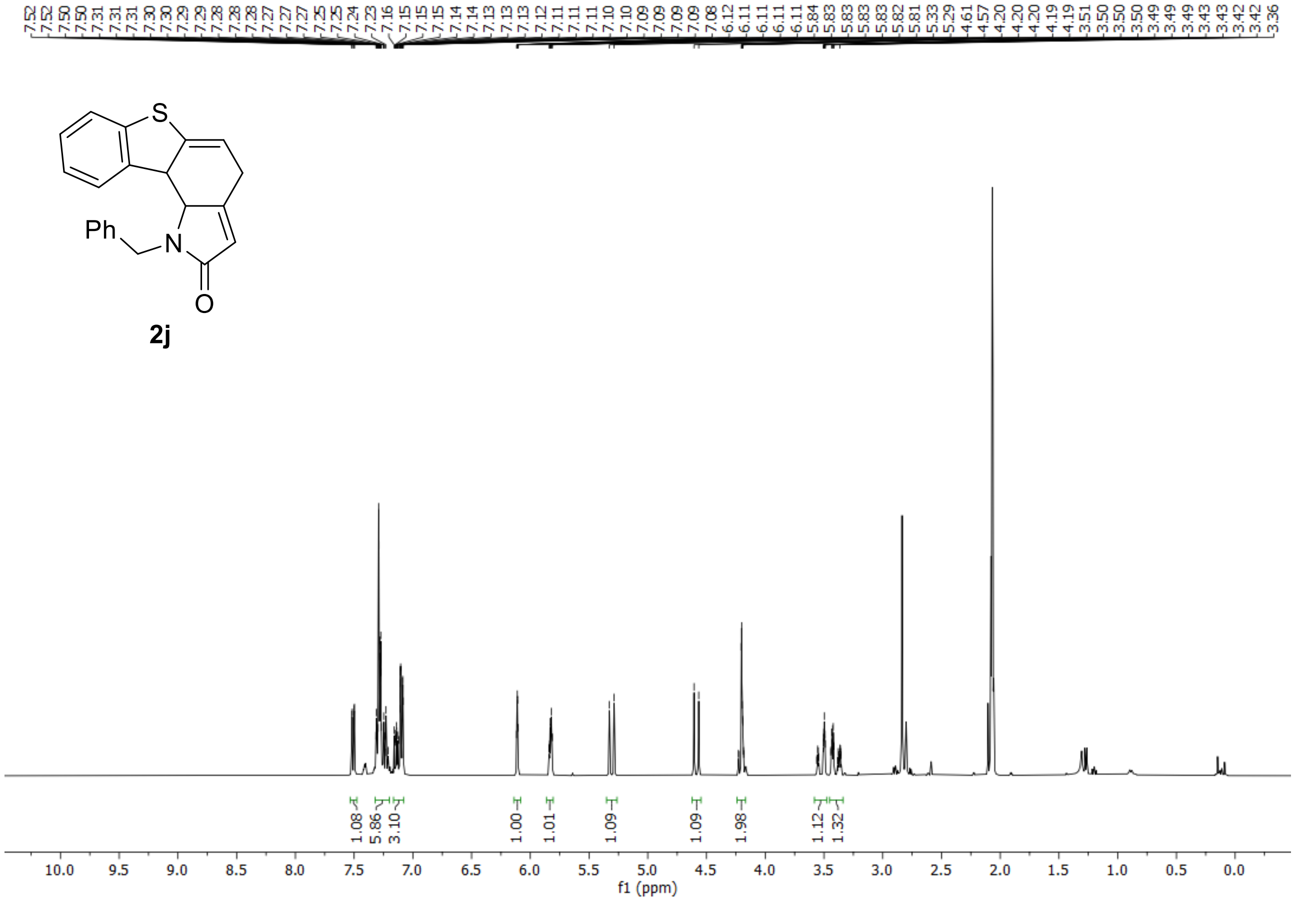

**2j**

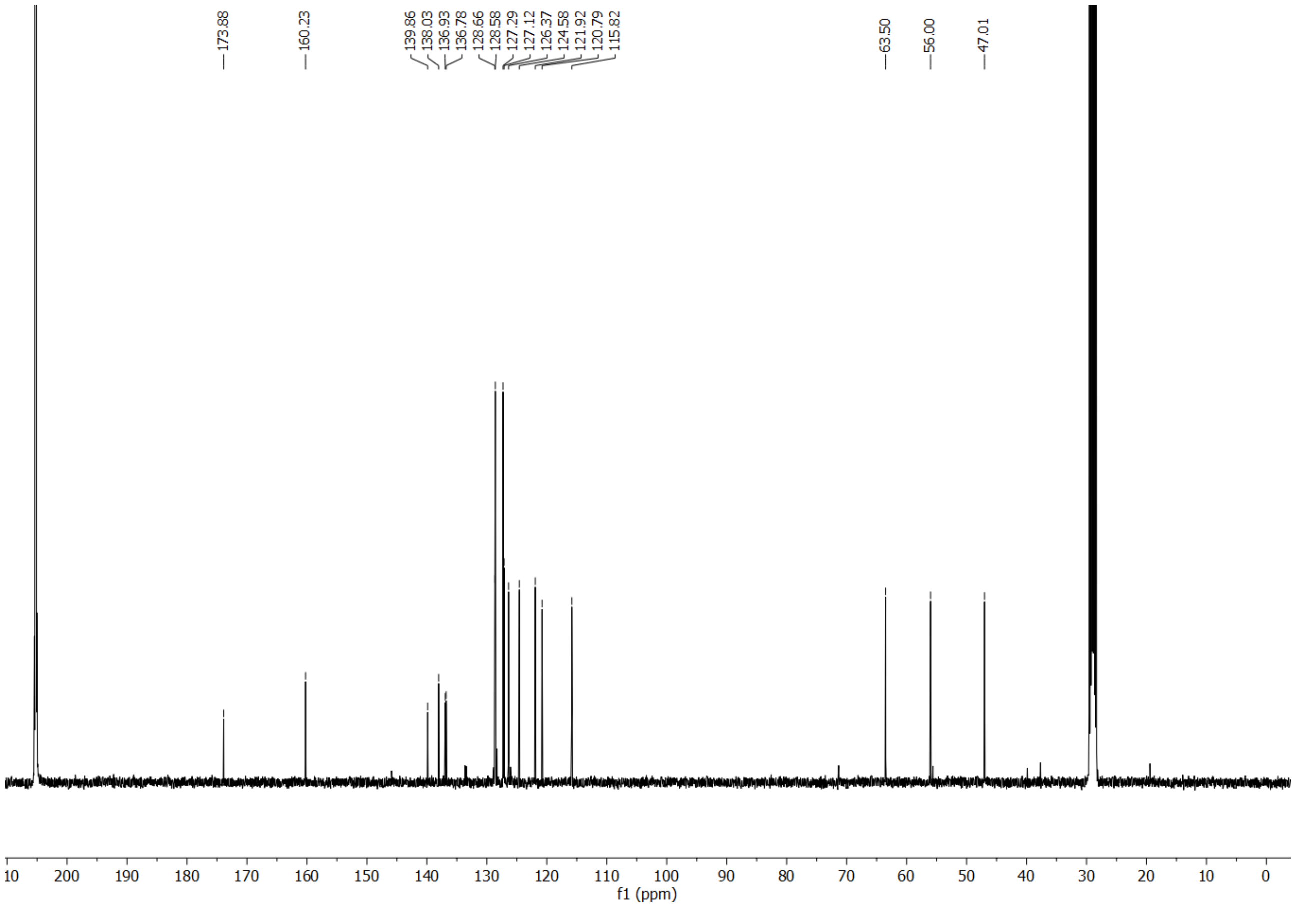

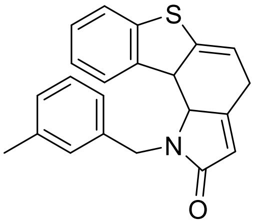
S
N
O

**2k**

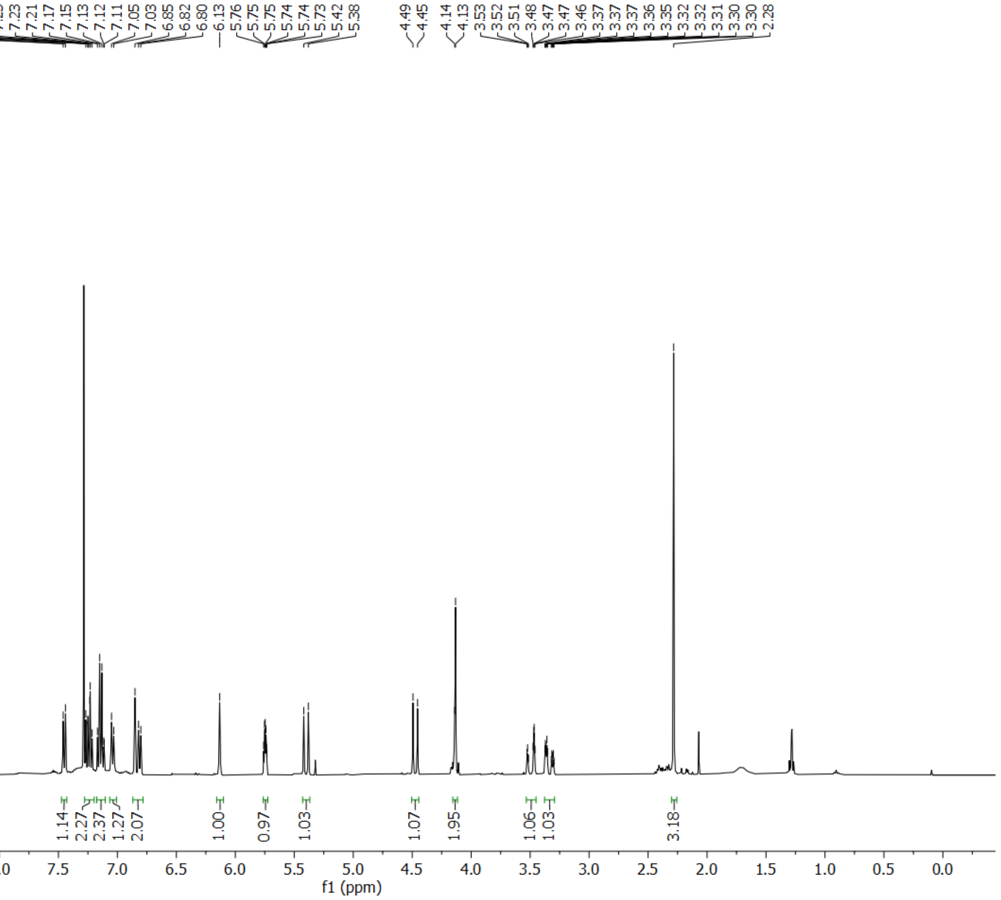
7.46
7.44
7.27
7.25
7.25
7.23
7.23
7.21
7.17
7.15
7.13
7.12
7.11
7.05
7.03
6.85
6.82
6.80
6.13
5.76
5.75
5.75
5.74
5.74
5.73
5.42
5.38
4.49
4.45
4.14
4.13
3.53
3.52
3.51
3.48
3.47
3.47
3.46
3.37
3.37
3.37
3.36
3.35
3.32
3.32
3.31
3.30
3.30
2.28
1.14
2.27
2.37
1.27
2.07
1.00
0.97
1.03
1.07
1.95
1.06
1.03
3.18
10.0
9.5
9.0
8.5
8.0
7.5
7.0
6.5
6.0
5.5
5.0
4.5
4.0
3.5
3.0
2.5
2.0
1.5
1.0
0.5
0.0
f1 (ppm)

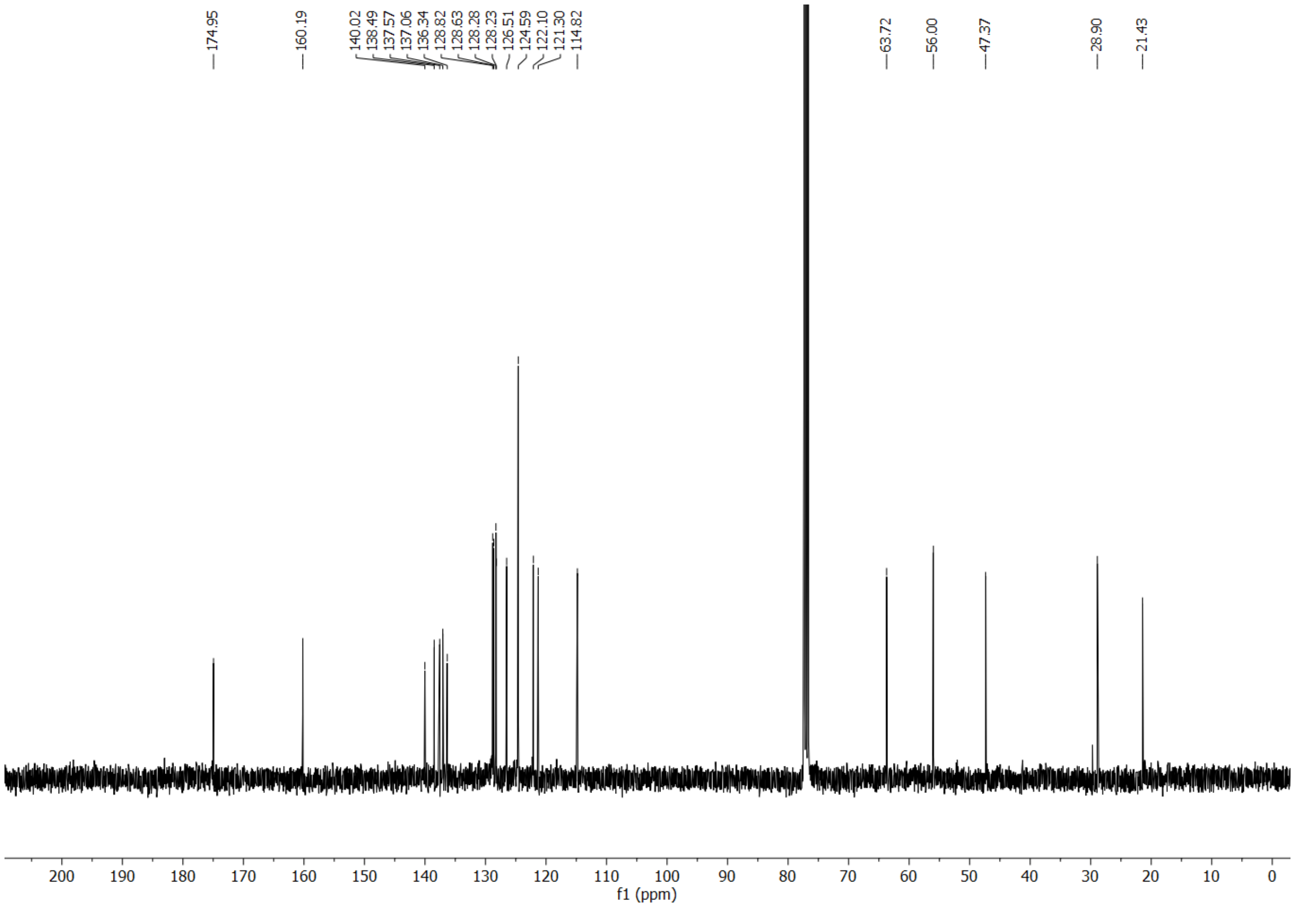
174.95
160.19
140.02
138.49
137.57
137.06
136.34
128.82
128.63
128.28
128.23
126.51
124.59
122.10
121.30
114.82
63.72
56.00
47.37
28.90
21.43
200
190
180
170
160
150
140
130
120
110
100
90
80
70
60
50
40
30
20
10
0
f1 (ppm)

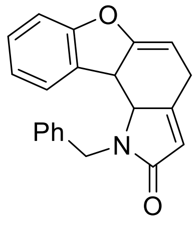

2l

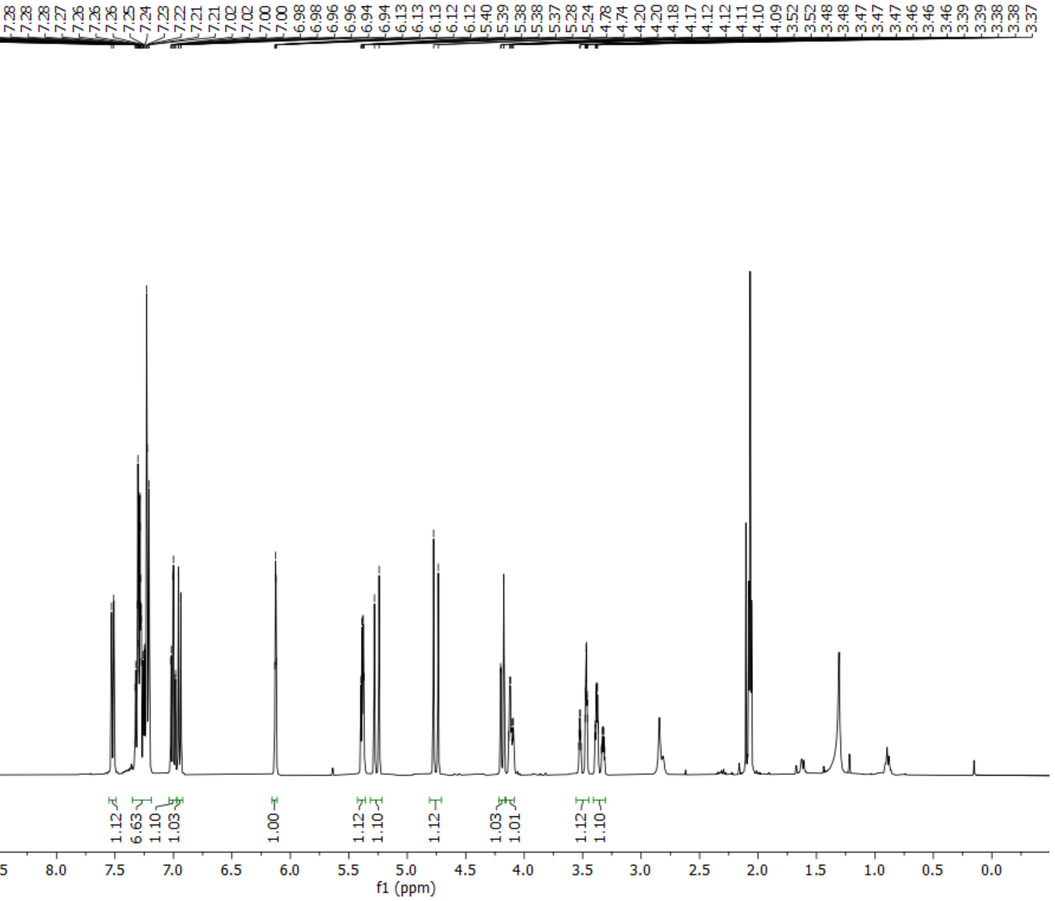
7.53
7.51
7.33
7.32
7.32
7.31
7.30
7.30
7.30
7.29
7.29
7.28
7.28
7.28
7.28
7.27
7.26
7.26
7.26
7.25
7.24
7.23
7.22
7.21
7.21
7.02
7.02
7.00
7.00
6.98
6.98
6.96
6.96
6.94
6.94
6.13
6.13
6.12
6.12
5.40
5.39
5.38
5.38
5.37
5.28
5.24
4.78
4.74
4.20
4.20
4.18
4.17
4.12
4.12
4.11
4.10
4.09
3.52
3.52
3.48
3.48
3.47
3.47
3.46
3.46
3.39
3.38
3.38
3.37
1.12
6.63
1.10
1.03
1.00
1.12
1.10
1.12
1.03
1.01
1.12
1.10
10.0 9.5 9.0 8.5 8.0 7.5 7.0 6.5 6.0 5.5 5.0 4.5 4.0 3.5 3.0 2.5 2.0 1.5 1.0 0.5 0.0
f1 (ppm)

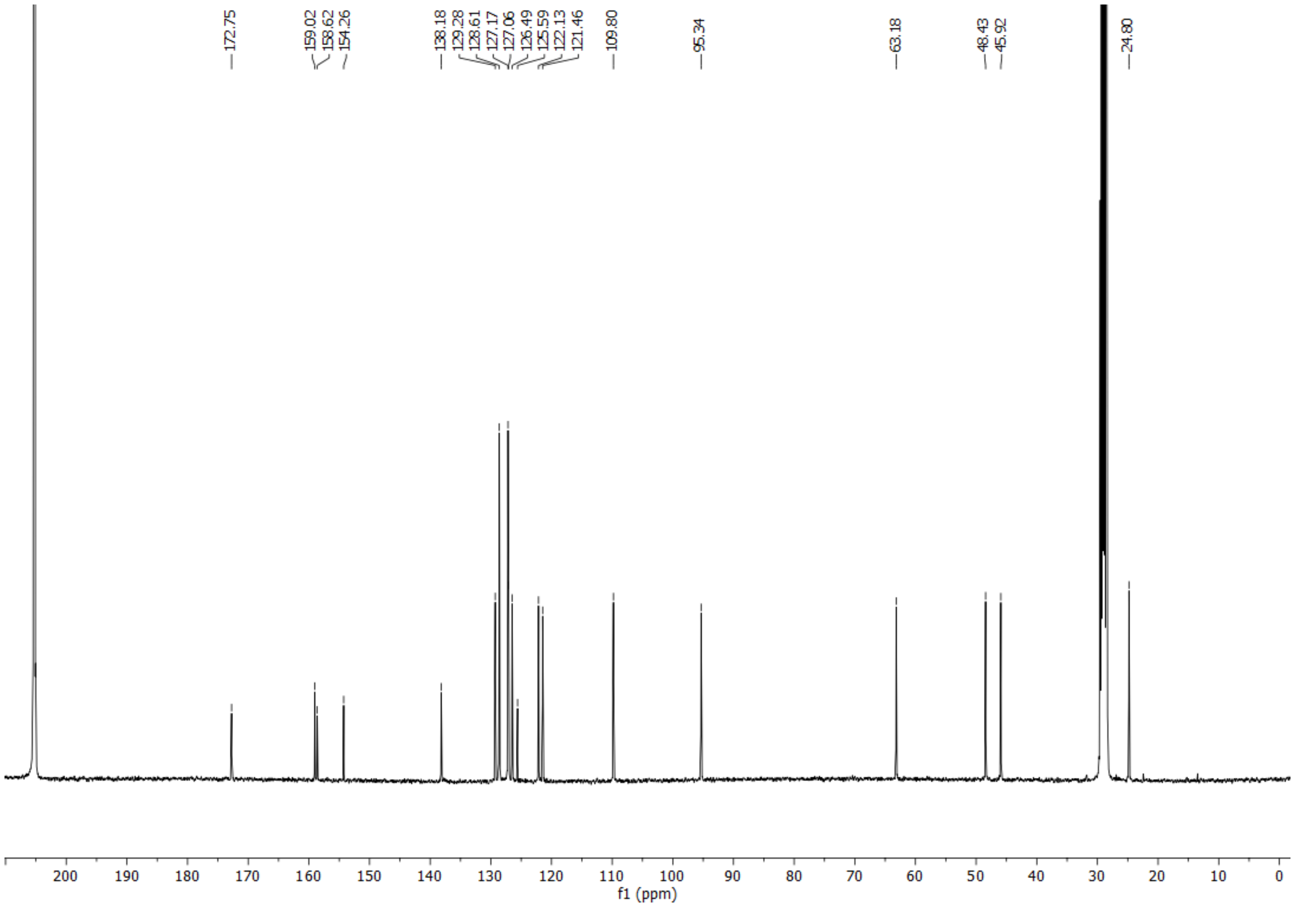
172.75
159.02
158.62
154.26
138.18
129.28
128.61
127.17
127.06
126.49
125.59
122.13
121.46
109.80
95.34
63.18
48.43
45.92
24.80
200 190 180 170 160 150 140 130 120 110 100 90 80 70 60 50 40 30 20 10 0
f1 (ppm)

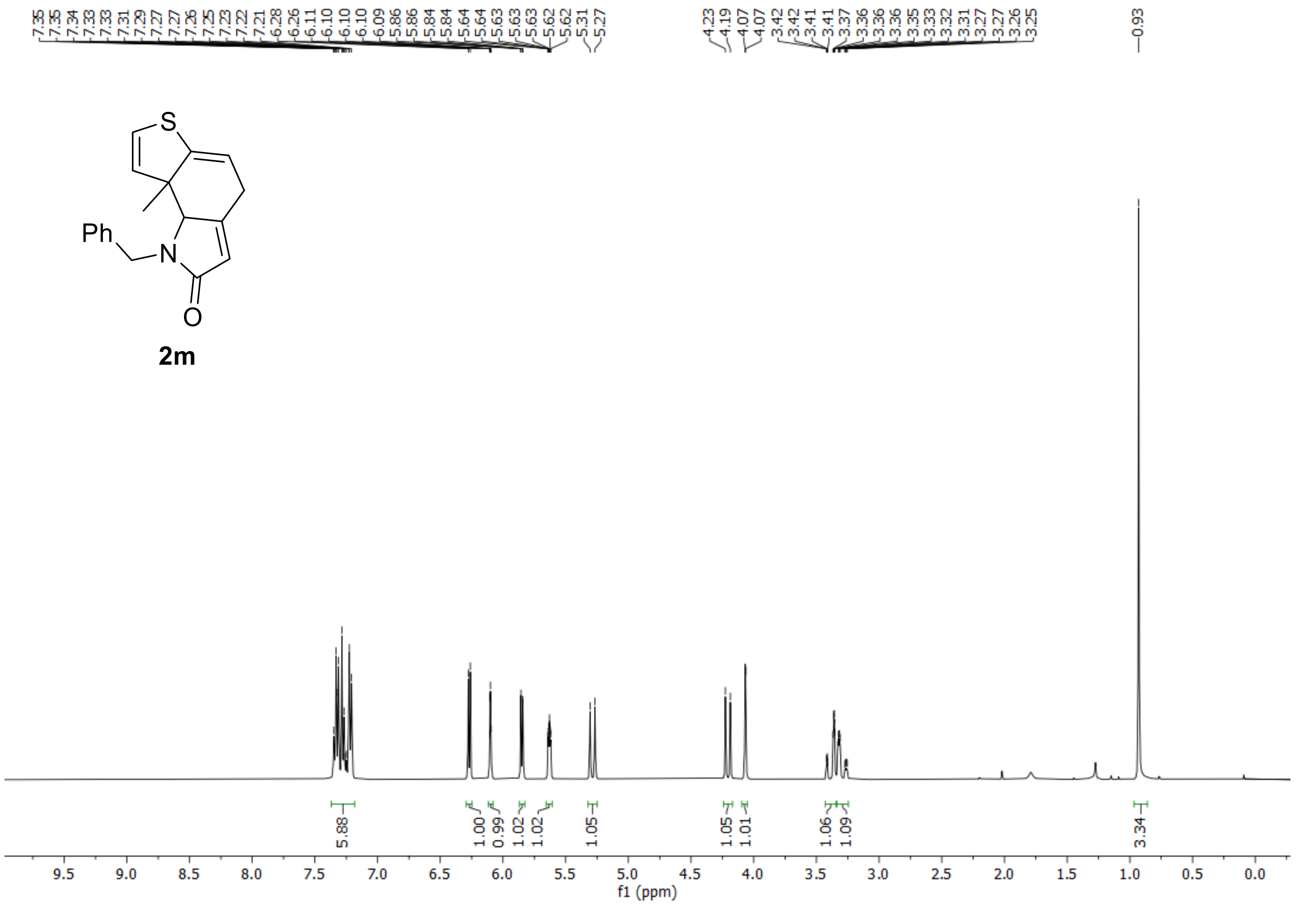

Ph
N
S
O
2m
f1 (ppm)


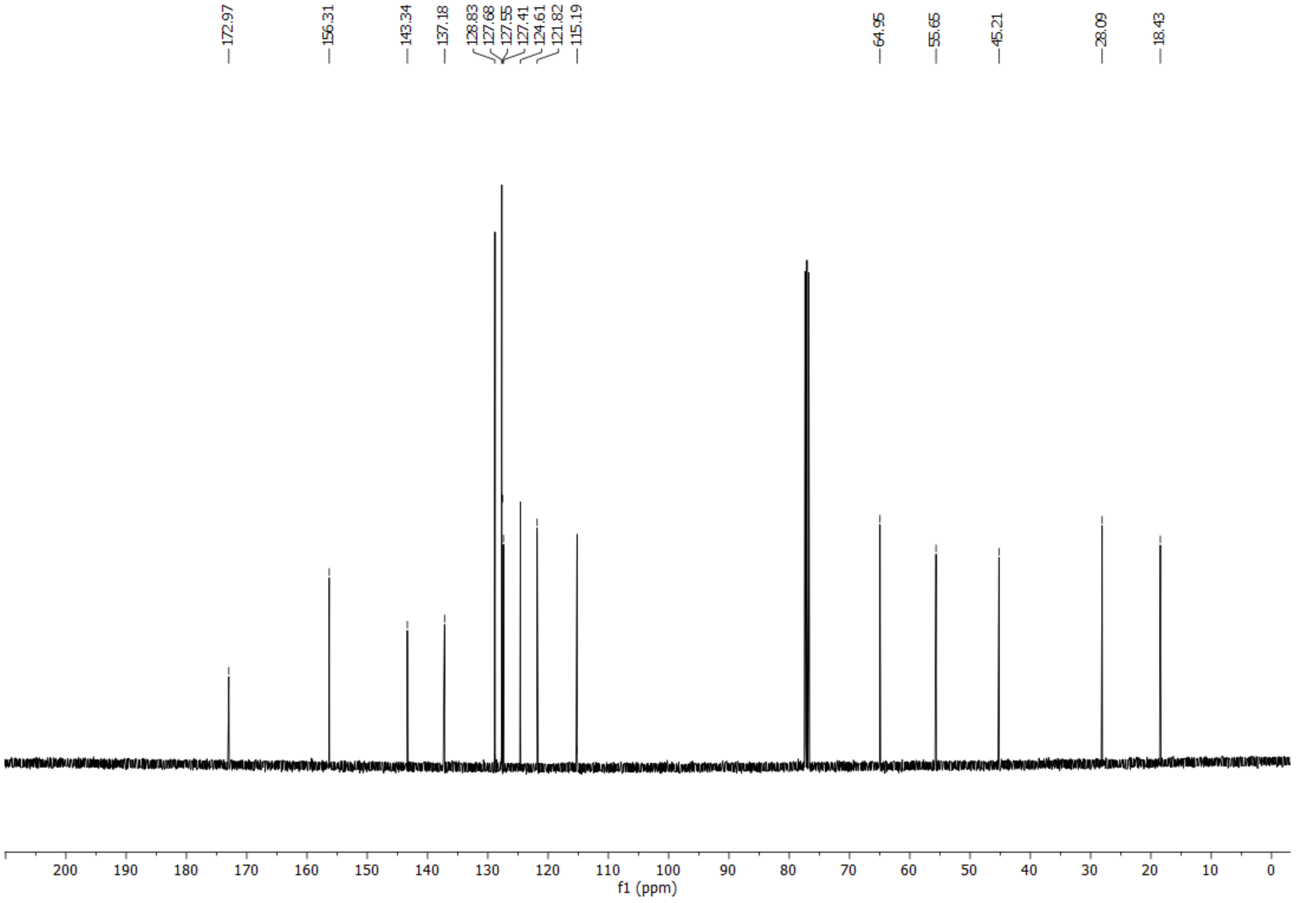

f1 (ppm)

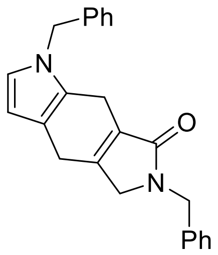

Ph
N
O
N
Ph


**2o**

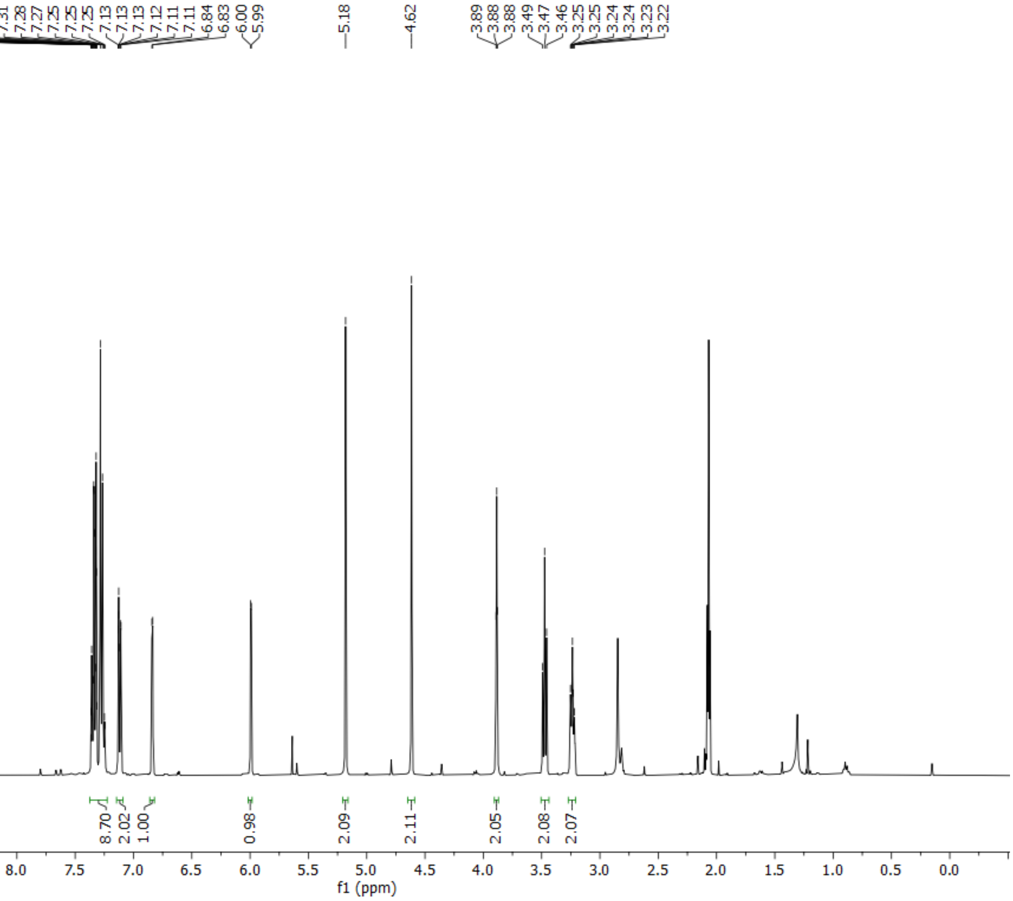

7.36 7.36 7.36 7.36 7.35 7.35 7.34 7.34 7.33 7.32 7.32 7.32 7.31 7.28 7.27 7.25 7.25 7.25 7.13 7.13 7.13 7.12 7.11 7.11 6.84 6.83 6.00 5.99
5.18
4.62
3.89 3.88 3.88 3.49 3.47 3.46 3.25 3.25 3.24 3.24 3.23 3.22
8.70 2.02 1.00 0.98 2.09 2.11 2.05 2.08 2.07
10.0 9.5 9.0 8.5 8.0 7.5 7.0 6.5 6.0 5.5 5.0 4.5 4.0 3.5 3.0 2.5 2.0 1.5 1.0 0.5 0.0
f1 (ppm)


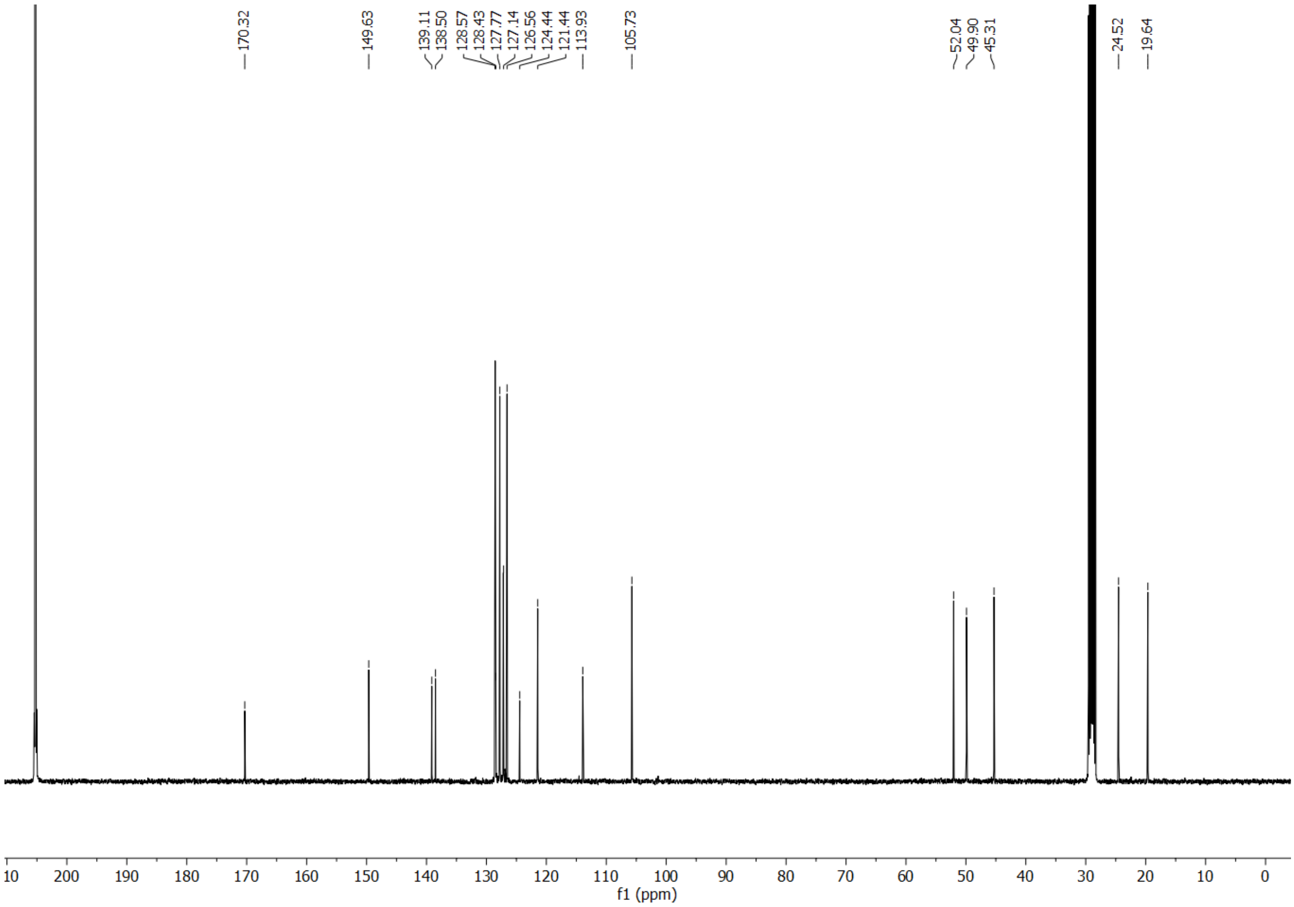

170.32
149.63
139.11 138.50 128.57 128.43 127.77 127.14 126.56 124.44 121.44 113.93
105.73
52.04 49.90 45.31
24.52 19.64
10 200 190 180 170 160 150 140 130 120 110 100 90 80 70 60 50 40 30 20 10 0
f1 (ppm)

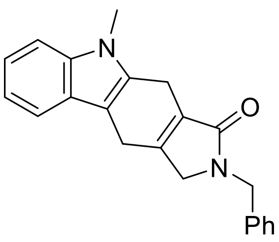
N
O
N
Ph

**2p**

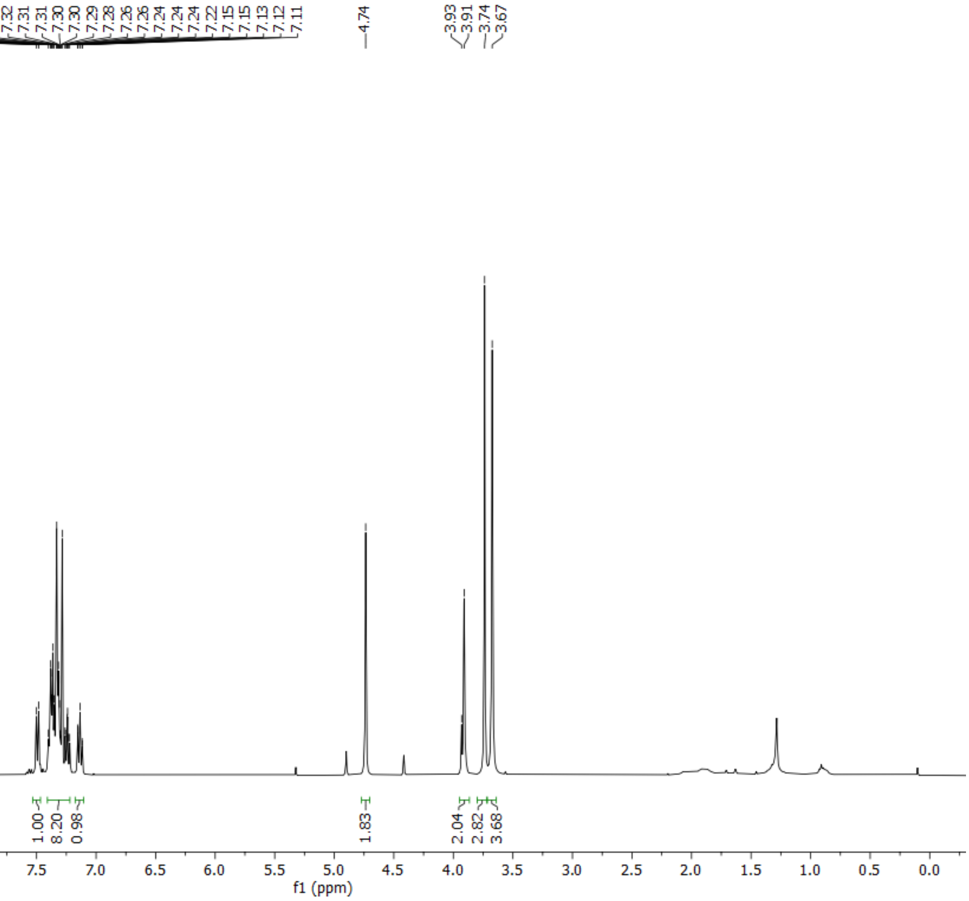
7.50 7.48 7.40 7.38 7.38 7.37 7.36 7.35 7.33 7.33 7.32 7.32 7.31 7.31 7.30 7.30 7.29 7.28 7.26 7.26 7.24 7.24 7.24 7.22 7.15 7.15 7.13 7.12 7.11
4.74
3.93 3.91 3.74 3.67
1.00 8.20 0.98
1.83
2.04 2.82 3.68
10.0 9.5 9.0 8.5 8.0 7.5 7.0 6.5 6.0 5.5 5.0 4.5 4.0 3.5 3.0 2.5 2.0 1.5 1.0 0.5 0.0
f1 (ppm)

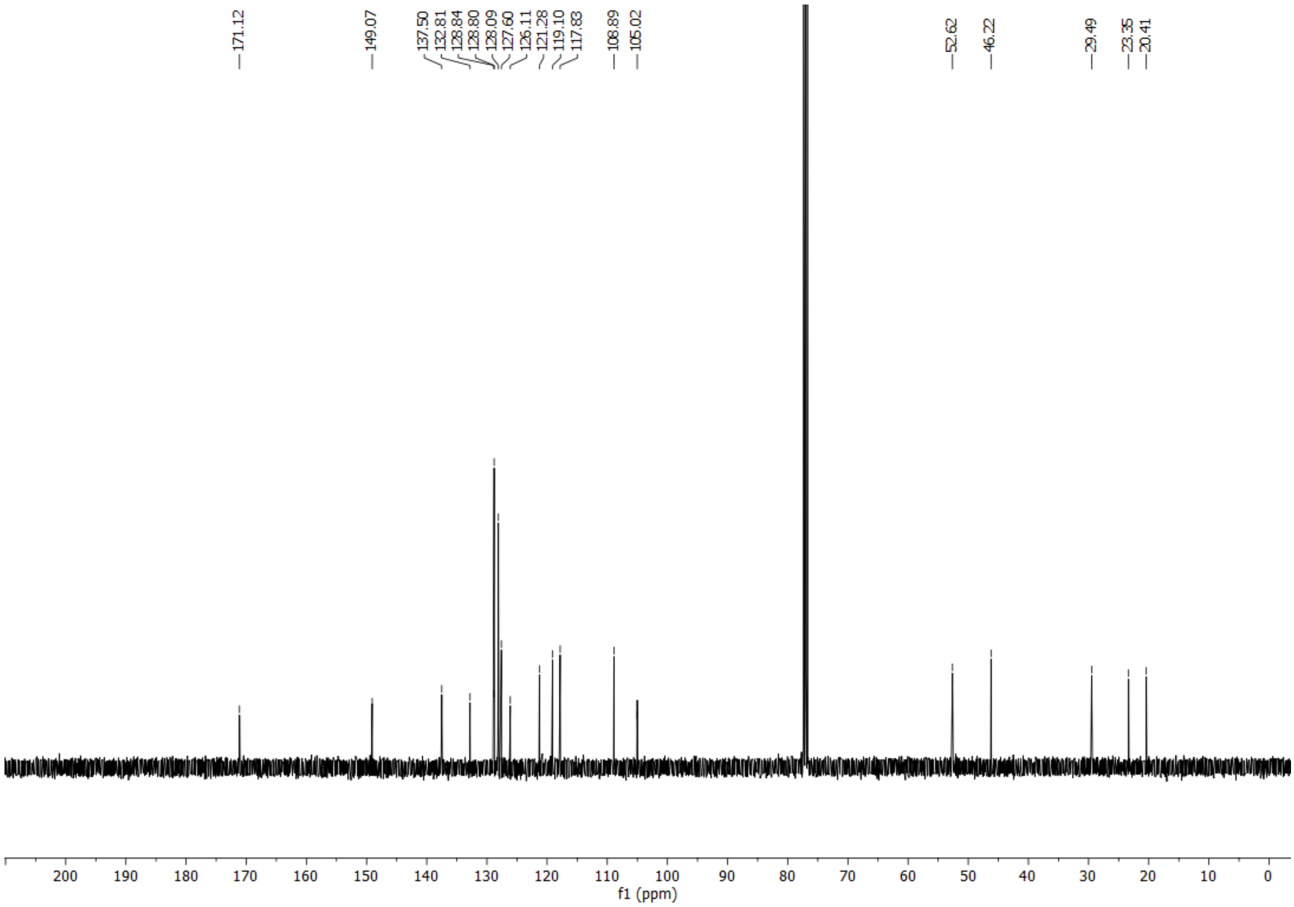
171.12
149.07
137.50 132.81 128.84 128.80 128.09 127.60 126.11 121.28 119.10 117.83
108.89
105.02
52.62
46.22
29.49
23.35
20.41
200 190 180 170 160 150 140 130 120 110 100 90 80 70 60 50 40 30 20 10 0
f1 (ppm)

# XYZ coordinates

**1**

25

scf done:  -953.688520

| | | | |
|---|---|---|---|
| C | -0.031689 | 0.016425 | 1.330438 |
| C | -0.048260 | 0.023400 | -0.106145 |
| C | 1.023718 | -0.015343 | -0.905169 |
| C | 0.842785 | -0.039781 | -2.366364 |
| N | 1.960477 | 0.146732 | -3.156051 |
| C | 3.200612 | 0.583944 | -2.680089 |
| C | 4.274103 | 0.725189 | -3.412050 |
| C | 5.365013 | 0.896533 | -4.091931 |
| O | -0.249218 | -0.241877 | -2.872698 |
| C | 1.846408 | -0.033461 | -4.588426 |
| H | 3.246321 | 0.859537 | -1.634423 |
| H | -1.023059 | 0.062440 | -0.587305 |
| H | 5.592286 | 1.838206 | -4.585340 |
| H | 6.101872 | 0.104961 | -4.202591 |
| H | 2.015691 | -0.069450 | -0.476729 |
| H | 2.591686 | -0.754991 | -4.930825 |
| H | 0.849999 | -0.394749 | -4.819710 |
| H | 2.022408 | 0.912049 | -5.107605 |
| S | -1.527200 | 0.073130 | 2.196926 |
| C | -0.731584 | 0.032531 | 3.702556 |
| C | 0.624959 | -0.021873 | 3.555412 |
| C | 1.023918 | -0.030735 | 2.208199 |
| H | 2.057680 | -0.069951 | 1.888731 |
| H | 1.311861 | -0.054259 | 4.390474 |
| H | -1.301884 | 0.050555 | 4.618799 |

**I**

25

scf done:  -953.619939

| | | | |
|---|---|---|---|
| C | 4.823938 | -1.591469 | -2.772904 |
| N | 3.688011 | -1.598682 | -1.866647 |
| C | 3.670028 | -0.680261 | -0.822440 |
| C | 2.422188 | -0.549724 | -0.060855 |
| C | 2.413897 | 0.135297 | 1.089281 |
| C | 1.266644 | 0.383472 | 1.915981 |
| C | 2.742437 | -2.585476 | -2.056529 |
| C | 2.780408 | -3.442793 | -3.113462 |
| C | 2.173131 | -4.487861 | -3.685225 |
| O | 4.654672 | -0.001042 | -0.583260 |
| H | 4.465014 | -1.565581 | -3.803609 |
| H | 1.979998 | -2.684761 | -1.290064 |
| H | 1.275072 | -4.933965 | -3.256525 |
| H | 2.545562 | -4.937688 | -4.599859 |
| H | 5.433441 | -0.717924 | -2.566996 |
| H | 5.424104 | -2.494614 | -2.640777 |
| H | 1.505210 | -0.971507 | -0.451688 |
| H | 3.363131 | 0.542623 | 1.430322 |

| | | | |
|---|---|---|---|
| S | 1.457049 | 1.263951 | 3.392511 |
| C | -0.203381 | 1.146010 | 3.752474 |
| C | -0.883036 | 0.461912 | 2.785312 |
| C | -0.049075 | 0.027925 | 1.741510 |
| H | -0.402098 | -0.529025 | 0.882834 |
| H | -1.948360 | 0.278550 | 2.825317 |
| H | -0.595557 | 1.586255 | 4.656632 |

**I' (triplet localized on the former alkenyl arm)**

25

scf done: -953.610355

| | | | |
|---|---|---|---|
| C | 0.980426 | -0.089850 | 2.115143 |
| C | -0.138143 | 0.015564 | 1.276210 |
| S | -1.594400 | 0.094789 | 2.247675 |
| C | -0.691978 | -0.012203 | 3.705304 |
| C | 0.655434 | -0.103684 | 3.471774 |
| C | -0.170133 | 0.060603 | -0.103388 |
| C | 1.041225 | 0.033329 | -0.902783 |
| C | 1.668734 | -1.233424 | -1.261514 |
| O | 1.322325 | -2.278429 | -0.727668 |
| N | 2.696351 | -1.210141 | -2.187768 |
| C | 3.468072 | -2.414592 | -2.410061 |
| C | 3.004911 | -0.106945 | -2.987592 |
| C | 4.018397 | -0.041136 | -3.810504 |
| C | 4.997512 | 0.070217 | -4.653051 |
| H | 2.316173 | 0.727774 | -2.940732 |
| H | -1.127049 | 0.142052 | -0.614930 |
| H | 4.902423 | -0.245054 | -5.689088 |
| H | 5.960465 | 0.481741 | -4.361326 |
| H | 1.514400 | 0.974829 | -1.165908 |
| H | 4.528024 | -2.215463 | -2.234759 |
| H | 3.120856 | -3.184717 | -1.729145 |
| H | 3.350449 | -2.756431 | -3.441345 |
| H | 1.986286 | -0.160695 | 1.718709 |
| H | 1.384599 | -0.182020 | 4.267537 |
| H | -1.201304 | -0.005178 | 4.657037 |

**TS (I-II)**

25

scf done: -953.616783

| | | | |
|---|---|---|---|
| C | 0.032887 | -0.054166 | 0.052368 |
| C | 0.088326 | 0.229656 | 1.398228 |
| S | 1.737138 | 0.355908 | 1.912233 |
| C | 2.321086 | 0.041398 | 0.338290 |
| C | 1.300833 | -0.157492 | -0.545987 |
| C | -0.971881 | 0.409707 | 2.342216 |
| C | -2.288572 | 0.390816 | 2.034916 |
| C | -3.330253 | -1.727596 | 1.688263 |
| C | -4.478949 | -1.235207 | 2.230234 |
| N | -4.475213 | -0.051246 | 2.942651 |
| C | -3.313287 | 0.689169 | 3.054241 |
| C | -2.762405 | -2.685815 | 0.954214 |
| O | -3.181144 | 1.524749 | 3.929042 |

| | | | |
|---|---|---|---|
| C | -5.601297 | 0.296424 | 3.785931 |
| H | -5.441169 | -0.033322 | 4.815018 |
| H | -5.442681 | -1.736485 | 2.162441 |
| H | -3.331311 | -3.524735 | 0.552847 |
| H | -1.696612 | -2.674409 | 0.742051 |
| H | -5.740003 | 1.375653 | 3.789407 |
| H | -6.496722 | -0.182264 | 3.394094 |
| H | -2.614423 | 0.380829 | 0.999495 |
| H | -0.695073 | 0.533889 | 3.386347 |
| H | -0.899051 | -0.194755 | -0.481696 |
| H | 1.458733 | -0.375402 | -1.593930 |
| H | 3.383260 | 0.019196 | 0.148054 |

**II**

25

scf done: -953.682542

| | | | |
|---|---|---|---|
| C | 0.030267 | 0.090538 | -0.002730 |
| C | -0.017259 | -0.027473 | 1.395199 |
| S | 1.619543 | -0.087767 | 2.026154 |
| C | 2.295501 | 0.047286 | 0.453277 |
| C | 1.328652 | 0.132222 | -0.513368 |
| C | -1.104649 | -0.097432 | 2.240768 |
| C | -2.514085 | -0.079606 | 1.772129 |
| C | -3.186901 | -1.442071 | 1.736528 |
| C | -4.356089 | -1.325911 | 2.480412 |
| N | -4.482796 | -0.054732 | 3.015581 |
| C | -3.419066 | 0.745035 | 2.680103 |
| C | -2.697592 | -2.535205 | 1.090049 |
| O | -3.249175 | 1.886023 | 3.043596 |
| C | -5.575337 | 0.370091 | 3.851659 |
| H | -5.635491 | -0.247639 | 4.749787 |
| H | -5.111299 | -2.073301 | 2.676355 |
| H | -3.232316 | -3.477171 | 1.099904 |
| H | -1.755798 | -2.492338 | 0.556491 |
| H | -5.401705 | 1.404859 | 4.139844 |
| H | -6.522007 | 0.300827 | 3.312644 |
| H | -2.575580 | 0.380193 | 0.778457 |
| H | -0.938877 | -0.218239 | 3.307335 |
| H | -0.860954 | 0.137415 | -0.615616 |
| H | 1.558192 | 0.221351 | -1.567200 |
| H | 3.367037 | 0.057545 | 0.323613 |

**[1] III**

25

scf done: -953.706144

| | | | |
|---|---|---|---|
| N | -3.716569 | -0.194642 | -0.148865 |
| C | -3.903894 | -1.292616 | 0.708279 |
| C | -3.335534 | -1.053352 | 1.889426 |
| C | -2.525982 | 0.196742 | 1.769497 |
| C | -2.817184 | 0.710034 | 0.380954 |
| C | -4.348524 | -0.040860 | -1.431370 |
| C | -2.346072 | -1.745487 | 2.775655 |
| C | -1.319614 | -0.734156 | 2.175134 |

| | | | |
|---|---|---|---|
| C | -0.270394 | -0.164181 | 3.055046 |
| C | -0.396830 | 0.347294 | 4.313355 |
| S | 1.364790 | -0.057205 | 2.523728 |
| C | 0.822801 | 0.822914 | 4.848006 |
| C | 1.867313 | 0.668721 | 3.990254 |
| O | -2.452899 | 1.733822 | -0.155865 |
| H | -0.884295 | -1.163966 | 1.269861 |
| H | -2.670974 | 0.977137 | 2.519853 |
| H | -5.427996 | 0.087979 | -1.325449 |
| H | -4.326710 | -2.203671 | 0.304940 |
| H | -2.156145 | -2.806332 | 2.618965 |
| H | -2.500318 | -1.552809 | 3.840255 |
| H | -3.932597 | 0.843822 | -1.910806 |
| H | -4.160442 | -0.910444 | -2.064972 |
| H | -1.341734 | 0.384955 | 4.843039 |
| H | 0.921075 | 1.261193 | 5.832506 |
| H | 2.902119 | 0.938064 | 4.137071 |

**$^{3}$ III**

25

scf done: -953.622704

| | | | |
|---|---|---|---|
| N | -3.811965 | -0.284777 | -0.035919 |
| C | -4.230117 | -1.040173 | 1.049455 |
| C | -3.209384 | -0.855490 | 2.081537 |
| C | -2.354306 | 0.325354 | 1.692319 |
| C | -2.714404 | 0.512531 | 0.226414 |
| C | -4.481837 | -0.291374 | -1.312800 |
| C | -2.006947 | -1.737414 | 2.304007 |
| C | -1.081502 | -0.537262 | 1.942687 |
| C | -0.178187 | -0.054772 | 3.017374 |
| C | -0.486649 | 0.233263 | 4.315157 |
| S | 1.492221 | 0.236316 | 2.718127 |
| C | 0.622878 | 0.685855 | 5.066099 |
| C | 1.767097 | 0.738313 | 4.331890 |
| O | -2.141962 | 1.203134 | -0.586796 |
| H | -0.518841 | -0.703477 | 1.020597 |
| H | -2.394309 | 1.280497 | 2.225077 |
| H | -5.554109 | -0.145790 | -1.167953 |
| H | -5.292124 | -1.224399 | 1.173130 |
| H | -1.942155 | -2.604305 | 1.639077 |
| H | -1.873524 | -2.087191 | 3.331799 |
| H | -4.083240 | 0.517503 | -1.921752 |
| H | -4.323939 | -1.240049 | -1.828689 |
| H | -1.487414 | 0.124627 | 4.718791 |
| H | 0.574138 | 0.961133 | 6.111571 |
| H | 2.753973 | 1.040904 | 4.647296 |

**$^{1}$ TS (III-IV)**

25

scf done: -953.669273

| | | | |
|---|---|---|---|
| N | 1.918130 | 1.851509 | 4.668440 |
| C | 1.287689 | 2.579118 | 3.656329 |
| C | 3.271635 | 2.047645 | 5.104329 |

| | | | |
|---|---|---|---|
| C | 1.026055 | 0.980298 | 5.274783 |
| O | 1.290487 | 0.251046 | 6.224990 |
| C | -0.238271 | 1.202982 | 4.577741 |
| H | -1.188009 | 0.911606 | 5.005230 |
| C | -0.021567 | 2.255780 | 3.625705 |
| H | 1.873688 | 3.179869 | 2.975638 |
| C | -1.015848 | 2.169322 | 2.525195 |
| H | -0.700830 | 2.614487 | 1.579239 |
| H | -1.989708 | 2.592694 | 2.791744 |
| C | -1.060545 | 0.670505 | 2.497695 |
| H | -0.164031 | 0.176957 | 2.134804 |
| C | -2.222224 | -0.113658 | 2.576553 |
| C | -2.320898 | -1.487389 | 2.420415 |
| C | -3.617207 | -1.974786 | 2.610180 |
| C | -4.512236 | -0.985112 | 2.915085 |
| S | -3.781272 | 0.555794 | 2.968254 |
| H | 3.399606 | 3.012909 | 5.602079 |
| H | 3.519844 | 1.256429 | 5.810460 |
| H | 3.961524 | 1.998908 | 4.258920 |
| H | -1.461755 | -2.100446 | 2.179042 |
| H | -3.893654 | -3.016977 | 2.525736 |
| H | -5.570679 | -1.083944 | 3.103705 |

**$^3$ TS (III-IV)**

25

scf done: -953.603266

| | | | |
|---|---|---|---|
| C | -1.141310 | -0.011956 | 0.585478 |
| C | -0.165839 | -0.154957 | 1.545349 |
| S | 1.393253 | 0.182825 | 0.857528 |
| C | 0.713776 | 0.526157 | -0.682870 |
| C | -0.640495 | 0.378913 | -0.671660 |
| C | -0.310554 | -0.683346 | 2.878691 |
| C | 0.745025 | -0.484517 | 3.978644 |
| C | 0.634476 | -1.921281 | 4.345404 |
| C | 0.119407 | -2.469904 | 3.114579 |
| C | -0.850917 | -3.501150 | 3.504131 |
| N | -0.925786 | -3.432201 | 4.922334 |
| C | -0.102382 | -2.525177 | 5.448003 |
| C | -1.792601 | -4.317275 | 5.662323 |
| O | -1.576775 | -4.234987 | 2.843907 |
| H | -1.327863 | -0.610085 | 3.262620 |
| H | 0.712838 | -2.629462 | 2.212557 |
| H | -1.679234 | -4.125679 | 6.726661 |
| H | 0.046792 | -2.437652 | 6.514068 |
| H | 1.720942 | -0.221626 | 3.556510 |
| H | 0.507054 | 0.241115 | 4.759881 |
| H | -2.828380 | -4.152314 | 5.365311 |
| H | -1.537384 | -5.354654 | 5.444721 |
| H | -2.189201 | -0.186396 | 0.797645 |
| H | -1.261491 | 0.554843 | -1.540698 |
| H | 1.351499 | 0.830370 | -1.498526 |

**$^1$ IV**

25
scf done: -953.696203

| | | | |
|---|---|---|---|
| C | 0.079940 | 0.803228 | 4.491133 |
| C | 0.180822 | 1.794434 | 3.547231 |
| C | 1.319950 | 2.554741 | 3.865306 |
| N | 1.906565 | 2.063307 | 4.977126 |
| C | 1.169820 | 0.944289 | 5.431575 |
| C | -0.722103 | 2.060172 | 2.381944 |
| C | -1.182108 | 0.825709 | 1.684124 |
| C | -0.363025 | 0.030352 | 0.912248 |
| S | 1.337762 | 0.386912 | 0.656802 |
| C | 1.531269 | -0.989284 | -0.357708 |
| C | 0.364968 | -1.695570 | -0.493633 |
| C | -0.700863 | -1.138147 | 0.212269 |
| C | 3.098411 | 2.560051 | 5.613871 |
| O | 1.470945 | 0.296186 | 6.421129 |
| H | -2.218593 | 0.518358 | 1.781738 |
| H | -0.663441 | 0.021973 | 4.550387 |
| H | 2.960217 | 3.585886 | 5.959122 |
| H | 1.726950 | 3.408027 | 3.339602 |
| H | -0.191747 | 2.715880 | 1.677561 |
| H | -1.591797 | 2.629692 | 2.721939 |
| H | 3.307065 | 1.923763 | 6.472209 |
| H | 3.949597 | 2.525642 | 4.932045 |
| H | -1.705582 | -1.541621 | 0.233003 |
| H | 0.289388 | -2.594252 | -1.091852 |
| H | 2.494829 | -1.205662 | -0.793494 |

**$^3$ IV**

25
scf done: -953.696591

| | | | |
|---|---|---|---|
| C | -0.361424 | 2.514777 | 4.927471 |
| C | -0.132928 | 1.723312 | 3.829285 |
| C | 1.192430 | 1.260329 | 3.922195 |
| N | 1.773677 | 1.751020 | 5.037170 |
| C | 0.841633 | 2.558837 | 5.728766 |
| C | -1.075378 | 1.419171 | 2.704710 |
| C | -0.811604 | 0.113691 | 2.043040 |
| C | -0.110687 | -0.050753 | 0.869993 |
| S | 0.564636 | 1.294985 | -0.039538 |
| C | 1.206029 | 0.236300 | -1.235648 |
| C | 0.926623 | -1.077077 | -0.963705 |
| C | 0.194728 | -1.252888 | 0.210119 |
| C | 3.115384 | 1.493666 | 5.491711 |
| O | 1.090939 | 3.128584 | 6.779437 |
| H | -1.154188 | -0.789690 | 2.540997 |
| H | -1.271677 | 3.033145 | 5.189764 |
| H | 3.848493 | 1.835701 | 4.759622 |
| H | 1.726680 | 0.606521 | 3.246065 |
| H | -1.020615 | 2.229323 | 1.966331 |
| H | -2.097417 | 1.444957 | 3.095896 |
| H | 3.262609 | 2.040089 | 6.421705 |
| H | 3.268695 | 0.429450 | 5.677956 |

| | | | |
|---|---|---|---|
| H | -0.125409 | -2.210941 | 0.600234 |
| H | 1.246023 | -1.889363 | -1.603606 |
| H | 1.751991 | 0.640923 | -2.074290 |

**TS (IV-V)**
25
scf done: -953.642557

| | | | |
|---|---|---|---|
| N | -0.007286 | 0.108340 | -0.011635 |
| C | 0.083206 | 0.182868 | 1.374582 |
| C | 1.484530 | -0.037327 | 1.695014 |
| C | 2.137285 | -0.357003 | 0.557104 |
| C | 1.207956 | -0.277088 | -0.564829 |
| C | -0.718511 | -1.335341 | 2.219705 |
| C | -0.288728 | -1.156380 | 3.570757 |
| C | 1.092989 | -0.998512 | 3.936563 |
| C | 1.950040 | -0.091157 | 3.099064 |
| S | -1.573610 | -0.482432 | 4.517381 |
| C | -2.720070 | -0.696989 | 3.243493 |
| C | -2.156057 | -1.193454 | 2.126268 |
| O | 1.385850 | -0.494319 | -1.753190 |
| C | -1.207312 | 0.301492 | -0.775971 |
| H | -1.798404 | -0.616473 | -0.855827 |
| H | -0.938404 | 0.619348 | -1.782790 |
| H | -1.823504 | 1.072224 | -0.310294 |
| H | -0.494295 | 0.967748 | 1.863046 |
| H | -0.210950 | -2.070069 | 1.592797 |
| H | 1.463416 | -1.314536 | 4.904436 |
| H | -3.763182 | -0.477282 | 3.418607 |
| H | 3.000146 | -0.390783 | 3.150287 |
| H | 1.901277 | 0.921341 | 3.534751 |
| H | 3.169861 | -0.656974 | 0.452320 |
| H | -2.718663 | -1.434074 | 1.233569 |

**V**
25
scf done: -953.653245

| | | | |
|---|---|---|---|
| C | 1.148676 | 6.637827 | 3.845209 |
| C | 0.311944 | 7.555799 | 4.670557 |
| C | 0.193050 | 8.835421 | 3.896304 |
| S | 1.372060 | 8.911678 | 2.618230 |
| C | 1.767357 | 7.219421 | 2.821339 |
| C | -0.347525 | 10.006824 | 4.540564 |
| C | 0.269195 | 10.369401 | 5.859199 |
| C | 0.352925 | 9.115634 | 6.656984 |
| C | 0.982366 | 7.901312 | 6.047952 |
| C | -0.131500 | 8.813946 | 7.859602 |
| C | 0.154385 | 7.399059 | 8.162219 |
| N | 0.809249 | 6.902697 | 7.065548 |
| C | 1.326811 | 5.567317 | 6.980875 |
| H | 2.366302 | 5.577901 | 6.642166 |
| O | -0.117410 | 6.770933 | 9.168866 |
| H | 0.744571 | 4.941230 | 6.297437 |
| H | 1.281762 | 5.120601 | 7.973518 |

| | | | |
|---|---|---|---|
| H | 2.050605 | 8.080430 | 5.844648 |
| H | -0.674462 | 7.134407 | 4.918147 |
| H | -1.311935 | 10.423693 | 4.266562 |
| H | 2.447839 | 6.755216 | 2.120984 |
| H | -0.298167 | 11.135980 | 6.388436 |
| H | 1.286417 | 10.758168 | 5.696543 |
| H | -0.657722 | 9.465054 | 8.543229 |
| H | 1.269770 | 5.589118 | 4.086531 |

**TS (IV-2)**

25

scf done: -953.680979

| | | | |
|---|---|---|---|
| N | 3.957856 | -1.273946 | 1.736046 |
| C | 2.665538 | -1.182950 | 1.336918 |
| C | 2.597302 | -0.262617 | 0.252674 |
| C | 3.866572 | 0.158242 | -0.019068 |
| C | 4.774624 | -0.452126 | 0.926396 |
| C | 1.338225 | 0.154015 | -0.451659 |
| C | 0.219839 | -0.712291 | 0.001715 |
| C | -0.083349 | -0.716399 | 1.324782 |
| C | 0.609449 | 0.023977 | 2.325540 |
| C | 0.239595 | -0.355839 | 3.630075 |
| C | -0.690374 | -1.346783 | 3.654474 |
| S | -1.174738 | -1.871240 | 2.087198 |
| O | 5.975065 | -0.318996 | 1.097277 |
| C | 4.450592 | -2.102909 | 2.802602 |
| H | 1.164864 | 0.922880 | 2.102522 |
| H | 1.958626 | -1.939765 | 1.632815 |
| H | 4.265284 | -3.160066 | 2.600198 |
| H | 3.979087 | -1.837661 | 3.751145 |
| H | 5.524235 | -1.941100 | 2.884193 |
| H | -0.296007 | -1.358996 | -0.696774 |
| H | -1.145563 | -1.793795 | 4.526311 |
| H | 4.172316 | 0.887394 | -0.754374 |
| H | 0.634943 | 0.103937 | 4.525757 |
| H | 1.482009 | 0.076525 | -1.531063 |
| H | 1.145959 | 1.217501 | -0.247115 |

**2**

25

scf done: -953.741547

| | | | |
|---|---|---|---|
| C | 1.165480 | 6.650338 | 3.873626 |
| C | 0.432371 | 7.631466 | 4.735366 |
| C | 0.376374 | 8.919487 | 3.947068 |
| S | 0.663576 | 8.615321 | 2.226490 |
| C | 1.297677 | 7.019496 | 2.606733 |
| C | 0.228012 | 10.124794 | 4.482776 |
| C | 0.082647 | 10.334721 | 5.957609 |
| C | 0.223405 | 9.065687 | 6.714608 |
| C | 1.006785 | 7.932454 | 6.122369 |
| C | -0.308373 | 8.683526 | 7.871595 |
| C | 0.098979 | 7.297394 | 8.167067 |
| N | 0.847432 | 6.885855 | 7.096139 |

| | | | |
|---|---|---|---|
| C | 1.535400 | 5.626005 | 7.049903 |
| H | 2.571174 | 5.767452 | 6.730587 |
| O | -0.158038 | 6.627332 | 9.149822 |
| H | 1.055602 | 4.914861 | 6.371091 |
| H | 1.528916 | 5.195822 | 8.051071 |
| H | 2.068645 | 8.202191 | 6.008430 |
| H | -0.592335 | 7.255571 | 4.898533 |
| H | 0.236433 | 11.008257 | 3.853108 |
| H | 1.728710 | 6.435023 | 1.805427 |
| H | -0.871811 | 10.813813 | 6.201175 |
| H | 0.851594 | 11.046056 | 6.287374 |
| H | -0.953346 | 9.254515 | 8.524585 |
| H | 1.481248 | 5.681632 | 4.239100 |

# Comprehensive Table in AU

| M06/def2-TZVP<br>CPCM = DCM | H (hartrees) | S (cal/K*mol) | imag. freq. (1/cm) |
|---|---|---|---|
| **1** | -953.497142 | 124.563 | |
| **I (triplet on allene)** | -953.430997 | 125.497 | |
| **I' (triplet on alkene)** | -953.422908 | 127.675 | |
| **TS (I-II)** | -953.428770 | 119.348 | -231.0468 |
| **II** | -953.492264 | 121.253 | |
| **$^{1}$ III** | -953.510536 | 113.431 | |
| **$^{3}$ III** | -953.429974 | 117.888 | |
| **$^{1}$ TS (III-IV)** | -953.477120 | 112.755 | -558.0511 |
| **$^{3}$ TS (III-IV)** | -953.413271 | 116.322 | -840.5435 |
| **$^{1}$ IV** | -953.504603 | 120.285 | |
| **$^{3}$ IV** | -953.505037 | 123.948 | |
| **TS (IV-V)** | -953.451373 | 112.324 | -530.2581 |
| **V** | -953.459504 | 110.672 | |
| **TS (IV-2)** | -953.487623 | 108.639 | -78.5296 |
| **2** | -953.544601 | 107.556 | |
